# Ultralow‐Dimensionality Reduction for Identifying Critical Transitions by Spatial‐Temporal PCA

**DOI:** 10.1002/advs.202408173

**Published:** 2025-04-25

**Authors:** Pei Chen, Yaofang Suo, Kazuyuki Aihara, Ye Li, Dan Wu, Rui Liu, Luonan Chen

**Affiliations:** ^1^ School of Mathematics South China University of Technology Guangzhou 510640 China; ^2^ International Research Center for Neurointelligence Institutes for Advanced Study The University of Tokyo Tokyo 113‐0033 Japan; ^3^ Shenzhen Institutes of Advanced Technology Chinese Academy of Sciences Shenzhen 518055 China; ^4^ School of Mathematical Sciences School of AI Shanghai Jiao Tong University Shanghai 200240 China; ^5^ Key Laboratory of Systems Biology Hangzhou Institute for Advanced Study University of Chinese Academy of Sciences Chinese Academy of Sciences Hangzhou 310024 China

**Keywords:** critical state transition, interpretable data representation, spatial‐temporal PCA, ultralow‐dimensionality reduction

## Abstract

Discovering dominant patterns and exploring dynamic behaviors especially critical state transitions and tipping points in high‐dimensional time‐series data are challenging tasks in study of real‐world complex systems, which demand interpretable data representations to facilitate comprehension of both spatial and temporal information within the original data space. This study proposes a general and analytical ultralow‐dimensionality reduction method for dynamical systems named spatial‐temporal principal component analysis (stPCA) to fully represent the dynamics of a high‐dimensional time‐series by only a single latent variable without distortion, which transforms high‐dimensional spatial information into one‐dimensional temporal information based on nonlinear delay‐embedding theory. The dynamics of this single variable is analytically solved and theoretically preserves the temporal property of original high‐dimensional time‐series, thereby accurately and reliably identifying the tipping point before an upcoming critical transition. Its applications to real‐world datasets such as individual‐specific heterogeneous ICU records demonstrate the effectiveness of stPCA, which quantitatively and robustly provides the early‐warning signals of the critical/tipping state on each patient.

## Graphical abstract

The proposed spatial‐temporal principal component analysis (stPCA) method analytically reduces high‐dimensional time‐series data to a single latent variable by transforming spatial information into temporal dynamics. By preserving the temporal properties of the original data, stPCA effectively identifies critical transitions and tipping points. It provides robust early‐warning signals, demonstrating effectiveness on both simulation and real‐world datasets.

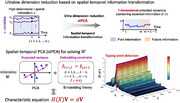

## 1 Introduction

Numerous physical and biological processes are characterized by high‐dimensional nonlinear dynamical systems, in which critical state transitions often occur.^[^ ^1^ ^]^ However, for most real‐world systems that are too complex to be simply described by an explicit model, we have to study their dynamics especially identifying the tipping point before an upcoming critical transition through high‐dimensional time‐series or sequential data. The exploratory analysis of these systems requires data dimensionality reduction and linear or nonlinear data representations,^[^ ^2^ ^]^ which are critical in dynamical analysis, pattern recognition, and visualization.^[^ ^3^ ^]^ It is still a challenging problem to efficiently perform dimensionality reduction and explore ultralow‐dimensional dynamics in an interpretable way for complex dynamical systems. A variety of data‐driven techniques have been developed to facilitate this task, including t‐distributed stochastic neighbor embedding (t‐SNE),^[^ ^4^ ^]^ uniform manifold approximation and projection,^[^ ^5^ ^]^ multidimensional scaling (MDS),^[^ ^6^ ^]^ local linear embedding (LLE),^[^ ^7^ ^]^ isometric feature mapping (ISOMAP).^[^ ^8^ ^]^ Nevertheless, these approaches generally lack the desirable linear and temporal interpretability, which is essential in enabling a more lucid depiction of location, direction, distance, and dynamics in the low‐dimensional representation space.

Linear dimensionality reduction methods, including principal component analysis (PCA),^[^ ^9^ ^]^ linear discriminant analysis,^[^ ^10^ ^]^ sparse dictionary learning (SDL),^[^ ^11^ ^]^ and independent component analysis^[^ ^12^ ^]^ offer interpretability by preserving the relations among variables using linear transformations. For example, PCA projects the original data onto a lower‐dimensional subspace in a linear form such that the projected latent variables capture the maximum variance in the representation space. However, these existing methods do not leverage the temporal or inherent relations among sequentially observed samples within the original data; hence, explaining the dynamic nature of the time‐series data by these methods is difficult. Besides, these methods are sensitive to outliers and thereby susceptible to noise interference. These limitations are a barrier to the practical usage of data representation for real‐world dynamical systems.

Deep neural networks (DNNs) have emerged as powerful tools in sequential data analysis and dynamic modeling, leveraging deep learning techniques to handle nonlinear embedding and intricate patterns in dynamical systems, including recurrent neural networks (RNNs)^[^ ^13^ ^]^ and the low‐rank variants,^[^ ^14^, ^15^ ^]^ temporal convolutional networks,^[^ ^16^ ^]^ autoencoders (AEs),^[^ ^17^ ^]^ deep belief networks (DBNs),^[^ ^18^ ^]^ transformers,^[^ ^19^ ^]^ CEBRA,^[^ ^20^ ^]^ LFADS,^[^ ^21^ ^]^ CANDyMan,^[^ ^22^ ^]^ and MARBLE.^[^ ^23^ ^]^ Despite of the state‐of‐the‐art performances of these DNN architectures, traditional RNNs can be computationally intensive and may suffer from issues such as vanishing or exploding gradients, which can affect their ability to capture all relevant information. Additionally, many DNN architectures still demand significant computational and memory resources, which can be limiting in resource‐constrained environments or scenarios with strict latency requirements.

Recently, the Koopman operator theory offers an alternative perspective, suggesting that linear superposition may characterize nonlinear dynamics through the infinite‐dimensional linear Koopman operator,^[^ ^24^, ^25^, ^26^, ^27^ ^]^ but obtaining its representations has proven difficult in all but the simplest systems. One approach to find tractable finite‐dimensional representations of the Koopman operator is DNN,^[^ ^28^ ^]^ however, they still face challenges related to the requirement of large training samples and other computational issues. Dynamic mode decomposition (DMD)^[^ ^24^, ^25^, ^26^ ^]^ is another commonly employed method for approximating the Koopman operator from time‐series data. It decomposes the system dynamics into temporal modes, each of which is associated with a fixed oscillation frequency and decay/growth rate. Some variants of the DMD algorithm mitigate noise interference by using techniques such as a Schweppe‐type Huber maximum‐likelihood estimator to flag outliers, incorporating regularizers and constraints  on the decomposition parameters, or deriving a maximum a posteriori (MAP) estimator for systems corrupted by multiplicative noise.^[^ ^29^, ^30^, ^31^ ^]^ Nonetheless, these DMD methods may require extensive time‐series data, and involves the computation of pseudo‐inverses of linear matrix operator and truncation of spectrum, all potentially impacting accuracy. Another method for representing dynamical systems is sparse identification of nonlinear dynamical systems,^[^ ^32^ ^]^ which combines sparsity‐promoting techniques and machine learning with nonlinear dynamical systems to discover governing equations, but the accuracy highly depends on the choice of measurement coordinates and the sparsifying function.

Furthermore, these dimensionality reduction methods mentioned above generally require multiple variables or principal components to represent the original high‐dimensional data. In other words, multiple representation components are usually mandatory for reconstructing a dynamical system by those methods to avoid information loss, which leads to challenges in efficiently visualizing or quantifying nonlinear phenomena, e.g., the critical slowing down (CSD) effect.^[^ ^33^, ^34^ ^]^ In fact, when a dynamical system approaches a bifurcation point from a stable steady state, the space is generically constrained to a center manifold, which typically has codim‐1 local bifurcation with a dominant real eigenvalue such as the saddle‐node bifurcation; thus, this one‐dimensional variable is roughly considered to represent the center manifold near the tipping point. A natural question arises for analyzing the observed high‐dimensional time‐series data: is it possible to devise an ultralow‐dimensionality reduction method with only a single latent variable to fully represent or reconstruct the whole dynamics of the original, yet unknown, high‐dimensional system?

Based on the generalized Takens’ embedding theory,^[^ ^35^, ^36^ ^]^ the dynamics of a system can be topologically reconstructed from the delay embedding scheme if *L* > 2*d* > 0, where *d* is the box‐counting dimension of the attractor for the original system and *L* represents the embedding dimension. By assuming that the steady state of a high‐dimensional system is contained in a low‐dimensional manifold, which is generally satisfied for dissipative real‐world systems, the spatial‐temporal information (STI)^[^ ^37^ ^]^ transformation has theoretically been derived from the delay embedding theory. This approach transforms the spatial information of high‐dimensional data into the temporal dynamics of any (one) target variable, i.e., a one‐dimensional delay embedding of this single variable, thus exploiting not only inter‐sample (cross‐sample) information but also intra‐sample information embedded in the high‐dimensional/spatial data. Several methods, such as the randomly distributed embedding,^[^ ^37^ ^]^ the autoreservoir neural network,^[^ ^38^ ^]^ and the spatiotemporal information conversion machine,^[^ ^39^ ^]^ have been developed for predicting short‐term time series within the framework of STI transformation, which depends on an explicit target variable from high‐dimensional data.

**Figure 1:**
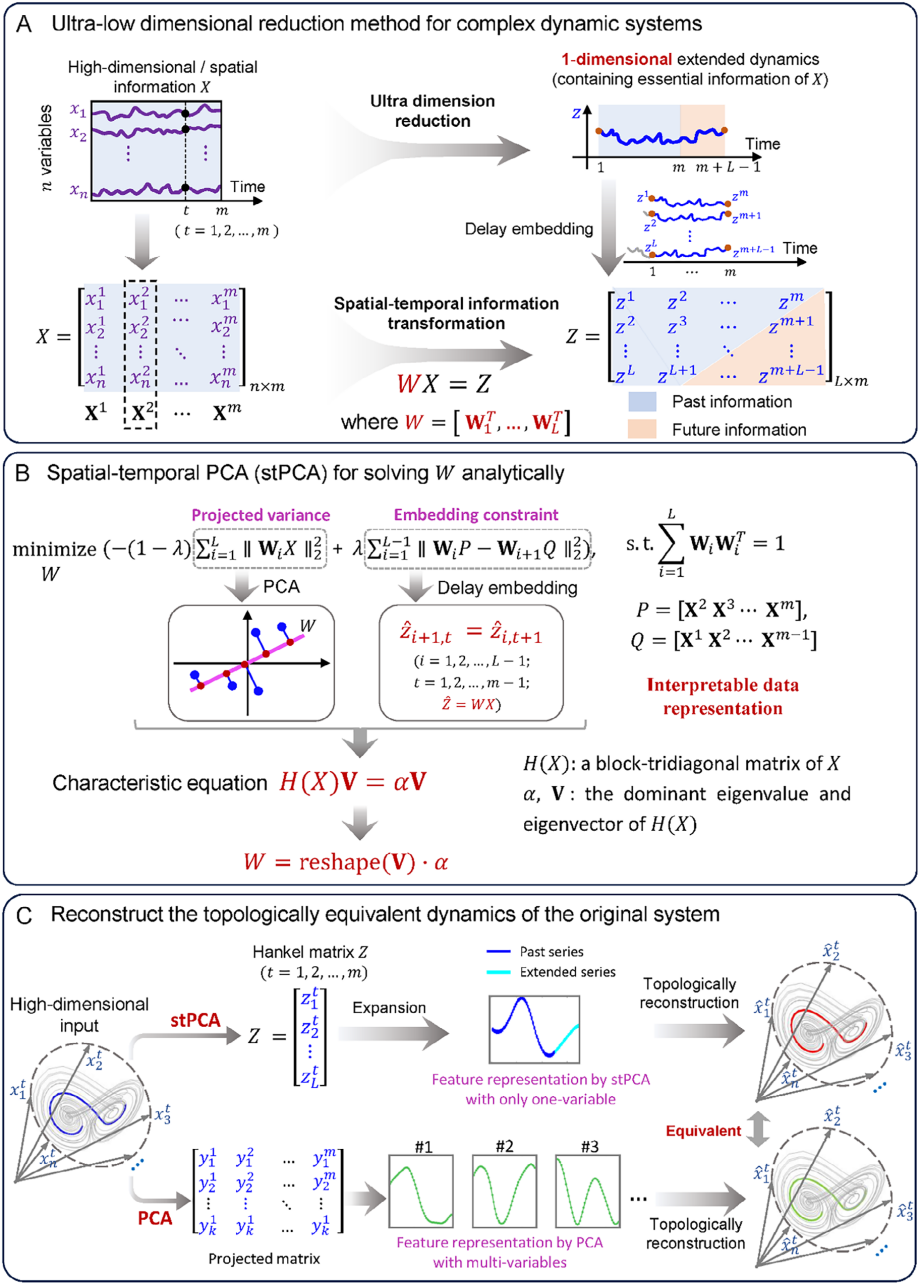
Schematic illustration of stPCA. A) The given high‐dimensional “spatial” information **X** ^*t*^ can be transformed into one‐dimensional “temporal” information **Z** ^*t*^ based on the STI equations derived from the delay‐embedding theorem. B) Given the high‐dimensional time series *X*, stPCA seeks to obtain the transformation matrix *W* and the projected Hankel matrix *Z*. The optimization objective consists of two terms: the first term aims to maximize the variance of the projected variable *Z*, and the second term ensures that the projected Hankel matrix *Z* satisfies the delay embedding condition. The analytical solution to the optimization problem is obtained by solving a characteristic equation *H*(*X*)V = αV, where *H*(*X*) is a block‐tridiagonal matrix. C) Through stPCA, the high‐dimensional time series *X* is effectively reduced to the Hankel matrix *Z*, which is actually formed by a single variable. On the other hand, PCA is employed to reduce the high‐dimensional time series *X* to *Y*. Notably, both the single latent variable *z* from stPCA and multiple latent variables from PCA can topologically reconstruct the original dynamical system.

In this study, according to the STI transformation but relying on a latent variable, we develop a novel ultralow‐dimensionality reduction framework: spatial‐temporal principal component analysis (stPCA), to fully represent the dynamics of high‐dimensional time‐series data by only a single latent variable. This framework enables effective detection of the impending critical transitions in dynamical systems with codim‐1 local bifurcation by examining the fluctuation of this single variable derived by transforming high‐dimensional spatial information into low‐dimensional temporal information. On the basis of a solid theoretical background in nonlinear dynamics and delay embedding theory (**Figure** **1**A), stPCA naturally imposes constraints on samples from the inherent temporal characteristics of dynamical systems, rendering it an applicable method in representing a complex dynamical system with almost no information loss. Furthermore, this single representation variable is analytically obtained from a delay embedding‐based optimization problem by solving a characteristic equation, rather than using an iterative numerical optimization algorithm which generally depends on initial values of parameters *W*,^[^ ^40^ ^]^ and can theoretically preserve the dynamical properties of the original high‐dimensional system.

Specifically, stPCA employs a spatial‐temporal transformation denoted as *W*, merging i) the attributes of linear interpretation from PCA and ii) the temporal dynamics from delay embeddings (Figure 1B). On one hand, similar to PCA, stPCA requires the maximum projected variance to attain a proper feature transformation (i.e., explainable features), ensuring the capability of a latent variable to quantify critical state transitions based on CSD theory; on the other hand, it requires the projected latent variable to adhere to the delay embedding constraints (i.e., temporal features);^[^ ^38^, ^39^ ^]^ thus, this ultralow 1‐dimensional variable can be theoretically used to topologically reconstruct the original high‐dimensional dynamical system. Therefore, it can be obtained by simultaneously optimizing the projected variance and the consistency with the delay embedding for the representative data. The existence of a closed‐form solution makes stPCA analytically tractable and offers a fast computational way to even large datasets.

Owing to the single representative variable of stPCA, it may become convenient to explore, quantify, and even predict the short‐term nonlinear phenomena/behaviors of real‐world dynamical systems from the observed high‐dimensional time‐series data, e.g., the deterioration of diseases,^[^ ^41^ ^]^ climate changes,^[^ ^42^ ^]^ and the occurrences of strong earthquakes.^[^ ^43^ ^]^ In particular, such a single representative variable derived from stPCA can be directly utilized to detect early‐warning signals of a critical state or tipping point just before a sudden event based on the CSD effect (a state transition condition).^[^ ^33^, ^41^, ^44^ ^]^ We applied stPCA first to three representative models, including coupled Lorenz systems,^[^ ^45^, ^46^ ^]^ multiple‐node networks with Michaelis‒Menten forms,^[^ ^47^ ^]^ and a DREAM4 dataset.^[^ ^48^ ^]^ Subsequently, we extended the analysis to real‐world scenarios, i.e., the Medical Information Mart for both Intensive Care III (MIMIC‐III)^[^ ^49^ ^]^ and Intensive Care IV (MIMIC‐IV)^[^ ^50^ ^]^ datasets, with the aim of identifying the tipping points of patients during their ICU hospitalizations. With the dynamics of a single variable derived by stPCA from heterogeneous high‐dimensional ICU data, we proposed a decision‐making scheme for ICU discharge with the goal of improving patient survival rates and ICU performance. In addition, stPCA was utilized to analyze a single‐cell RNA sequencing dataset of human embryonic stem cells,^[^ ^51^ ^]^ successfully detecting the cell fate transition of differentiation into definitive endoderm. Finally, we predicted the risk of transitioning from a healthy state to type 2 diabetes (T2D) based on continuous glucose monitoring data.^[^ ^52^ ^]^ All of these results validated the effectiveness and efficiency of the proposed stPCA, as a new ultralow‐dimensionality reduction tool for dynamically decoding high‐dimensional time series.

## 2 Results

### 2.1 The stPCA Framework for High‐Dimensional Time‐Series Data: an Analytical and Explainable Approach of a Dynamical System

The proposed stPCA framework is a dynamic dimensionality reduction method designed to maintain the interpretability of a linear subspace while representing a high‐dimensional dynamical system with a single projected variable using a delay embedding strategy. For an observed high‐dimensional time series $X^{t}={\left(x_{1}^{t},x_{2}^{t},\text{…},x_{n}^{t}\right)}^{\prime }$ with *n* variables and t=1,2,…,m, we construct a corresponding delayed vector **Z** ^*t*^ = ($Z^{t}={\left(z^{t},z^{t+1},\text{…},z^{t+L-1}\right)}^{\prime }$ by a delay‐embedding strategy with *L* > 1 as the embedding dimension (Figure 1A), where the symbol “ ′ ” represents the transpose of a vector. Clearly, **X** ^*t*^ is a spatial vector with *n* variables observed at one‐time point *t*, while **Z** ^*t*^ is a temporal vector formed by only one variable *z* but at many time points t,t+1,…,t+L−1. According to Takens’ embedding theory and its generalized versions, such as a delay‐embedding scheme, **Z** ^*t*^ topologically reconstructs the equivalent dynamics of the original system **X** ^*t*^ if *L* > 2*d* > 0, where *d* is the box‐counting dimension of the attractor of **X** ^*t*^, via the spatial‐temporal information transformation (STI) equations Φ (**X** ^*t*^) = **Z** ^*t*^ shown in the Materials and Methods section. In this way, $Z=[Z^{1},Z^{2},\text{…},Z^{m}]$ is a Hankel matrix formed by an extended vector $z=(z^{1},\;z^{2},\text{…},z^{m},\;z^{m+1},\text{…},z^{m+L-1})$ of a single variable. Then, aiming to identify the critical state transition of a complex dynamical system, we linearize the STI equation as *Z* = *WX*. Based on the delay embedding requirement for *Z*, the latter *m* − 1 elements in the *i*‐th row are identical to the former *m* − 1 elements in the (*i* + 1)‐th row of the matrix *Z*, thus we can establish the following equation:

(1) $$W_{i}P=W_{i+1}\;Q$$

where *P* and *Q* are two submatrices of *X*, i.e., *P* = [**X** ^2^ **X** ^3^ ⋅⋅⋅ **X** ^*m*^], *Q* = [**X** ^1^ **X** ^2^ ⋅⋅⋅ **X** ^*m* − 1^], and **W**_*i*_ = (*w*_*i*1_, *w*_*i*2_,⋅⋅⋅, *w_in_*), *i* = 1, 2, ⋅⋅⋅, *L* − 1 (see the Experimental Section for details). More explanations about linear embedding are provided in the Discussion section.

On the other hand, to obtain *W* such that *Z* can be obtained through *Z* = *WX* given *X* = [**X** ^1^ **X** ^2^ ⋅⋅⋅ **X** ^*m*^] with *n* variables {$x_{1}^{t},x_{2}^{t},\text{…},x_{n}^{t}$}, i.e., **Z**_*i*_ = **W**_*i*_ *X*, it is necessary to minimize the reconstruction error or maximize the variance of the projected variable $∥Z_{i}{∥}_{2}^{2}$. Then, the sample mean of *X* is removed as is done in PCA. Thus, the loss function is shown as

(2) $$\underset{W}{\mathrm{minimize}\;\;}-\left(1-\lambda \right)\sum_{i=1}^{L}∥W_{i}{X∥}_{2}^{2}+\lambda \sum_{i=1}^{L-1}{∥W_{i}P-W_{i+1}Q∥}_{2}^{2},$$

(3) $$s.t.\;\;\sum_{i=1}^{L}W_{i}W_{i}^{T}=1$$

where λ is a predetermined regularization parameter between 0 and 1. The first term of Equation (2) is the typical PCA loss, which guarantees the maximum projected variance and enables identification of the critical state transition by examining the fluctuation of **z**, as detailed in the Materials and Methods section. The second term makes *Z* a Hankel matrix, i.e., governed by the delay embedding constraint according to Equation (1). Theoretically, the obtained single variable *z^t^* can be used to topologically reconstruct the dynamics of the original system **X** ^*t*^, thus characterizing the dynamical features of the whole original system.

Given *X*, in this work, an analytical solution to the optimization problem in Equation (2) was presented by solving the following characteristic equation:

(4) $$H\left(X\right)V=\alpha V$$

where *H* is a function that converts *X* to a block‐tridiagonal matrix, α and **V** denote the dominant eigenvalue and a corresponding eigenvector of matrix *H*(*X*), respectively. Matrix *W* is obtained by reshaping vector **V** (Figure 1B), then leading to Hankel matrix *Z* (Figure 1A). The main time cost comes from solving the dominant eigenvalue of *H*(*X*). Utilizing the tridiagonal matrix algorithm,^[^ ^53^ ^]^ its time complexity is *O*(*nL*), which ensures the high efficiency of the stPCA algorithm.

### 2.2 Ultralow‐Dimensionality Data Representation and High Dynamic Robustness: Application of stPCA to Time‐Series Data of a Coupled Lorenz Model

To illustrate the mechanism of the proposed framework, a series of coupled Lorenz models^[^ ^45^, ^46^ ^]^

(5) $$\dot{X}\left(t\right)=G\left(X\left(t\right);P\right)$$

were employed to generate synthetic time‐series datasets under different noise conditions, where *G*(·) is the nonlinear function of the Lorenz system with $X\left(t\right)={\left(x_{1}^{t},\text{…},x_{n}^{t}\right)}^{\prime }$, **P** is a parameter vector, and *n* is the dimension of **X**(*t*). More details about the Lorenz system are provided in Note S4 (Supporting Information).

To study the intrinsic dynamical information of variable *z* via stPCA, singular value decomposition (SVD) is carried out on the Hankel matrix *Z* stacked by *z*:

(6) $$Z={\left(\begin{array}{cccc} z^{1} & \;z^{2} & \;\text{…} & \;z^{m}\\ z^{2} & \;z^{3} & \;\text{…} & \;z^{m+1}\\ \vdots & \;\vdots & \;\ddots & \;\vdots \\ z^{L} & \;z^{L+1} & \;\text{…} & \;z^{m+L-1} \end{array}\right)}_{L\times m}=U\Sigma R$$

**Figure 2:**
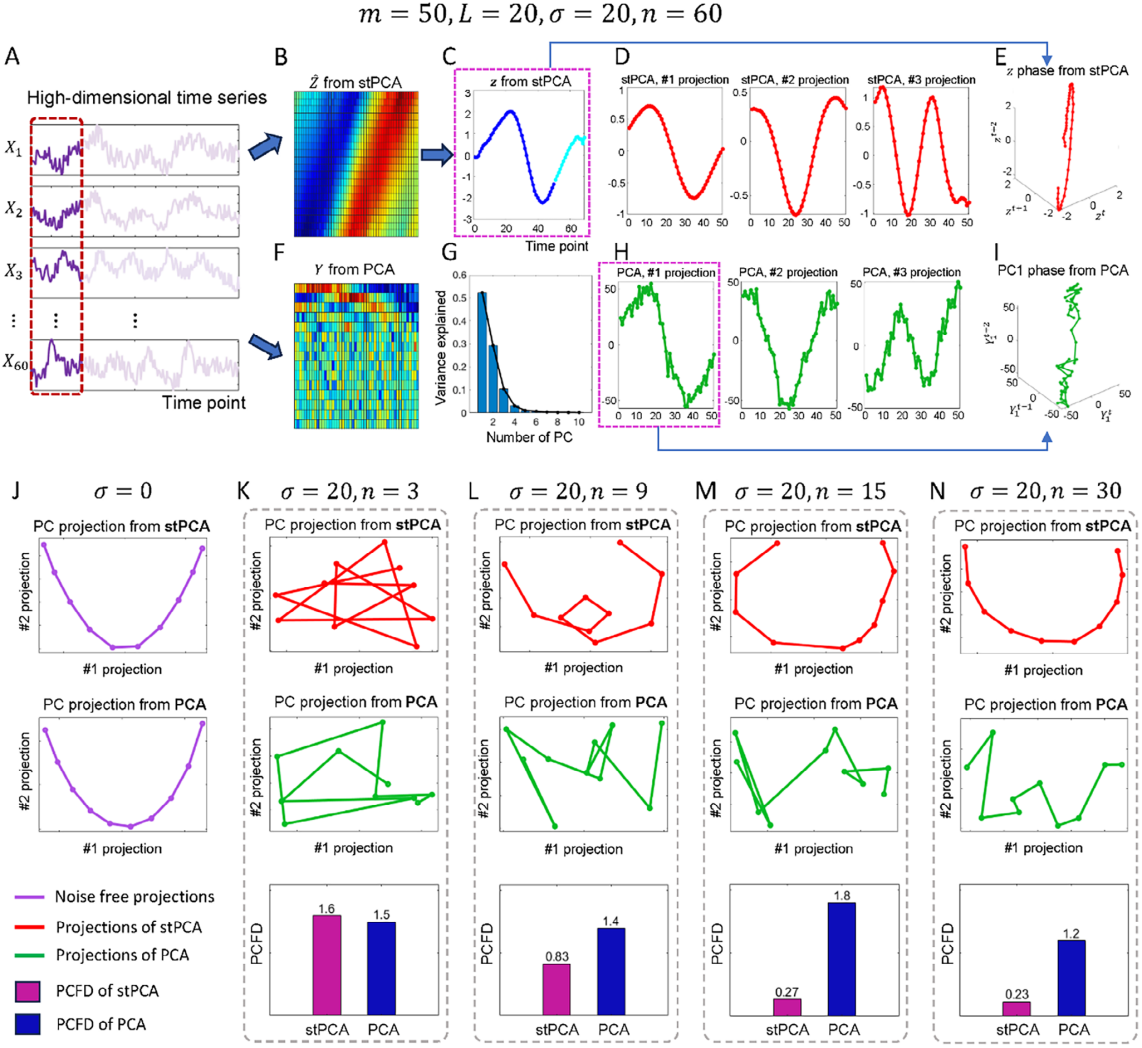
Synthetic time‐series datasets of a coupled Lorenz model were generated in noise‐free and noisy situations. A–I) For the strong noise‐perturbed scenario (noise intensity σ = 20), the dimensionality reduction results of a time series with length *m* = 50 and embedding dimension *L* = 20 of the 60D‐coupled (*n* = 60) Lorenz system. A) The original high‐dimensional time series *X*. B) The heatmap of the matrix $\hat{Z}$ obtained by stPCA. C) The one‐dimensional variable *z* obtained by stPCA. D) Projections of the top three principal components (denoted as PC for short in the figure) obtained from the Hankel matrix from stPCA. E) The projected phase of the one‐dimensional variable *z*. F) The heatmap of the projections *Y* of *X* obtained by PCA. G) The proportions of PC variances from PCA. H) Projections of the top three PCs from PCA. I) The projected phase of the #1 PC projection from PCA. J) The projections of the top 2 PCs from stPCA or PCA for a low‐dimensional system (*n* = 3) with *m* = 10, *L* = 5 and noise intensity σ = 0. K–N) For dimensionalities *n* = 3, 9, 15, and 30, the curves of top 2 PC projections from stPCA and PCA, along with the PCFD between the curve in Figure 2J and that in the strong noise‐perturbed scenario by stPCA and PCA, respectively.

The columns of matrices *U* and *R* after SVD are structured hierarchically according to their capability to represent the columns and rows of *Z*. Usually, *Z* can be derived via a low‐rank approximation by utilizing the first *r* columns from *U* and *R*.^[^ ^20^, ^21^ ^]^ The initial *r* columns of *R* represent a time series of the intensity of each column of *U*Σ.^[^ ^54^ ^]^ In this manner, we can decompose the single variable *z* into multiple principal components projections (**Figure** **2**C,D).

To quantify the similarity between noisy cases and noise‐free cases for the principal component projections of the stPCA and PCA algorithms respectively, we designed a metric called the principal component Frechet distance (PCFD) based on the Frechet distance^[^ ^55^, ^56^ ^]^ (Note S7 for details, Supporting Information). Given a metric space (H,D) with *D* as the distance metric in H, for two curves $\;y:[b_{1},\;b_{2}]\to H$ and $\;z:\;[b_{1}^{\prime },\;b_{2}^{\prime }]\to H$, their discrete Fréchet distance (DFD) is given as

(7) $$\mathrm{DFD}\left(y,z\right)=\underset{\beta_{y},\beta_{z}}{\inf}\max_{t\in \left[0,1\right]}D\left(y\left(\beta_{y}\left(t\right)\right),z\left(\beta_{z}\left(t\right)\right)\right)$$

where β_*y*_ and β_*z*_ are arbitrary continuous nondecreasing functions from [0, 1] onto [*b*_1_, *b*_2_] and $\left[b_{1}^{\prime },\;b_{2}^{\prime }\right]$, respectively. To better evaluate the distance of the two projection trajectories, we normalize the curves/projections by $\tilde{y}=\;\mathrm{Normalize}\left(y\right)$ and $\tilde{z}=\;\mathrm{Normalize}\left(z\right)$. Any commonly used normalization methods, such as the z‐score, can be applied. Then

(8) $$\mathrm{PCFD}\;\left(y,z\right)=\;\min\left\{\mathrm{DFD}\left(\tilde{y},\tilde{z}\right),\mathrm{DFD}\left(-\tilde{y},\tilde{z}\right)\right\}$$

By plotting the projections of the first three columns of matrix *R* in Equation (6) of one noise‐free case (*m* = 50, *L* = 20, *n* = 20, σ = 0), we found that the projections of the top principal components from *Z* by stPCA are very similar to those by PCA (Figure S5, Supporting Information), with PCFD values of 0.108, 0.291 and 0.254 for the top 3 components, respectively, which indicates that the 1‐dimensional variable *z* actually contains as much information as multiple projected variables from PCA. This characteristic is evidence that our stPCA method achieves efficient dimensionality reduction to an ultralow‐dimensional space with almost no information loss compared with traditional PCA. The analysis results obtained by stPCA and PCA for more cases are presented in Figure S1 (Supporting Information). Furthermore, both stPCA and PCA were applied in two scenarios: one with the original sample order and another with a randomly shuffled sample order, as shown in Figure S2 (Supporting Information).

In addition, we discuss the situation when there is additive noise, where *n* = 60, *m* = 50, *L* = 20, and noise intensity σ = 20 (Figure 2A). The results are illustrated in Figure 2B–I. The projections of the top three principal components from stPCA in the presence of noise closely resemble those from the noise‐free situation, which are shown in Figure S5 (Supporting Information). Besides, Figure 2D,H demonstrates the high robustness of stPCA against noise in contrast to PCA, which may be due to the inherent temporal constraints of stPCA imposed by the dynamical system in a steady state. The results of more cases with different noise intensities are illustrated in Figure S5 (Supporting Information). The quantified PCFD values for different embedding dimensions *L* are presented in Table S1 (Supporting Information).

We also applied stPCA and PCA to short‐term time series with *m* = 10 and *L* = 5. With the relatively low dimensionality *n* = 3 and a noise intensity σ = 0 of system Equation (5), the projections of the top two principal components obtained from stPCA and PCA are illustrated in Figure 2J. When strong noise is introduced (σ = 20), we illustrated the top two principal components for dimensionalities *n* = 3, 9, 15, and 30 in Figure 2K–N. Clearly, with low dimensionality (*n* = 3), both stPCA and PCA exhibit unsatisfactory performance due to the presence of noise compared with the noise‐free situation (Figure 2K). As dimensionality increases, the #1‐#2‐projection curve obtained from stPCA becomes smoother, and the PCFD between this curve and that of stPCA in Figure 2J decreases from 1.6 to 0.23 (Figure 2L–N). Meanwhile, the projection curve from PCA always exhibits irregular fluctuations owing to noise perturbations. The improved performance of stPCA with the increasement of dimensionality of system Equation (5) indicates that the inclusion of additional relevant variables results in an expansion of spatial information. According to Takens' embedding theory, such extra/increased spatial information can be transformed into temporal information, thereby enhancing the robustness of stPCA to noise, which also demonstrates the ability of stPCA in converting spatiotemporal information. More results are demonstrated in Figure S3 (Supporting Information). We also applied stPCA to longer time series of a coupled Lorenz system and the DREAM4 dataset, and the results are shown in Figures S4 and S7 (Supporting Information).

### 2.3 The Early‐Warning Signals of a Critical Transition: Application of stPCA to the Tipping Point Detection of Multiple‐Node Networks

**Figure 3:**
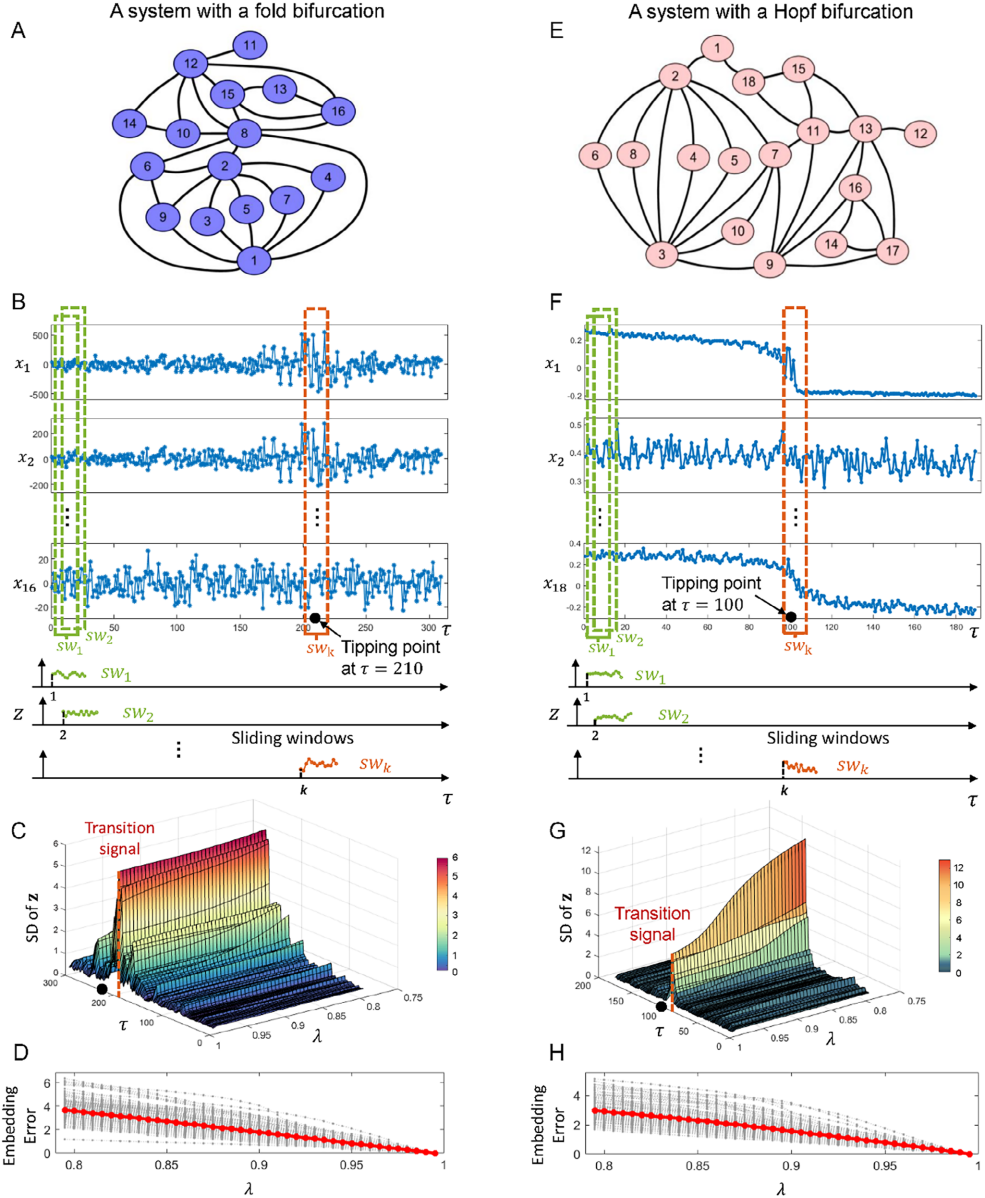
Application of stPCA to the tipping point detection in simulated datasets. The datasets are generated from a multiple‐node network model. A) A 16‐node model of a 16‐dimensional dynamical system with a Hopf bifurcation. B) The observed dynamics of each node/variable as parameter τ varies for the 16‐node model. To capture the early‐warning signals of the tipping points before the critical transitions of the dynamical system, the original high‐dimensional time series are partitioned into sliding windows. The one‐dimensional latent variable *z* is obtained by stPCA from each sliding window. C) In each sliding window, the standard deviation (SD) of the latent variable *z* is calculated with the varying parameter τ and regularization parameter λ for the 16‐node model. D) The red and gray curves represent the average embedding error and all embedding errors as λ varies, respectively. E) An 18‐node model of an 18‐dimensional dynamical system with a fold bifurcation. F) The observed dynamics of each node/variable as parameter τ varies for the 18‐node model. The one‐dimensional latent variable *z* is obtained by stPCA from each sliding window. G) The SD of *z* is calculated within a sliding window as parameters τ and λ varies for the 18‐node model. H) The red curve represents the curve of the average embedding error as λ varies, while the gray curves represent the curve of all embedding errors as λ varies for the 18‐node model.

To illustrate how stPCA effectively characterizes high‐dimensional time series, two network models were employed:^[^ ^47^ ^]^ a sixteen‐node/variable system with a Fold bifurcation (**Figure** **3**A) and an eighteen‐node/variable system with a Hopf bifurcation (Figure 3E). More details of these two networks are presented in Figures S8, S9, and in Note S5 (Supporting Information). From these systems, two datasets were generated by varying parameter τ from 1 to 310 and from 1 to 190, respectively (Tables S2 and S3, Supporting Information). The critical transition points were set at τ = 210 (Hopf bifurcation) and τ = 100 (fold bifurcation). To investigate different states with the varying parameters of a dynamical system, a sliding‐window scheme was implemented to divide the entire process into smaller windows. For each window, the dynamics of the high‐dimensional time series are reduced to that of a latent variable *z* from stPCA (Figure 3B,F). Based on the CSD or dynamic network biomarker (DNB),^[^ ^44^ ^]^ the abrupt increase in the standard deviation (SD) of **z** = (*z* ^1^,*z* ^2^,⋅⋅⋅, *z^m^*,*z* ^*m* + 1^, ⋅⋅⋅, *z* ^*m* + *L* − 1^) (or the fluctuations of the “*z*” dynamics in a sliding window) indicates the upcoming critical transition (Figure 3C,G). The details are shown in the Materials and Methods section.

In addition, when the regularization parameter λ varies from 0.78 to 0.98, stPCA can effectively detect the imminent critical transitions of systems with fold bifurcation (Figure 3C) and Hopf bifurcation (Figure 3G) through the one‐dimensional dynamics of *z*. However, the embedding error $E_{embedding}=∥Z-\hat{Z}{∥}_{F}$ increases accordingly (Figure 3D,H) with decreasing λ. To meet the delay embedding condition so that $\hat{Z}$ can reconstruct the original high‐dimensional dynamics, it is necessary to keep the embedding error below 2, which suggests that λ typically ranges from 0.9 to 1.

### 2.4 ICU Decision‐Making: Real‐World Application of stPCA on MIMIC Datasets

The MIMIC‐III database^[^ ^49^ ^]^ is a widely used resource for studying critical care patients.^[^ ^57^, ^58^ ^]^ It comprises deidentified health‐related data associated with patients who stayed in the critical care units of the Beth Israel Deaconess Medical Center between 2001 and 2012 and contains comprehensive clinical information collected from the ICU stays. The percentage of patients who experienced death within a 5‐year period was 2.3%, and unplanned 30‐day readmissions were 12.9%; therefore, making accurate judgments about whether a patient is ready for discharge from the ICU is of utmost importance in optimizing life‐saving measures with the constraints of available medical resources. By analyzing these heterogeneous high‐dimensional noisy data, we aimed to gain insights into the complex nature of critical care conditions and contribute to improving patient management and decision‐making processes.

**Figure 4:**
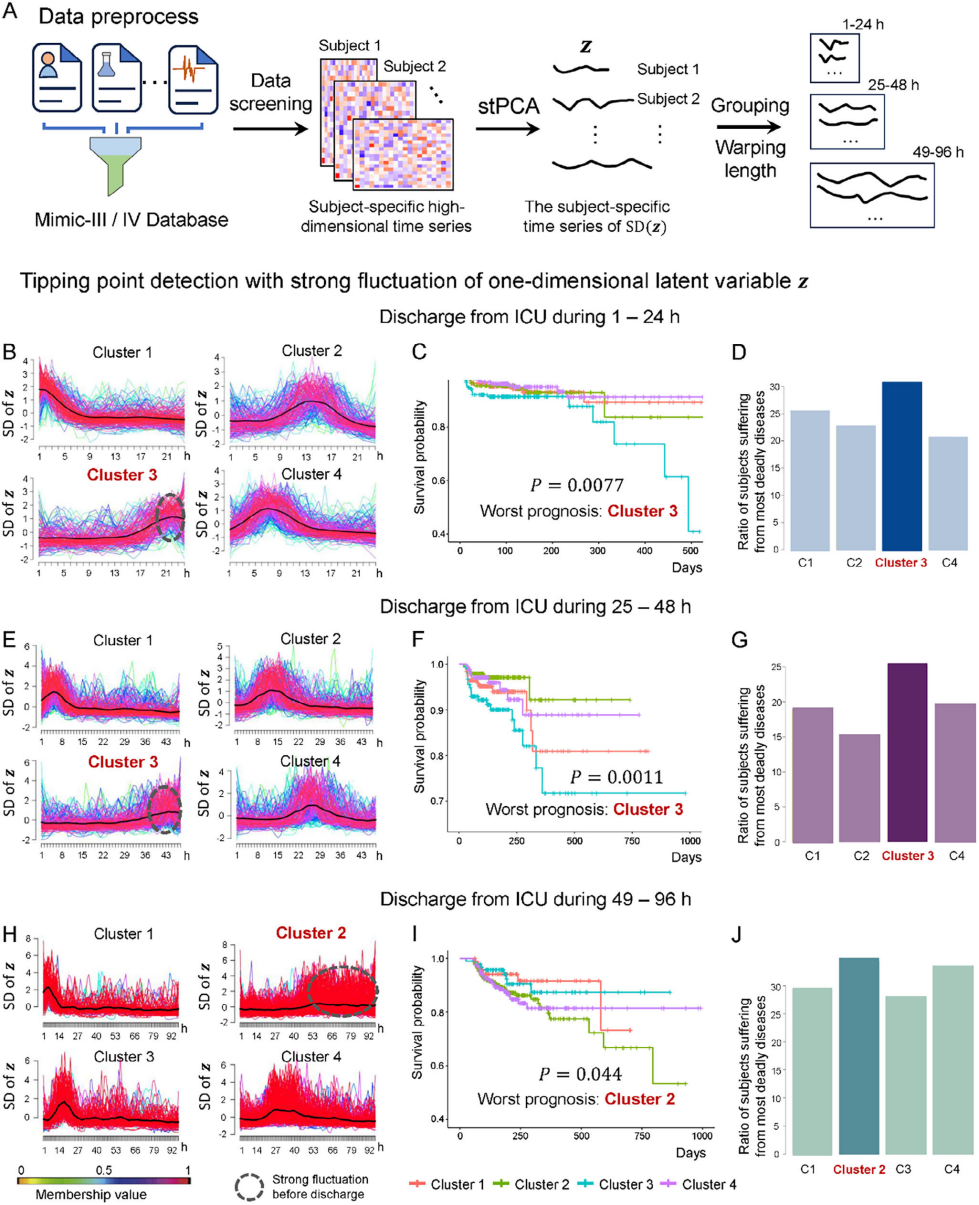
Performance of stPCA applied on the MIMIC‐III dataset with three groups on patients’ ICU stay. A) Patient admission data were extracted from the ICU PDMS. According to the screening criteria, we obtained 2908 patient‐specific matrices that represent tested disease indicators at different hours. The patients were divided into three groups, with B–D) representing patients who stayed in the ICU for 1–24 h, E–G) representing patients who stayed in the ICU for 25–48 h, and H–J) representing patients who stayed in the ICU for 49–96 h. For each group, the high‐dimensional disease indicators of each patient were dimensionally reduced to a single variable by stPCA. Then, a DTW scheme was adopted to align these time series so that they have the same length. B) Each colored curve corresponds to the SD of the one‐dimensional variable for each patient, and the black curve represents the average SD. Then, time series clustering was performed on these curves, resulting in four clusters. Notably, Cluster 3 exhibits strong fluctuations in the later stages. C) Survival analysis results for the four clusters with an ICU duration of 1–24 h. D) Proportion of patients in the four clusters with an ICU duration of 1–24 h who experienced the highest disease mortality rate. E) Time series clustering results for patients with an ICU duration of 25–48 h, with Cluster 3 exhibiting strong fluctuations in the later stages. F) Survival analysis results for the four clusters with ICU durations of 25–48 h. G) Proportion of patients in the four clusters with ICU durations of 25–48 h who experienced diseases with the highest mortality rates. H) Time series clustering results for patients with ICU durations of 49–96 h, with Cluster 2 exhibiting strong fluctuations in the later stages. I) Survival analysis results for the four clusters with ICU durations of 49–96 h. J) Proportion of patients in the four clusters with ICU durations of 49–96 h who experienced diseases with the highest mortality rates.

In this study, we first applied stPCA to a subset of the MIMIC‐III database. We randomly selected 10 000 patients from this database. After a rigorous preprocessing procedure shown in Figure S14 (Supporting Information), which involved data cleaning and quality control measures, a final dataset comprising 2908 patients was obtained (**Figure** **4**A). The data of each patient were represented by a heterogeneous high‐dimensional time series, i.e., a matrix consisting of at least 10 time points indicating the number of hours since his/her admission and containing no less than five diagnosis‐related indicators. Additional details of the preprocessing process are provided in Figure S11 (Supporting Information).

For each patient, the high‐dimensional time series was reduced to one variable *z* by stPCA with the sliding‐window scheme, and then the fluctuation of *z*, *Fl^z^*, was calculated;, i.e., $Fl_{\left(k\right)}^{z}=\;\mathrm{SD}\left(z_{\left(k\right)}^{1},z_{\left(k\right)}^{2},\text{…},z_{\left(k\right)}^{m},z_{\left(k\right)}^{m+1},\text{…},z_{\left(k\right)}^{m+L-1}\right)$ was calculated for the *k*th sliding window (Figure S12, Supporting Information). Based on the individual variations in ICU stay duration, we classified the curves of *Fl^z^* into four groups: Group 1 (ICU stay duration 1–24 h), Group 2 (ICU stay duration 25–48 h), Group 3 (ICU stay duration 49–96 h), and Group 4 (ICU stay duration exceeding 4 days). To explore underlying dynamic patterns among patients in each group, we first used the dynamic time warping (DTW) algorithm^[^ ^59^, ^60^ ^]^ to align the curves of *Fl^z^* so that they have a consistent length. The details of the DTW algorithm are presented in Note S8 (Supporting Information).

Then, the SD curves were clustered^[^ ^60^, ^61^ ^]^ within each group based on the aligned time series. Four clusters were obtained within each group (Figure 4B,E,H). Notably, there is a common pattern among Cluster 3 of Group 1 (Figure 4C), Cluster 3 of Group 2 (Figure 4F), and Cluster 2 of Group 3 (Figure 4I), all of which exhibit relatively strong fluctuations in *z* in the later stages, correlating with the poorest recorded prognosis within the corresponding cluster. In addition, there was a significant difference (pvalue < 0.05) in the prognoses of patients among different clusters in each group. Furthermore, by examining the proportion of patients within each cluster who suffered from diseases/diagnoses with the highest mortality rates (Figure 4D,G,J), it was found that in these clusters with poor prognoses, the proportions of patients afflicted by diagnoses with high mortality rates were also higher. Based on these observations, we speculate that for an individual patient, the significant fluctuations of *z* from stPCA may indicate a critical condition, warranting close monitoring and ongoing treatment until the *Fl* ^z^ index stabilizes.

Furthermore, on the basis of *Fl^z^*, i.e., the fluctuation of the latent variable *z*, and 2–5 diagnosis‐specific indicators, we proposed a new scheme to determine whether a patient should be discharged from the ICU at time point *t*, i.e., ICU decision‐making, as follows:

In recent hours, {t−wl+1,…,t−1,t}, where *wl* represents the window length, if the average *Fl* ^z^ for a patient is significantly lower than the highest value observed since admission, it indicates a relatively stable condition after treatment. An index idx_*z*_(*t*) of *z* is defined as follows:

(9) $$\mathrm{id}x_{z}\left(t\right)=\max\left(\mathrm{mean}\left(S_{\mathrm{past}\left(j\right)}\right)\right)\;/\;\mathrm{mean}\left(S_{\mathrm{recent}}\right)$$

where vector **S**_past(*j*)_ represents a vector of standard deviations of *z* during a past period (time window *j*);, i.e., $S_{\mathrm{past}(j)}=\left(Fl_{\left(j-wl+1\right)}^{z},\text{…},Fl_{\left(j-1\right)}^{z},Fl_{\left(j\right)}^{z}\right)$, with j=wl,wl+1,…,t−1. In addition, vector **S**_recent_ represents the standard deviations of *z* over the most recent *wl* time points, i.e., $S_{\mathrm{recent}}=\left(Fl_{\left(t-wl+1\right)}^{z},\text{…},\;Fl_{\left(t-1\right)}^{z},Fl_{\left(t\right)}^{z}\right)$.

During the recent period, 2–5 diagnosis‐specific items $x_{i}^{t}$ that are most closely associated should fall within the normal range (see Table S4, Supporting Information). This can be evaluated using the following condition: $\mathrm{itmFlg}\;\left(t\right)=\sum_{i=1}^{K}\#\left(x_{i}^{t}\right)$ with item flags

(10) $$\#\left(x_{i}^{t}\right)=\left\{\begin{array}{cc} 1, & Lb_{i}\leq x_{i}^{t}\leq Ub_{i},\\ 0, & \mathrm{otherwise}, \end{array}\right.$$

where *K* is the number of selected items, *Lb_i_* and *Ub_i_* represent the lower and upper bounds of the normal range for $x_{i}^{t}$, respectively.

Then, the decision for ICU discharge at time point *t* is described as follows:

(11) $$\mathrm{Decision}\;\left(t\right)=\left\{\begin{array}{cc} 1, & \mathrm{id}\;x_{z}\left(t\right)\geq FC\;\mathrm{and}\;\mathrm{itmFlg}\left(t\right)=K\\ 0, & \mathrm{otherwise}, \end{array}\right.$$

where *FC* represents the fold change threshold. If Decision (*t*) = 1, i.e., a positive decision at time *t*, then the patient's condition is stable at time point *t*, thereby indicating suitability for ICU discharge. Otherwise, if Decision (*t*) = 0, i.e., a negative decision at time *t*, then the patient should stay in the ICU for further treatment and monitoring. An illustration of this discharge scheme is presented in Figure S13 (Supporting Information).

A set of standardized metrics are employed for evaluating the performance of the ICU discharge decision for a specific method as follows. When Decision (*t*) = 1, if the patient is discharged during a period of 5 h after *t* or all the relevant items thereafter are within the normal range, then this decision is considered a true positive (TP); otherwise, it is a false‐positive (FP). When Decision (*t*) = 0, if the patient is currently receiving treatment in the ICU, then this decision is considered a true negative (TN); otherwise, this decision is considered a false negative (FN). The F1‐score, accuracy (ACC), true positive rate (TPR)/recall, false positive rate (FPR), and precision metrics are calculated based on TP, FP, TN, and FN. The detailed descriptions and calculation formulas for these metrics are provided in Note S6 (Supporting Information).

**Figure 5:**
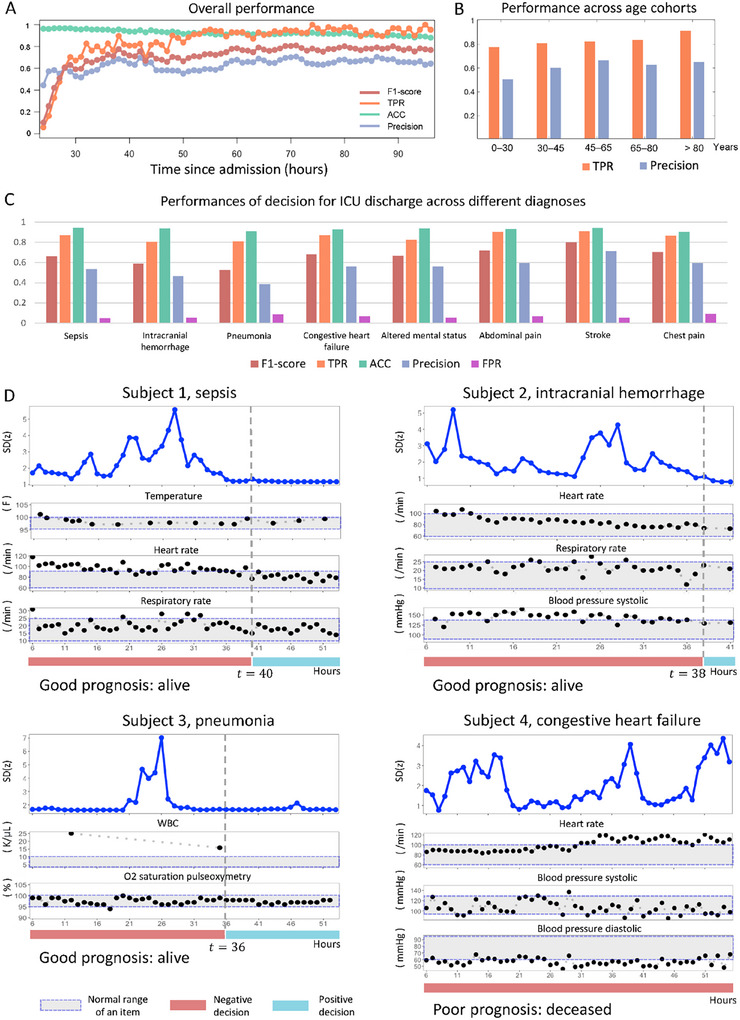
Performance of the decision for ICU discharge. A) For all samples across diagnoses of 2908 patients, the performance of stPCA‐based discharge decision was assessed for each hour since admission. B) TPR/Recall and Precision across five age cohorts are presented. C) For the top eight diagnoses with the highest mortality rate, we evaluate the performance of our discharge decision metrics based on the stPCA algorithm. D) We present four typical cases (patients suffering from sepsis, intracranial hemorrhage, pneumonia, and congestive heart failure). Combining the dimensionality reduction results of stPCA and 2–5 diagnosis‐related indicators, we can make decisions on whether patients should be discharged from the ICU. The red interval indicates that the patient should continue receiving ICU treatment or observation, while the blue interval suggests that the patient's condition is relatively stable and that they can be discharged from the ICU.

Based on the aforementioned scheme, the overall performance of stPCA‐based decision‐making was assessed over the course of time since admission (**Figure** **5**A). The precision and F1‐score are ≈0.6 and 0.7, while TPR/recall and ACC metrics are all greater than 0.8 when *t* ≥ 30 h. When *t* < 30 h, the slightly lower F1‐score and TPR are due to the limited number of discharged patients. Then, the performances across five age cohorts are demonstrated in Figure 5B. Moreover, Figure 5C presents the decision performance of stPCA for the top‐8 diagnoses with the highest mortality rates: sepsis, intracranial hemorrhage, pneumonia, congestive heart failure, altered mental status, abdominal pain, stroke, and chest pain. Across various metrics, our decision scheme based on stPCA consistently demonstrates favorable results. Figure 5D illustrates the assessment of ICU discharge for four subjects diagnosed with sepsis, intracranial hemorrhage, pneumonia, and congestive heart failure. When *t* ≥ 40 h, 38 h, and 36 h for Subjects 1, 2, and 3, respectively, we determined that Decision (*t*) = 1; thus, Subjects 1–3 met the criteria for ICU discharge after treatment, which is consistent with their positive prognoses, as recorded. Conversely, Subject 4 exhibited inadequate treatment response and experienced a deteriorating state over time, which is consistent with the recorded negative prognosis.

### 2.5 Performance Comparisons of Dimensionality Reduction Methods on the MIMIC‐III and MIMIC‐IV Datasets

To further evaluate the performance of the stPCA algorithm in ICU decision‐making, stPCA is compared with seven commonly used dimensionality reduction or data representation methods including (1) PCA,^[^ ^9^ ^]^ (2) LLE,^[^ ^7^ ^]^ (3) t‐SNE,^[^ ^4^ ^]^ (4) MDS,^[^ ^6^ ^]^ (5) ISOMAP,^[^ ^8^ ^]^ (6) AE,^[^ ^17^ ^]^ (7) variational autoencoder (VAE),^[^ ^62^ ^]^ (8) MARBLE,^[^ ^23^ ^]^ (9) CANDyMan,^[^ ^22^ ^]^ (10) HAVOK,^[^ ^26^ ^]^ which can potentially be applied to the decision‐making process of ICU patient discharge. The ten methods for comparison are listed below, with hyperparameters mainly adopted from the original references. Further details are presented in Note S9 (Supporting Information). On the MIMIC‐III data, the results for all samples across diagnoses of 2908 patients and the top‐8 high‐mortality diagnoses are illustrated in **Table** **1**. Clearly, stPCA consistently achieves the best performance among all methods. The comparisons of time complexity between DNN‐based representation learning methods and stPCA are provided in Note S10 (Supporting Information).

**Table 1:** Comparison of discharge‐decision performances on the MIMIC‐III dataset for stPCA and 10 other dimensionality reduction methods.

Method	Diagnosis Metrics	All samples	Sepsis	Intracranial hemorrhage	Pneumonia	Congestive heart failure	Altered mental status	Abdominal pain	Stroke	Chest pain
stPCA	Recall	0.835	0.869	0.803	0.810	0.869	0.825	0.902	0.911	0.863
	Precision	0.639	0.534	0.467	0.386	0.560	0.559	0.593	0.713	0.595
	F1‐score	0.724	0.662	0.591	0.523	0.681	0.666	0.716	0.800	0.704
PCA	Recall	0.625	0.798	0.538	0.486	0.834	0.569	0.211	0.766	0.604
	Precision	0.507	0.685	0.318	0.379	0.569	0.541	0.154	0.837	0.733
	F1‐score	0.560	0.737	0.400	0.426	0.676	0.555	0.178	0.800	0.662
LLE	Recall	0.496	0.000	0.308	0.333	0.500	0.547	0.063	0.000	0.178
	Precision	0.497	0.000	0.500	0.253	0.914	0.744	0.067	NaN	0.099
	F1‐score	0.496	NaN	0.381	0.288	0.646	0.630	0.065	NaN	0.127
t‐SNE	Recall	0.556	0.429	0.750	0.760	0.538	0.549	0.714	0.519	0.593
	Precision	0.476	0.178	0.468	0.442	0.574	0.196	0.507	0.400	1.000
	F1‐score	0.513	0.252	0.576	0.559	0.555	0.289	0.593	0.452	0.745
MDS	Recall	0.196	0.000	0.000	0.000	0.267	0.000	0.000	0.000	0.116
	Precision	0.471	0.000	NaN ^a)^	0.000	1.000	NaN	NaN	NaN	0.385
	F1‐score	0.277	NaN	NaN	NaN	0.421	NaN	NaN	NaN	0.178
ISOMAP	Recall	0.257	0.257	0.182	0.494	0.396	0.468	0.000	0.400	0.269
	Precision	0.495	0.205	0.500	0.534	0.875	0.400	0.000	0.615	0.359
	F1‐score	0.338	0.228	0.267	0.513	0.545	0.431	NaN	0.485	0.308
AE	Recall	0.486	0.667	0.441	0.587	0.568	0.438	0.375	0.820	0.357
	Precision	0.465	0.464	0.312	0.514	0.618	0.286	0.231	0.696	0.357
	F1‐score	0.475	0.547	0.365	0.548	0.592	0.346	0.286	0.753	0.357
VAE	Recall	0.670	0.767	0.561	0.598	0.819	0.554	0.516	0.772	0.576
	Precision	0.587	0.439	0.535	0.359	0.659	0.240	0.356	0.379	0.690
	F1‐score	0.626	0.558	0.548	0.449	0.730	0.335	0.421	0.508	0.628
MARBLE	Recall	0.568	0.677	0.502	0.612	0.515	0.527	0.611	0.692	0.632
	Precision	0.533	0.624	0.445	0.480	0.562	0.500	0.544	0.489	0.571
	F1‐score	0.550	0.649	0.472	0.538	0.537	0.513	0.576	0.573	0.600
CANDyMan	Recall	0.622	0.714	0.550	0.562	0.681	0.610	0.598	0.582	0.628
	Precision	0.496	0.563	0.425	0.507	0.542	0.420	0.520	0.423	0.418
	F1‐score	0.552	0.630	0.479	0.533	0.604	0.497	0.556	0.490	0.502
HAVOK	Recall	0.643	0.574	0.704	0.514	0.572	0.642	0.620	0.657	0.650
	Precision	0.511	0.536	0.581	0.445	0.539	0.478	0.533	0.504	0.498
	F1‐score	0.569	0.554	0.637	0.477	0.555	0.548	0.573	0.570	0.564

^a)^ NaN means there is no such event.

Additionally, we extended the application of our algorithm to an independent database called MIMIC‐IV, which comprises medical records corresponding to patients admitted to an ICU or the emergency department between 2008 and 2019.^[^ ^50^ ^]^ Similarly, a cohort of 10000 patients was randomly selected from this extensive dataset. After subjecting the data to a rigorous preprocessing procedure similar to that for MIMIC‐III (Figure 4A), we curated a focused sub‐dataset consisting of 625 patients, based on which the ICU decision‐making approach from stPCA was compared with those from seven other data representation methods, which is in line with the above evaluation protocol. The performance metrics for all diagnoses of 625 subjects, as well as those pertaining to the top‐5 most prevalent diagnoses, are shown in **Table** **2**. In a general overview, it is noted that our discharge strategy from stPCA consistently outperforms those from other methods. The observation from applications on both the independent MIMIC‐III and MIMIC‐IV datasets highlights the robustness and effectiveness of the stPCA approach.

**Table 2:** Comparison of discharge‐decision performances on the MIMIC‐IV dataset for stPCA and 10 other dimensionality reduction methods.

Method	Diagnosis Metrics	All samples	Acute posthemorrhagic anemia	Pure hypercholesterolemia	Acidosis	Thrombocytopenia	Cardiogenic shock
stPCA	Recall	0.886	0.913	0.966	0.973	0.933	0.988
	Precision	0.676	0.668	0.824	0.636	0.617	0.837
	F1‐score	0.767	0.772	0.889	0.769	0.743	0.906
PCA	Recall	0.553	0.491	0.9	0.804	0.369	0.952
	Precision	0.684	0.358	0.006	0.643	0.346	0.900
	F1‐score	0.611	0.414	0.885	0.714	0.358	0.925
LLE	Recall	0.091	0.090	0	0.350	0.288	0.667
	Precision	0.493	0.818	NaN ^a)^	0.667	0.826	0.780
	F1‐score	0.154	0.167	NaN	0.459	0.427	0.719
t‐SNE	Recall	0.671	0.743	0.923	0.76	0.586	0.927
	Precision	0.714	0.699	0.774	0.31	0.288	0.692
	F1‐score	0.692	0.720	0.842	0.441	0.386	0.793
MDS	Recall	0.317	0.340	0	0.631	0.229	0.842
	Precision	0.631	0.776	0	0.477	0.438	1
	F1‐score	0.422	0.474	NaN	0.543	0.301	0.914
ISOMAP	Recall	0.766	0.818	0.833	0.875	0.818	0.907
	Precision	0.783	0.886	1	0.509	0.706	0.834
	F1‐score	0.774	0.851	0.909	0.644	0.758	0.869
AE	Recall	0.284	0.446	0.651	0.627	0.254	0.846
	Precision	0.709	0.631	0.757	0.646	0.340	0.733
	F1‐score	0.405	0.522	0.700	0.636	0.291	0.786
VAE	Recall	0.686	0.554	0.855	0.785	0.365	0.851
	Precision	0.634	0.663	0.776	0.646	0.265	0.776
	F1‐score	0.659	0.603	0.813	0.709	0.307	0.814
MARBLE	Recall	0.514	0.591	0.842	0.901	0.675	0.927
	Precision	0.493	0.583	0.772	0.782	0.721	0.658
	F1‐score	0.503	0.587	0.805	0.837	0.697	0.770
CANDyMan	Recall	0.691	0.455	0.872	0.575	0.487	0.884
	Precision	0.489	0.470	0.711	0.387	0.392	0.582
	F1‐score	0.573	0.462	0.783	0.463	0.434	0.702
HAVOK	Recall	0.727	0.924	0.906	0.432	0.465	0.917
	Precision	0.604	0.586	0.712	0.392	0.371	0.776
	F1‐score	0.660	0.717	0.797	0.411	0.413	0.841

^a)^ NaN means there is no such event.

### 2.6 Identifying the Tipping Point for Single‐Cell Embryonic Development

**Figure 6:**
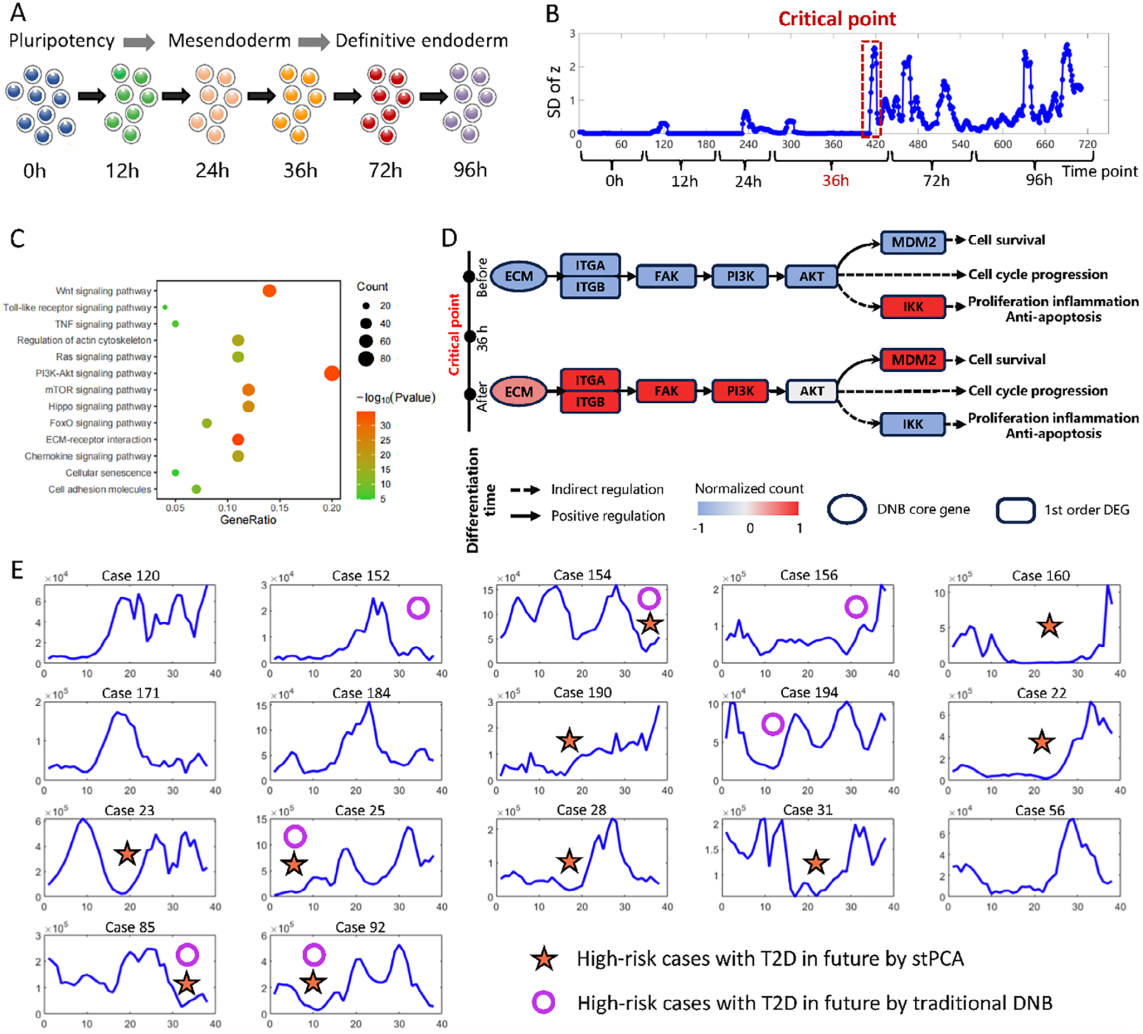
Detecting the tipping points during embryonic development and analyzing T2D risk by stPCA. A) The biological process of cell differentiation from a pluripotent state to mesendoderm and, finally, to definitive endoderm (DE) cells. B) The SD curve of the latent variable *z* with the sliding‐window scheme. It is shown that there was a sudden increase at 36 h during the differentiation. C) The pathway enrichment analysis for the stPCA‐signaling genes/DNB. D) The underlying molecular mechanism revealed by the functional analysis of stPCA‐signaling genes and their first‐order neighboring differentially expressed genes (DEGs). E) The SD of *z* for 17 cases confirmed T2D subsequently. The orange star presents a case with a high risk of developing T2D by stPCA, and the blue circle denotes a case with a high risk of developing T2D by traditional DNB.

To illustrate how stPCA detects the signal of cell differentiation, the proposed method was applied to an scRNA‐seq dataset of embryonic development (**Figure** **6**A)^[^ ^51^ ^]^ We selected the top 200 highly variable genes, and then reordered 758 single cells along the pseudotime.^[^ ^63^ ^]^ Subsequently, the SD of *z* was calculated by stPCA with the sliding window scheme. From Figure 6B, it is seen that there is an abrupt increase in the SD curve at 36 h, which signals the cell fate transition of differentiation into definitive endoderm. The identified critical transition is validated by the experimental observation, that is, such signal is notably earlier than the typical emergence of DE cells around days 4 or 5 in established human pluripotent stem cell protocols.^[^ ^51^ ^]^

To investigate the molecular regulation mechanism underlying cell differentiation during embryonic development, biological functional analyses such as signaling pathway enrichment analysis and gene annotation were performed to reveal the potential biological functions of 50 stPCA‐signaling/DNB genes found at 36 h and their first‐order differentially expressed genes (DEGs). The enrichment results showed that these genes were mainly enriched in the Wnt signaling pathway, PI3K‐Akt signaling pathway, mTOR signaling pathway, and other differentiation‐related signaling pathways (Figure 6C). The results also show that the synergy of the stPCA‐signaling genes and their 1st‐order neighboring DEGs regulate cell cycle progression around the critical point through the PI3K‐Akt pathway (Figure 6D).

Actually, the identified critical transition as well as the enriched PI3K signaling pathway were supported by recent studies,^[^ ^64^ ^]^ in which the intricate interplay between the PI3K signaling pathway, encompassing PI3K and its downstream molecule Akt/PKB, and the preservation of self‐renewal and multilineage differentiation potential in embryonic stem cells (ES cells) was found essential for cell proliferation and differentiation. This pathway has also exhibited significant involvement in orchestrating cell proliferation and differentiation across a spectrum of cell types.^[^ ^65^ ^]^ Figure 6D elucidates potential regulatory mechanisms for the functional analysis of stPCA‐signaling genes and their immediate neighbors. Notably, growth factors such as extracellular matrix (ECM) components COL4A1 and SPP1 have emerged as influential upstream regulatory factors propelling cells through critical developmental transitions during the process of cell differentiation. These factors exhibit upregulation after critical transition, potentially steering downstream gene expressions. Furthermore, within the PI3K/Akt pathway, a responsive signaling cascade to stPCA‐signaling genes has been discerned, exerting a pivotal influence on the regulation of cell proliferation. Upregulation of upstream stPCA‐signaling genes COL4A1 and SPP1 initiates a cascade effect, culminating in the upregulation of downstream neighboring genes ITGB9, ITGB8, and PTK2. This, in turn, activates the AKT protein, leading to increased MDM2 gene expression and thereby fostering cell proliferation and differentiation. As a key stPCA‐signaling gene, COL4A1 plays a critical role in cell adhesion, migration, proliferation, and differentiation through its interaction with integrin receptors. In summary, the induced downstream signaling reactions of stPCA‐signaling genes actively engage in the intricate regulation of cell proliferation and differentiation. The sustained expression of stPCA‐signaling genes in these pathways, both before and after critical transitions, highlights the pivotal role of identified critical transitions in guiding embryonic stem cell development.

#### 2.6.1 Predicting the T2D Transition Risk Based on Continuous Glucose Monitoring Data

We applied stPCA to a continuous glucose monitoring (CGM) dataset^[^ ^52^ ^]^ to analyze the risk of T2D. There were 208 patients selected from the outpatient clinic of hypertension and vascular risk of the University Hospital of Móstoles, in Madrid, from January 2012 to May 2015. A CGM record was obtained for 48 h with sampling every 5 min. Patients were then followed every 6 months until the diagnosis of T2D or the end of the study. There were 17 confirmed cases of T2D, with a median time to diagnosis of 33.8 months after the sampling time point. For each subject, the CGM time‐series data were segmented according to the following rules:

1. Segmentation was performed based on the three main meals: breakfast, lunch, and dinner.
2. Meal times were determined using local maxima in the CGM data. Breakfast: within the time range 6:00–9:00, identified by the local maximum T1, corresponding to the period [T1‐3 h, T1+1 h]. Lunch: within the time range 11:00–14:00, identified by the local maximum T2, corresponding to the period [T2‐3 h, T2+1 h]. Dinner: within the time range 17:00–21:00, identified by the local maximum T3, corresponding to the period [T3‐3 h, T3+1 h].
3. Additionally, two segments were added for the early morning (2:00–6:00) and fasting (21:00–1:00 of the next day) periods on the first day. Consequently, each subject's CGM time‐series was divided into eight segments, each containing CGM data measured over a 4‐h interval, representing a 48‐dimensional vector.

In this process, an 8‐dimensional time series was generated for each case. Subsequently, we applied stPCA to this high‐dimensional time series and computed the SD of the latent variable *z*. A substantial fluctuation (SD exceeding the average value) in *z* indicates an elevated risk of transitioning from a healthy state to T2D for one case. 17 cases confirmed T2D were demonstrated in Figure 6E. Notably, stPCA identified 10 cases with a high risk of developing T2D in the future, surpassing predictions made by the traditional DNB approach. Therefore, stPCA is effective in providing early warnings for T2D risk based on CGM data.

## 3 Discussion

The stPCA framework is proposed to represent the dynamical features of observed high‐dimensional time‐series data by only a single latent variable through a spatiotemporal transformation that converts high‐dimensional spatial information into low‐dimensional temporal information. This representative variable is acquired in an analytical and interpretable way by solving a unique characteristic equation and theoretically can preserve the dynamical property of the original high‐dimensional space. In contrast to change‐point detection, stPCA aims to predict the change point before the change through critical collective fluctuations based on CSD theory, requiring only samples before the tipping point. However, change‐point detection methods, such as GSL^[^ ^66^ ^]^ and stdCPD^[^ ^67^ ^]^ generally require samples from both before and after the tipping point. In addition, the comparisons between the analytical solution from stPCA and an iterative numerical optimization algorithms such as sequential quadratic programming^[^ ^40^ ^]^ for a coupled Lorenz system and a multiple‐node network show that the former yields a much more stable and accurate result (Figures S6 and S10, Supporting Information). Compared with DNN‐based representation learning method,^[^ ^20^, ^21^, ^22^, ^23^ ^]^ stPCA achieves high efficiency with the time complexity of *O*(*nL*).

Similar to the Koopman operator which can theoretically linearize high‐dimensional nonlinear systems into an infinite linear space (without approximation) through spectral decomposition of infinite eigenfunctions, stPCA solves an optimization problem to represent high‐dimensional systems with only one singular latent variable by linear embeddings (with approximation) in an analytical way with one eigenvalue/eigenvector. However, eigenfunctions of the Koopman operator K need to be sought (without a general method).^[^ ^24^, ^26^, ^28^ ^]^ Representations have proven difficult to obtain and are often intractably complex or uninterpretable. DNN and DMD can be utilized for approximating the Koopman operator, but they face other challenges related to accuracy, training sample size, and time cost. As for stPCA, eigenvectors of matrix *H*(*X*) can be relatively easy to obtain (as shown in the subsection of “The stPCA algorithm”), and the latent representation variable is derived from one dominant eigenvalue and its eigenvector. This property makes stPCA effective even for small sample sizes or short time‐series data, as well as analytically tractable and computationally fast even for large datasets.

The applications of stPCA to both theoretical models and real‐world datasets demonstrate its effectiveness and robustness in temporal data representation, tipping‐point detection, and risk analysis of complex systems. First, implementing stPCA on coupled Lorenz models provided evidence that this method is effective in achieving efficient dimensionality reduction to an ultralow‐dimensional space and exhibits resistance to noise. In addition, for ultrashort time‐series cases, the extra spatial information can be transformed into temporal information of a latent variable, thereby further enhancing the robustness of stPCA to noise interference. This is demonstrated in Figure 2, illustrating stPCA's ability to transform spatiotemporal information. Second, through the prognosis analysis of various cohort groups for ICU patients, the significant fluctuations of the latent single variable 𝑧 for each individual patient may indicate the critical/tipping condition. Thus, a scheme was proposed by combining the SD of *z* and 2–5 diagnosis‐related items to determine whether a patient should be discharged from the ICU at a specific time point, as demonstrated in the applications to MIMIC‐III and MIMIC‐IV datasets. Third, we compared this stPCA‐based scheme with seven other dimensionality reduction methods, revealing that stPCA consistently outperforms its counterparts.

From the above analyses and applications, it is seen that stPCA possesses several noteworthy advantages: i) stPCA excels in achieving ultralow‐dimensionality reduction for dynamical systems without distortion. Its innovative use of a delay embedding strategy eliminates the need for truncation (approximation), a fundamental requirement in other dimensionality reduction methods. ii) The transformation matrix *W* and the single variable *z* are derived through an analytical solution by solving a unique characteristic equation, which makes stPCA tractable and allows fast computation scalable to large datasets. iii) stPCA efficiently represents the dynamics or temporal features of high‐dimensional time‐series data using only a single latent variable. This one‐dimensional variable is roughly considered to represent the center manifold near the tipping point, enabling effective detection of the impending critical transitions in dynamical systems with codim‐1 local bifurcation by examining the SD of this single variable.

There are still challenges and promising directions that motivate future work. First, there are still several limitations associated with the linear embedding *Z* = *WX*. Similar to PCA, which is mainly used for cross‐sectional samples with the truncation of principal components as the approximation, stPCA is used for time‐series samples but without truncation. Both methods adopt linear embedding due to their simple and explainable form, making them widely applicable in various fields. Such a linear embedding may not accurately characterize the nonlinear dynamics or may make inaccurate predictions of nonlinear dynamics. However, rather than to predict the overall dynamics, our method aims to identify critical transition dynamics which actually explores such inaccuracy (e.g., strong fluctuations). Specifically, when the prediction of a system by stPCA at a certain period or state, i.e., near a tipping point, becomes inaccurate due to its high nonlinearity or strong fluctuations during this period or state, the linear‐embedding features in a one‐dimensional space can be employed to detect this tipping point based on the CSD theory or our critical collective fluctuation principle. In other words, the detection of the tipping point can be actually quantified based on the inaccuracy (or fluctuation) of prediction.

Another drawback about stPCA is its sensitivity to the scaling of certain coordinates, which may affect the quality or stability of the results. Therefore, normalization of the original data is an essential step in the stPCA procedure. Nonetheless, despite this scaling concern, stPCA remains effective in identifying the tipping point using one single latent variable, in contrast to PCA which necessitates multiple representation variables. Actually, to mitigate this issue, we can also transform the information not directly from the original data or space, but from a feature space converted with a deep learning framework in our future work. Additionally, some hyperparameters for ICU decision‐making are empirically determined based on our experimental setup. Future effort will also focus on refining hyperparameter tuning, possibly through the integration of deep learning techniques or pre‐trained models, to obtain more universally applicable and reliable values.

Despite these limitations, stPCA is proficient in detecting the critical transitions across diverse fields when compared with some previous works in predicting critical behaviors.^[^ ^68^ ^]^ It provides a novel approach to dimensionality reduction for high‐dimensional time‐series data of nonlinear dynamical systems, characterized by computational efficiency, dynamics‐preserving, accuracy, and robustness, thus holding significant promise for real‐world applications.

## 4 Experimental Section

### Spatial‐Temporal Information Transformation

The steady state or attractor was generally constrained in a low‐dimensional space for a high‐dimensional dissipative system, which holds for most real‐world complex systems. By exploring such a low‐dimensional feature, spatial‐temporal information (STI) transformations have been theoretically derived from delay‐embedding theory^[^ ^35^, ^36^ ^]^ and could be used to transform the spatial information of high‐dimensional data to the temporal information of any target variable. Specifically, for a general discrete‐time dissipative system, the dynamics could be defined as

(12) $$X^{t+1}=\phi \left(X^{t}\right)$$

where $\phi :\;R^{n}\to R^{n}$ was a nonlinear map, and its variables were defined in the *n*‐dimensional state space 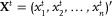 at a time point *t*, where symbol “ ′ ” was the transpose of a vector, and any time interval between two consecutive time points was equal. After a sufficiently long time, all states converge into a compact manifold V. Takens’ embedding theorem was stated as follows.^[^ ^69^ ^]^

If $V\subseteq R^{n}$ was an attractor with the box‐counting dimension *d*, for a smooth diffeomorphism ϕ:V→V and a smooth function h:V→R, there was a generic property that the mapping $\Phi_{\phi ,h}:V\to R^{L}$ was an embedding when *L* > 2*d*; that was,

(13) $$\Phi_{\phi ,h}\;\left(X\right)={\left(h\left(X\right),h\circ \;\phi \left(X\right),\text{…},\;h\circ \phi^{L-1}\left(X\right)\right)}^{\prime }$$

where symbol “○” was the function composition operation. In particular, letting *X* = **X** ^*t*^, Φ_ϕ,*h*_ = Φ and *h* (**X** ^*t*^) = *z^t^*, where $z^{t}\in R$, the STI equations were expressed in Equation (14):
(14)

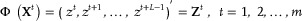

where $\Phi :R^{n}\to R^{L}$ was a differentiable function and *m* was the length of the original high‐dimensional time series *X* (see Notes S1 and S2 for details, Supporting Information). Clearly, **X** ^*t*^ in Equation (14) was the spatial information of *n* variables, while **Z** ^*t*^ was the temporal information of the target variable. In other words, **Z** ^*t*^ can be viewed as an observable of Equation (12) according to the generalized delay embedding theorem, and intuitively, Equation (14) transforms the original spatial information **X** ^*t*^ into the encoded temporal information **Z** ^*t*^. Thus, the dynamics of the original system **X** ^*t*^ can be topologically reconstructed from a 1D delay embedding **Z** ^*t*^, which was employed to identify the critical state transition later.

In this study the aim was to identify the critical state transition, Equation (14) was further linearized as

(15) Z=WX

where *X*, *W*, and *Z* were defined as

(16) $$X={\left(\begin{array}{cccc} x_{1}^{1} & \;x_{1}^{2} & \;\text{…} & \;x_{1}^{m}\\ x_{2}^{1} & \;x_{2}^{2} & \;\text{…} & \;x_{2}^{m}\\ \vdots & \;\vdots & \;\ddots & \;\vdots \\ x_{n}^{1} & \;x_{n}^{2} & \;\text{…} & \;x_{n}^{m} \end{array}\right)}_{n\times m}$$

(17) $$W={\left(\begin{array}{cccc} w_{11} & \;w_{12} & & \;w_{1n}\\ w_{21} & \;w_{22} & \;\text{…} & \;w_{2n}\\ \vdots & \;\vdots & \;\ddots & \;\vdots \\ w_{L1} & \;w_{L2} & \;\text{…} & \;w_{Ln} \end{array}\right)}_{L\times n}$$

(18) $$Z={\left(\begin{array}{cccc} z^{1} & \;z^{2} & \;\text{…} & \;z^{m}\\ z^{2} & \;z^{3} & \;\text{…} & \;z^{m+1}\\ \vdots & \;\vdots & \;\ddots & \;\vdots \\ z^{L} & \;z^{L+1} & \;\text{…} & \;z^{m+L-1} \end{array}\right)}_{L\times m}\;$$

Based on the delay embedding requirement for *Z*, the latter *m* − 1 elements in the *i*‐th row were identical to the former *m* − 1 elements in the (*i* + 1)‐th row of the matrix *Z*. In addition,

(19) $$W_{i}P=\left(z^{i+1},\;z^{i+2},\;\text{…},z^{i+m-1}\right)$$

(20) $$W_{i+1}Q=\left(z^{i+1},\;z^{i+2},\;\text{…},z^{i+m-1}\right)$$

where *P* and *Q* were two submatrices of *X*, i.e., *P* = [**x** ^2^ **x** ^3^ ⋅⋅⋅**x** ^*m*^], *Q* = [**x** ^1^ **x** ^2^ ⋅⋅⋅**x** ^*m* − 1^], and $W_{i}=\left(w_{i1},\;w_{i2},\text{…},w_{in}\right)$, 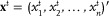, and i=1,2,…,L−1.

From Equation (19) and Equation (20), the following equation could be obtained:

(21) $$W_{i}P=W_{i+1}\;Q$$

### The stPCA Algorithm

Given a high‐dimensional time series *X*, the box‐counting dimension *d* of the system's dynamics was estimated using the false nearest neighbor algorithm^[^ ^70^ ^]^ and the embedding dimension *L* > 2*d* > 0 was chosen. The target was to obtain a single variable *z* from high‐dimensional time‐series data to represent the dynamics of the whole system. The dynamics of such a single variable were analytically derived, with the main points listed as follows:

1). A linear transformation *Z* = *WX* was used, e.g., **Z**_*i*_ = **W**_*i*_ *X*. At first, the sample mean of *X* was removed before performing a projection, similar to PCA. Then, the loss function was shown as

(22) $$\underset{W\;}{\mathrm{minimize}\;\;}-\left(1-\lambda \right)\sum_{i=1}^{L}∥W_{i}X{∥}_{2}^{2}+\lambda \;\sum_{i=1}^{L-1}∥W_{i}P-W_{i+1}Q{∥}_{2}^{2}$$

(23) $$s.t.\;\;\sum_{i=1}^{L}W_{i}W_{i}^{T}=1$$

where λ was a predetermined regularization parameter between 0 and 1.

2). On the basis of the loss function, the Lagrange function was as follows:

(24) $$\begin{array}{ccc} L\;(W,\;\Lambda ) & = & -\left(1-\lambda \right)\sum_{i=1}^{L}∥W_{i}X{∥}_{2}^{2}+\lambda \sum_{i=1}^{L-1}∥W_{i}P-W_{i+1}Q{∥}_{2}^{2}\\ & & +\;\Lambda \left(\sum_{i=1}^{L}W_{i}W_{i}^{T}-1\right) \end{array}$$

where $\Lambda \in R^{nL\times nL}$ was a Lagrange matrix.

3). By taking the partial derivative of *L*(*W*, Λ) with respect to **W**_*i*_ and Λ, yields

(25) $$\frac{\partial L\left(W,\Lambda \right)}{\partial \;W_{1}}=-2\left(\left(\left(1-\lambda \right)\cdot XX^{T}-\lambda PP^{T}\right)W_{1}^{T}+\lambda PQ^{T}W_{2}^{T}\right)+2\Lambda_{1}W_{1}^{T}$$

(26) $$\begin{array}{ccc} \frac{\partial L\left(W,\Lambda \right)}{\partial \;W_{i}} & = & -2\left(\lambda QP^{T}W_{k-1}^{T}+\left(\left(1-\lambda \right)\cdot XX^{T}-\lambda PP^{T}-\lambda QQ^{T}\right)W_{k}^{T}\right.\\ & & +\left.\lambda PQ^{T}W_{k+1}^{T}\right)+2\Lambda_{i}W_{i}^{T} \end{array}$$

where

(27) 2≤i≤L−1

(28) $$\begin{array}{c} \frac{\partial L\left(W,\Lambda \right)}{\partial \;W_{L}}=-2\lambda \left(QP^{T}W_{L-1}^{T}+\left(\left(1-\lambda \right)\cdot XX^{T}-\lambda QQ^{T}\right)W_{L}^{T}\right)+2\Lambda_{L}W_{L}^{T} \end{array}$$

4). To minimize the loss function above or in Equation (2), it was set $\frac{\partial L(W,\Lambda )}{\partial \;W_{1}}=0$, $\frac{\partial L(W,\Lambda )}{\partial \;W_{i}}=0$, $\frac{\partial L(W,\Lambda )}{\partial \;W_{L}}=0$, and let **V** = vec(*W*). Then, the following characteristic equation was obtained:

(29) $$H\left(X\right)V=\alpha V$$

where *H* was a function converting *X* to a block‐tridiagonal matrix; i.e.,

(30) $$\begin{array}{c} H\left(X\right)=\left(\begin{array}{ccccccc} \left(1-\lambda \right)XX^{T}-\lambda PP^{T} & \lambda PQ^{T} & 0 & \text{…} & 0 & 0 & 0\\ \lambda QP^{T} & \left(1-\lambda \right)XX^{T}-\lambda PP^{T}-\lambda QQ^{T} & \lambda PQ^{T} & \text{…} & 0 & 0 & 0\\ \text{…} & \text{…} & \text{…} & \text{…} & \text{…} & \text{…} & \text{…}\\ 0 & 0 & 0 & \text{…} & \lambda QP^{T} & \left(1-\lambda \right)XX^{T}-\lambda PP^{T}-\lambda QQ^{T} & \lambda PQ^{T}\\ 0 & 0 & 0 & \text{…} & 0 & \lambda QP^{T} & \left(1-\lambda \right)XX^{T}-\lambda QQ^{T} \end{array}\right) \end{array}$$

Here, α and ***V*** denote the dominant eigenvalue and a corresponding eigenvector of *H*(*X*), respectively.

5). *W* was obtained by reshaping the vector **V** by  = *reshape*(**V**) · α.

6). There was

(31) $$\hat{Z}=WX$$

A Hankel matrix *Z* was derived from $\hat{Z}$ in Equation (31), and the one‐dimensional representation **z** = (*z* ^1^,*z* ^2^,⋅⋅⋅, *z^m^*,*z* ^*m* + 1^, ⋅⋅⋅, *z* ^*m* + *L* − 1^) could be easily obtained by expanding *Z*. Detailed information about the stPCA algorithm was presented in Note S3 (Supporting Information). Theoretically, the dynamics of the original system could be topologically reconstructed by this delay embedding matrix *Z* with one variable.

In addition, stPCA reduces to a linear representation when λ was 0, and the value of λ controls the tradeoff between nonlinear regularization, which enhances adaptability for delay embedding and noise resistance, and the preservation of the interpretation within the corresponding linear representation. It was noted that compared with the linear representation, choosing large values of λ may lead to some distortion and compression of the data span. Smooth interpolation between any stPCA representation and the corresponding linear representation can be obtained by generating a series of representations by gradually reducing λ to 0.

### Detecting Early‐Warning Signals of Tipping Points Just Before Critical State Transitions in a High‐Dimensional Dynamical System

A discrete‐time dynamical system in generic form was considered

(32) $$X\left(t\right)=f\left(X\left(t-1\right);P\right)$$

where $X\;\left(t\right)=\left(x_{1}\left(t\right),\text{…},x_{n}\left(t\right)\right)$ was an *n*‐dimensional state vector or variable at time instant *t* representing feature values and $P=(p_{1},\text{…},p_{s})$ was a parameter vector or driving factor representing slowly changing factors. $f:R^{n}\times R^{s}\to R^{n}$ was a nonlinear function.^[^ ^44^ ^]^ Furthermore, the following conditions were assumed to hold for Equation (32): i). $\bar{X}$ was a fixed point of the system such that $\bar{X}=f\left(\bar{X};P\right)$. ii). There was a value **P**_*c*_ such that one or a pair of eigenvalues of the Jacobian matrix $\frac{\partial \;f(X;P_{c})}{\partial \;X}{|}_{X=\bar{X}\;}$ equals 1 in modulus. iii) When $P\neq P_{c}$, the eigenvalues of system were not always 1 in modulus. These three conditions with other transversal conditions, imply that the system undergoes a phase change at $\bar{X}$ or a codimension‐one bifurcation when **P** reaches the threshold **P**_*c*_.

For system Equation (32) near $\bar{X}$, before **P** reaches **P**_*c*_, the system was supposed to stay at a stable fixed point $\bar{X}$, and therefore, all the eigenvalues were within (0, 1) in modulus. The parameter value **P**_*c*_ at which the state shift of the system occurs was called a bifurcation parameter value or a critical transition value. Now, the linearized approximate equations of Equation (32) were considered. Specifically, by introducing new variables $Y\;\left(t\right)=\left(y_{1}\left(t\right),\text{…},y_{n}\left(t\right)\right)$ and a transformation matrix *A* = (*a_ij_*)_*n* × *n*_, i.e., $Y\;\left(t\right)=A^{-1}\;\left(X\left(t\right)-\bar{X}\right)$, we have

(33) $$Y\left(t\right)=\Gamma \left(P\right)Y\left(t-1\right)+\zeta \left(t-1\right)$$

where $\zeta =(\zeta_{1},\text{…},\zeta_{n})$ denotes small Gaussian noise with zero means. ζ_*i*_ has a small standard deviation σ_*i*_ for all *i*.

Without loss of generality, the diagonalized matrix $\Gamma =\;\mathrm{diag}(\gamma_{1},\text{…},\gamma_{n})$ was assumed to have each γ_*i*_ between 0 and 1. Among the eigenvalues of Γ, the largest one (in modulus), say γ_1_, first approaches 1 in modulus when parameter transition **P** → **P**_*c*_ occurs. The dominant eigenvalue γ_1_ characterized the system's rate of change around the fixed point and was called the dominant eigenvalue. The before‐transition state corresponds to the period when |γ_1_| < 1, whereas the critical/pre‐transition stage corresponds to the period when |γ_1_| → 1. Without the loss of generality, the first variable *y*_1_ in **Y** was assumed to be associated with the dominant eigenvalue γ_1_. It was clear that the variance was of the form:

(34) $$\mathrm{Var}\;\left(y_{i}\right)=\frac{\sigma_{i}^{2}}{\left|1-\gamma_{i}^{2}\right|}\;$$

From Equation (34), the standard deviations of variables $x_{i}=a_{i1}\;y_{1}+\cdots +a_{in}y_{n}+\bar{x_{i}}$ with $a_{i1}\neq 0$, increase sharply as the dominant eigenvalue |γ_1_| → 1. However, such *x_i_* with $a_{i1}\neq 0$ were difficult to identify based on only time‐series data (without a definite model).

The single latent variable *z* obtained by stPCA can be regarded as *y*_1_ of an abstract manifold/center manifold. Therefore, when only high‐dimensional time series were observed, the SD of the representative variable *z* of stPCA could be utilized to evaluate the state when a dynamical system approaches a bifurcation point from a stable steady state.

### Data and materials availability

The codes of stPCA for two simulation datasets and three real‐world applications were available from https://github.com/RPcb/stPCA. The raw data of MIMIC‐III and MIMIC‐IV were available from https://physionet.org/content/mimiciii/1.4/ and https://physionet.org/content/mimiciv/2.2/. The continuous glucose monitoring (CGM) dataset was from https://doi.org/10.1371/journal.pone.0225817.s001. The dataset of single‐cell embryonic development was deposited in NCBI's Gene Expression Omnibus and accessible through https://www.ncbi.nlm.nih.gov/geo/query/acc.cgi?acc=GSE75748. The signaling pathway analysis was carried out based on the Kyoto Encyclopedia of Genes and Genomes (KEGG).

## Conflict of Interest

The authors declare that they have no conflict of interest.

## Author Contributions

P.C., R.L., and L.C. designed the research; P.C., R.L., and K.A. performed the research; Y.L. and D.W. provided the MIMIC data; P.C. and Y.S. analyzed the data; and P.C., K.A., R.L., and L.C. wrote the paper.

## Supporting information



Supporting Information

## Data Availability

The data that support the findings of this study are available in the supplementary material of this article.;
