# Supplementary material for: Ultralow‐Dimensionality Reduction for Identifying Critical Transitions by Spatial‐Temporal PCA
Source: Adv Sci (Weinh). 2025 Apr 25;12(20):2408173. doi: 10.1002/advs.202408173 (PMC12120726; doi:10.1002/advs.202408173)
Supplement: Supplementary file 1 — Supporting Information [file ADVS-12-2408173-s001.pdf]

ADVANCED SCIENCE
Open Access

Supporting Information

for *Adv. Sci.*, DOI 10.1002/advs.202408173

Ultralow-Dimensionality Reduction for Identifying Critical Transitions by Spatial-Temporal PCA

Pei Chen, Yaofang Suo, Kazuyuki Aihara, Ye Li, Dan Wu, Rui Liu and Luonan Chen**

Supplementary Information for

Ultralow-dimensionality reduction for identifying critical transitions by spatial-temporal PCA

Chen et al.

*Correspondence: Luonan Chen, lnchen@sibcb.ac.cn; Rui Liu, scliurui@scut.edu.cn

This PDF file includes:

Figs. S1 to S14
Tables S1 to S4
Supplementary Notes S1-S10

Table of Contents

Supplementary Information for *1*

Ultralow-dimensionality reduction for identifying critical transitions by spatial-temporal PCA *1*

Section S1. Supplementary figures ***3***

Figure S1. Heatmaps of projections Z and principal components (W) 3

Figure S2. Results of input with randomly ordered samples 4

Figure S3. Results of ultrashort time series from stPCA and PCA 5

Figure S4. Results of longer time series from a coupled Lorenz system 6

Figure S5. Results from a coupled Lorenz system with different noise strengths 7

Figure S6. Performances of stPCA and a numerical solution SQP for coupled Lorenz systems 8

Figure S7. Results of DREAM4 from stPCA and PCA 9

Figure S8. Networks with 16 nodes under different parameter q values 10

Figure S9. Networks with 18 nodes under different parameter p values 11

Figure S10. Performances of stPCA and a numerical solution SQP for multi-node networks 12

Figure S11. Preprocessing of the MIMIC datasets 13

Figure S12. The sliding window scheme 14

Figure S13. The ICU discharge scheme based on stPCA 15

Figure S14. The preprocessing of the original records into patient-specific high-dimensional time series for the MIMIC datasets 16

Section S2. Supplementary tables ***17***

Table S1. PCFDs for the top three projections of a coupled Lorenz system with different values of L 17

Table S2. The relationship between parameters q and τ for the 16-node network model 17

Table S3. The relationship between parameters p and τ for the 18-node network model 18

Table S4. The 2-5 most relevant biometric items for the diagnoses/diseases 19

Section S3. Supplementary Notes 20

Supplementary Note S1. Dynamical systems and delay embedding theorem 20

Supplementary Note S2. Spatiotemporal information (STI) transformation equations 20

Supplementary Note S3. Details of the stPCA algorithm 21

Supplementary Note S4. Coupled Lorenz systems 23

Supplementary Note S5. The details of the numerical simulation on multiple-node networks 24

Supplementary Note S6. Evaluation Metrics 29

Supplementary Note S7. Discrete Fréchet distance 30

Supplementary Note S8. Dynamic time warping 31

Supplementary Note S9. Methods for comparison 32

Supplementary Note S10. The comparisons of time complexity between DNN methods and stPCA 33

References 34

Section S1. Supplementary figures

Figure S1. Heatmaps of projections $\hat{\boldsymbol{Z}}$ and principal components ($\boldsymbol{W}$)

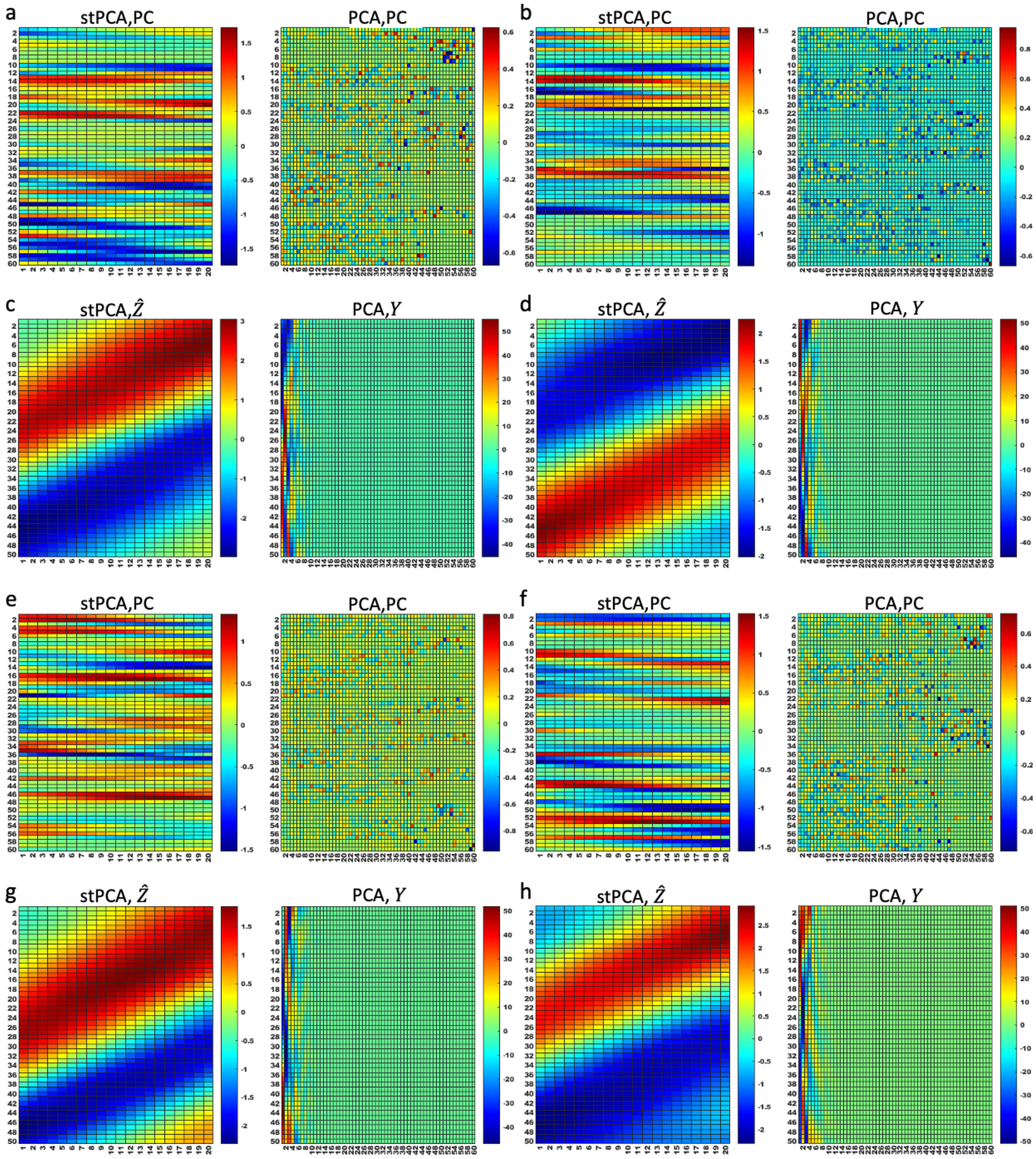

Figure S1. The heatmaps of projections $\hat{Z}$ for stPCA and Y for PCA, and principal components (W). We presented the heatmaps of 4 different cases from a coupled Lorenz system by stPCA and PCA ($m = 50$, $L = 20$, $\sigma = 0$, and $n = 90$). (a) Principal components (PC) of Case 1; (b) Principal components of Case 2; (c) Projections of Case 1; (d) Projections of Case 2; (e) Principal components of Case 3; (f) Principal components of Case 4; (g) Projections of Case 3; (h) Projections of Case 4.

Figure S2. Results of input with randomly ordered samples

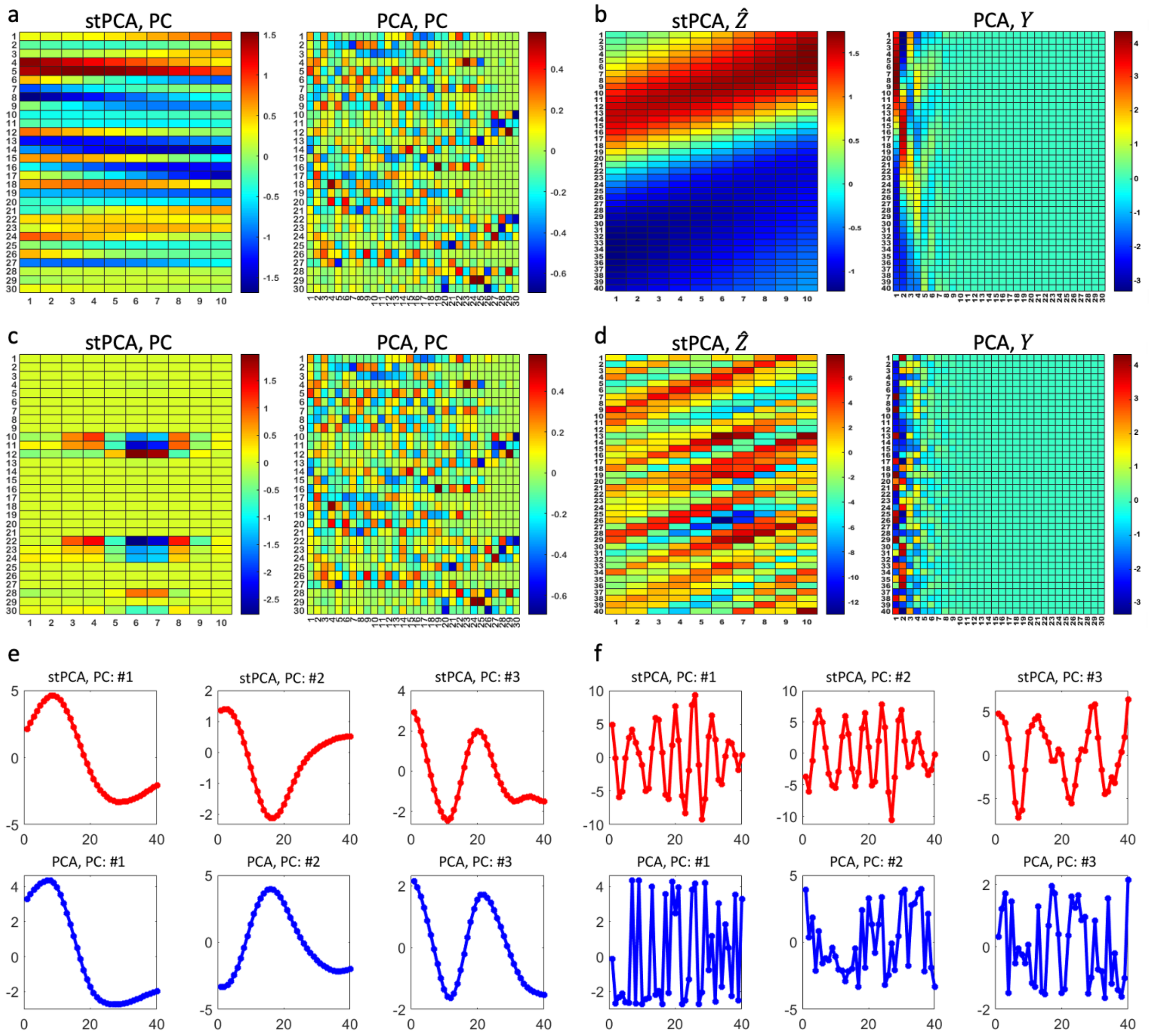

Figure S2. Results of input with random order of samples. We adopted a high-dimensional time series from a coupled Lorenz system. (a) Heatmaps of principal components (PC) with the original order of samples. (b) Projections of principal components with the original order of samples. (c) Heatmaps of principal components with randomly ordered samples. (d) Projections of principal components with randomly ordered samples. (e) For the original order, the red curves above display projections of the three largest principal components obtained from the Hankel matrix of stPCA through SVD, while the green curves below display the projections of the three largest principal components obtained from PCA. (f) For the random order, the red curves above display projections of the three largest principal components obtained from the Hankel matrix through SVD, while the green curves below display the projections of the three largest principal components obtained from PCA.

Figure S3. Results of ultrashort time series from stPCA and PCA

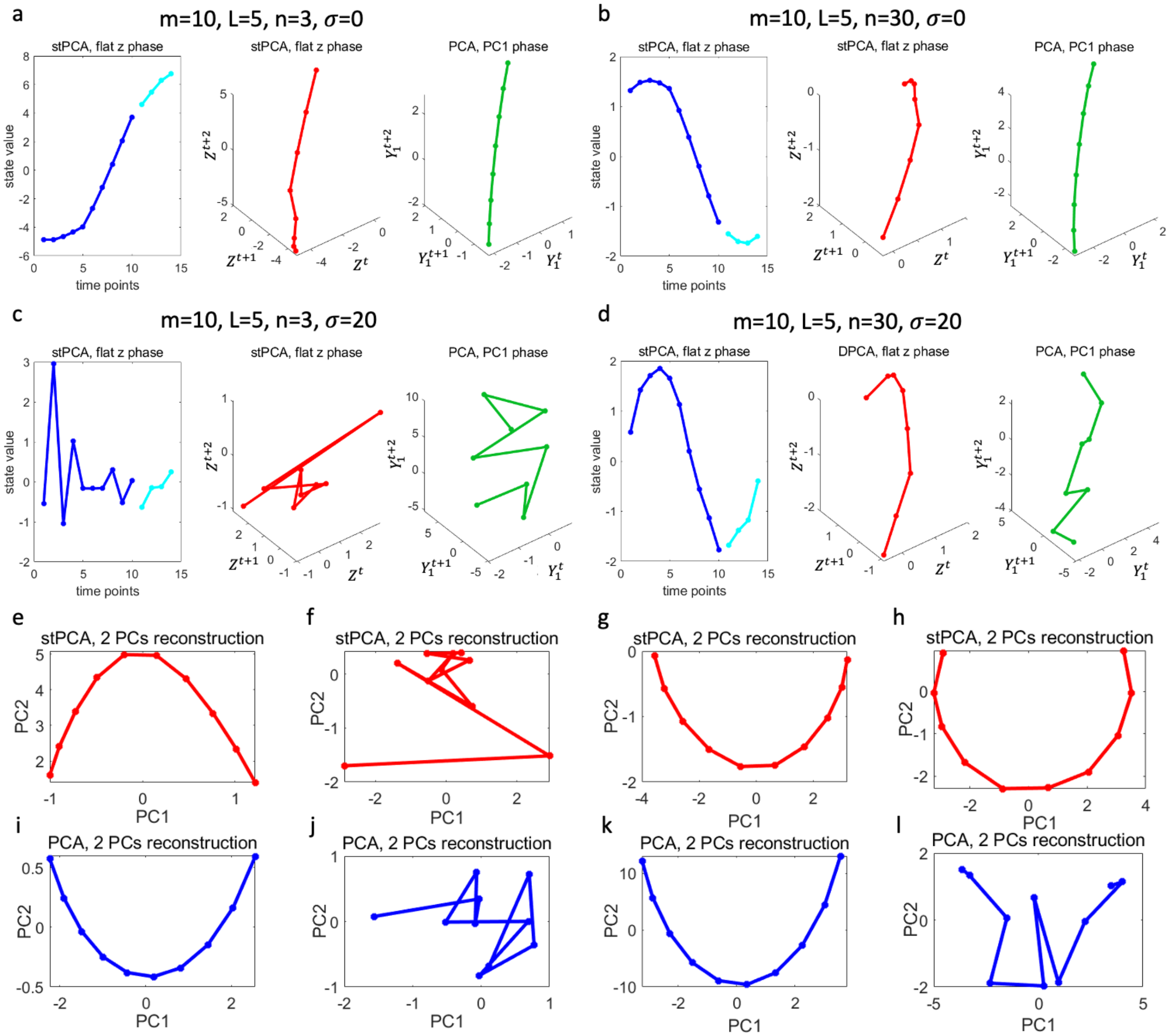

Figure. S3. Results of ultrashort time series. (a) z and phases of a case with $m = 10, L = 5, n = 3$, noise strength $\sigma = 0$, where flat z refers to one-dimensional dynamics of variable z; (b) z and phases of a case with $m = 10, L = 5, n = 30$, noise strength $\sigma = 0$; (c) z and phases of a case with $m = 10, L = 5, n = 3$, noise strength $\sigma = 20$; (d) z and phases of a case with $m = 10, L = 5, n = 30$, noise strength $\sigma = 20$; (e) Principal component projections resulting from stPCA with $n = 3$, noise strength $\sigma = 0$; (f) Principal component projection results from stPCA with $n = 3$, noise strength $\sigma = 30$; (g) Principal component projection results from stPCA with $n = 30$, noise strength $\sigma = 0$; (h) Principal component projection results from stPCA with $n = 30$, noise strength $\sigma = 30$; (i) Principal component projections resulting from PCA with $n = 3$, noise strength $\sigma = 0$; (j) Principal component projection results from PCA with $n = 3$, noise strength $\sigma = 30$; (k) Principal component projection results from PCA with $n = 30$, noise strength $\sigma = 0$; (l) Principal component projection results from PCA with $n = 30$, noise strength $\sigma = 30$.

Figure S4. Results of longer time series from a coupled Lorenz system

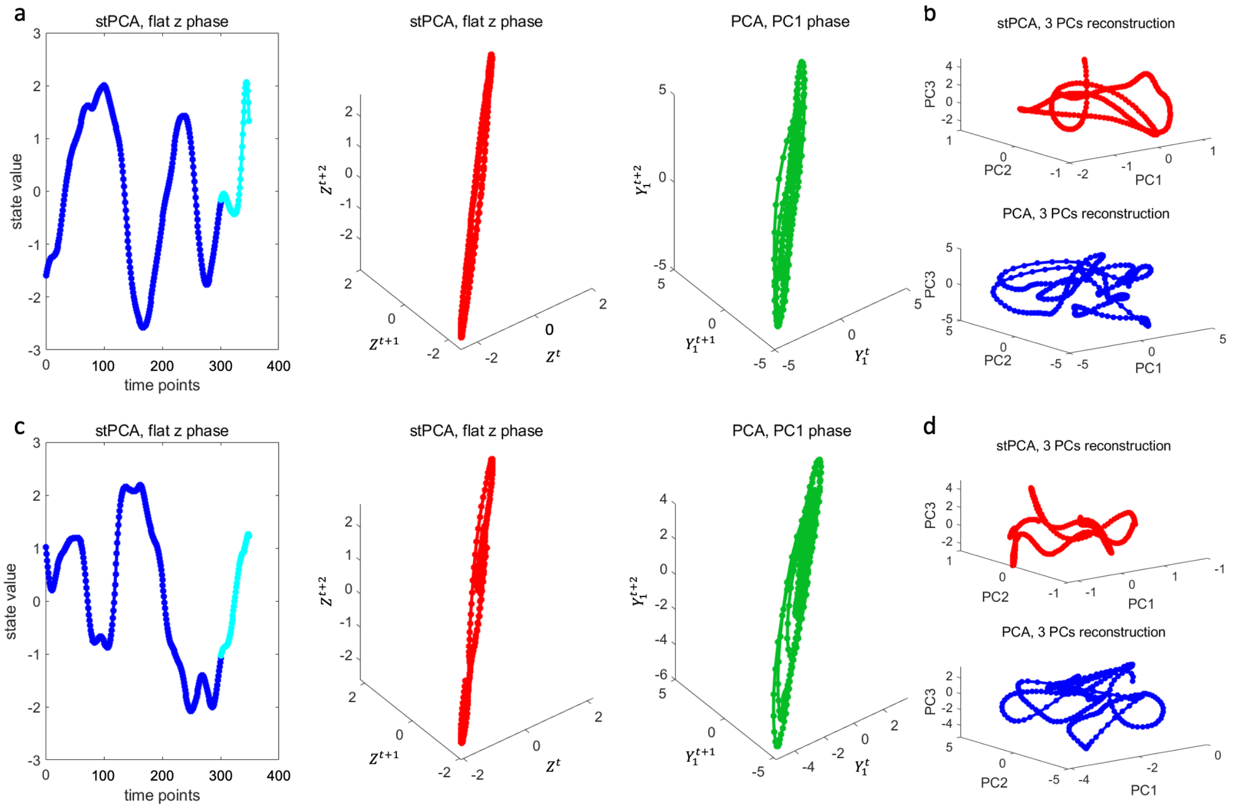

Figure S4. Results of longer time series from a coupled Lorenz system with $m = 300, L = 50$, and $n = 90$. (a) z and phases of Case 1 by stPCA and PCA; (b) Projections for the three largest principal components (PC) of Case 1 by stPCA and PCA; (c) z and phases of Case 2 by stPCA and PCA; (d) Projections for the three largest principal components of Case 2 by stPCA and PCA.

Figure S5. Results from a coupled Lorenz system with different noise strengths

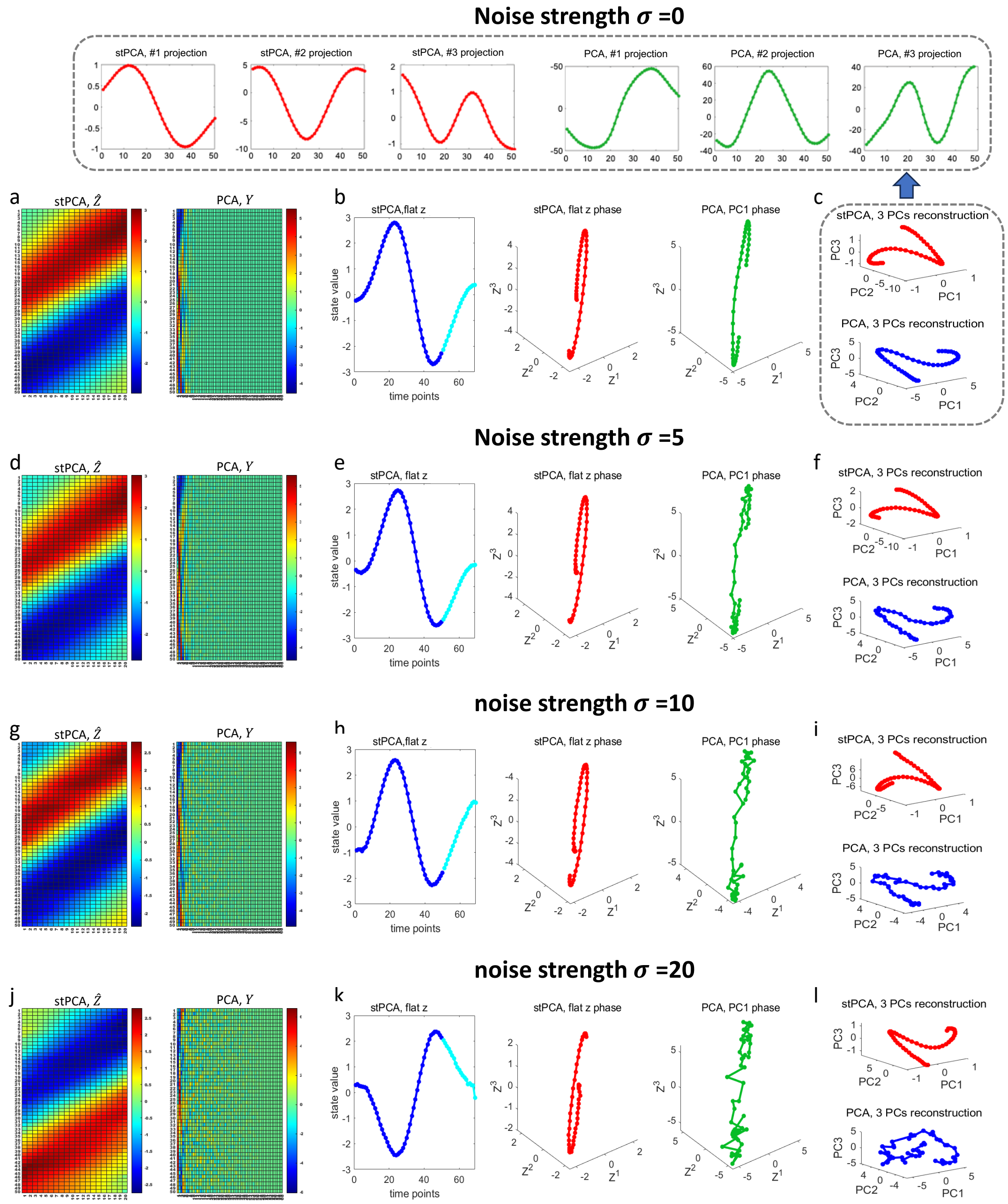

Figure S5. Results from a coupled Lorenz system with different noise strengths. (a) Heatmaps of projections with noise strength σ =0 by stPCA and PCA; (b) z and phases with noise strength σ =0; (c) Projections for the three largest principal components (PC) with noise strength σ =0 by stPCA and PCA; (d) Heatmaps of projections with noise strength σ =5 by stPCA and PCA; (e) z and phases with noise strength σ =5; (f) Projections for the three largest principal components with noise strength σ =5 by stPCA and PCA; (g) Heatmaps of projections with noise strength σ =10 by stPCA and PCA; (h) z and phases with noise strength σ =10; (i) Projections for the three largest principal components with noise strength σ =10 by stPCA and PCA; (j) Heatmaps of projections with noise strength σ =20 by stPCA and PCA; (k) z and phases with noise strength σ =20; (l) Projections for the three largest principal components with noise strength σ =20 by stPCA and PCA.

Figure S6. Performances of stPCA and a numerical solution SQP for coupled Lorenz systems

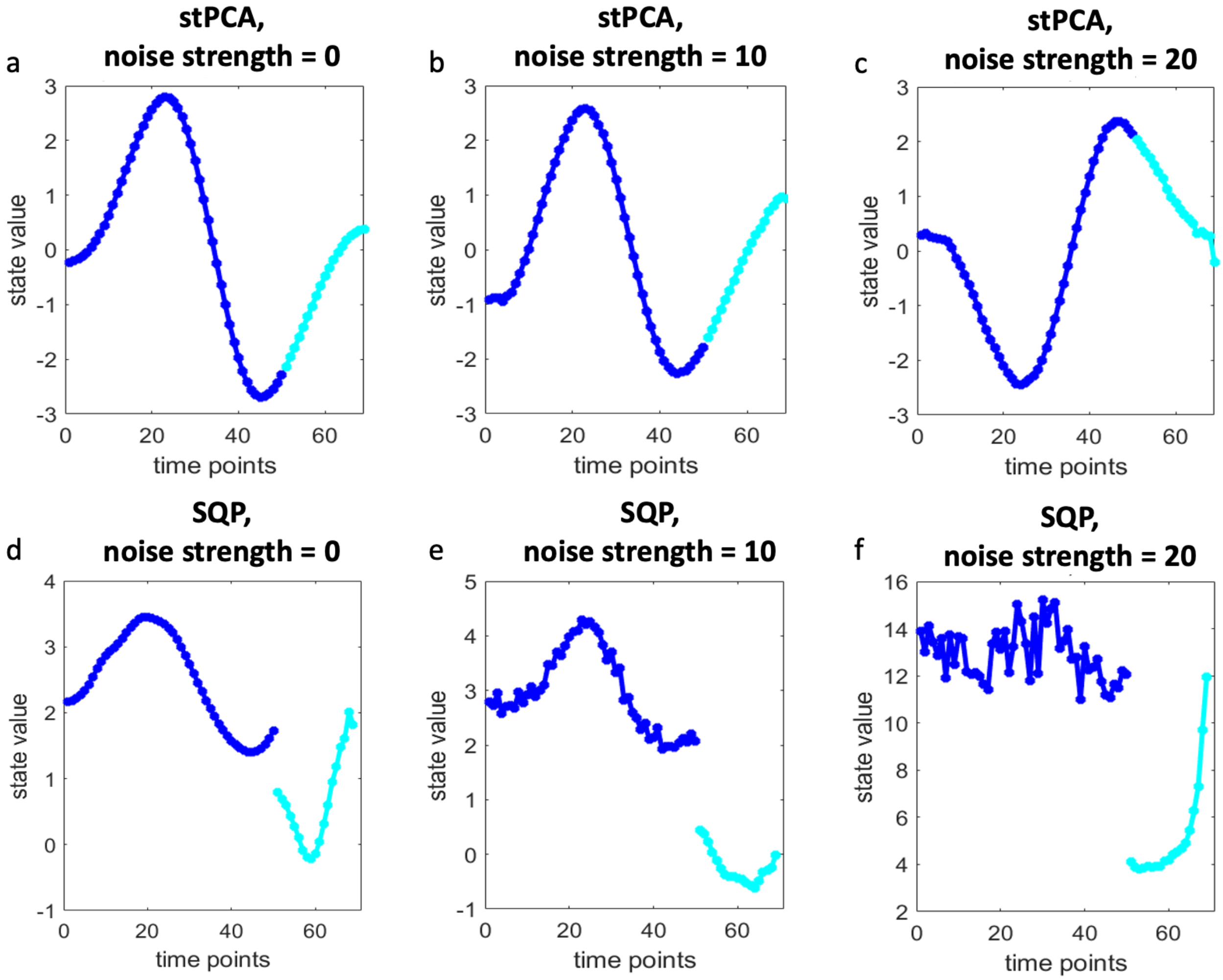

Figure S6. Performances of stPCA and a numerical solution SQP for coupled Lorenz systems. (a) z obtained by stPCA with noise strength σ =0; (b) z obtained by stPCA with noise strength σ =10; (c) z obtained by stPCA with noise strength σ =20; (d) z obtained by a numerical solution of sequential quadratic programming (SQP)[1] with noise strength σ =0; (e) z obtained by a numerical solution of SQP with noise strength σ =10; (f) z obtained by a numerical solution of SQP with noise strength σ =20.

Figure S7. Results of DREAM4 from stPCA and PCA

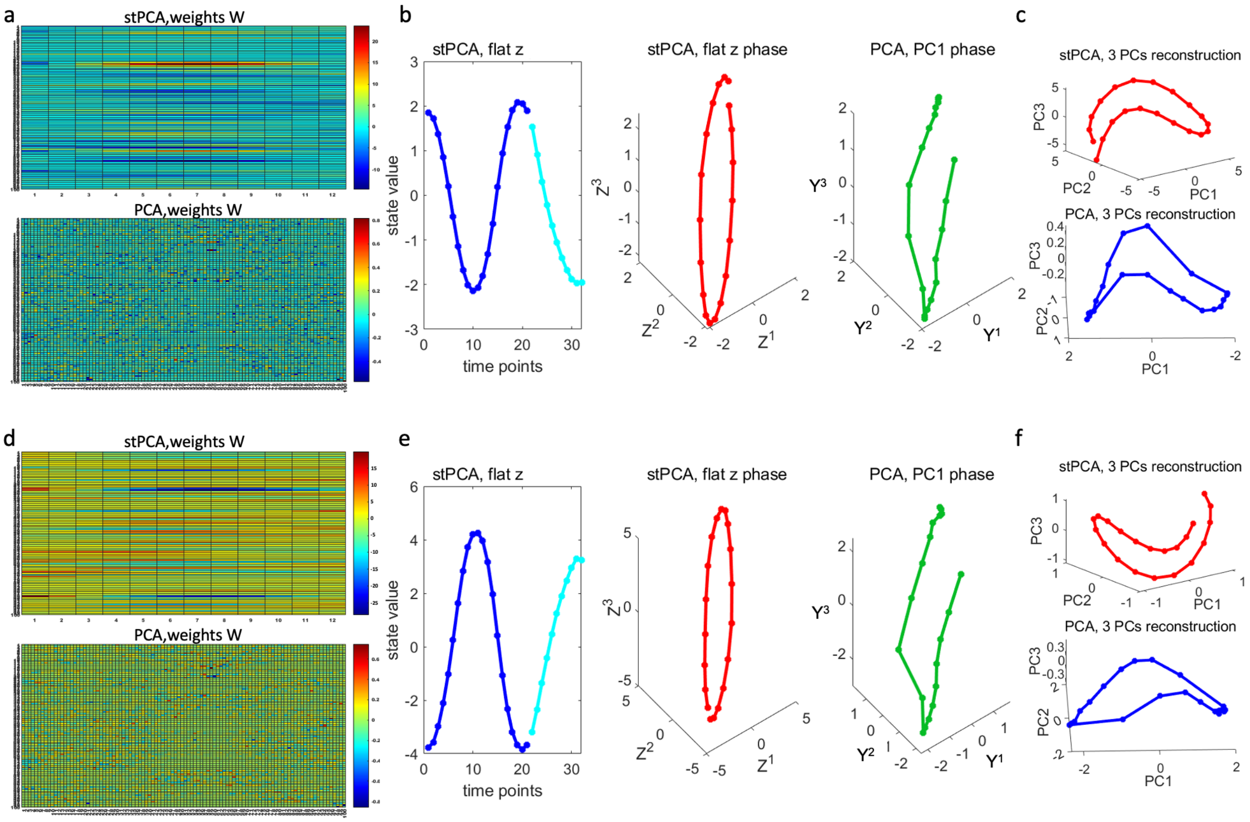

Figure S7. Results of DREAM4 from stPCA and PCA. (a) Heatmaps of principal components (i.e., weights W) of Case 1 by stPCA and PCA; (b) z and phases of Case 1, where flat z refers to one-dimensional dynamics of variable z; (c) Projections for the three largest principal components (PC) of Case 1 by stPCA and PCA; (d) Heatmaps of principal components (W) of Case 2 by stPCA and PCA; (e) z and phases of Case 2; (f) Projections for the three largest principal components of Case 2 by stPCA and PCA.

Figure S8. Networks with 16 nodes under different parameter q values

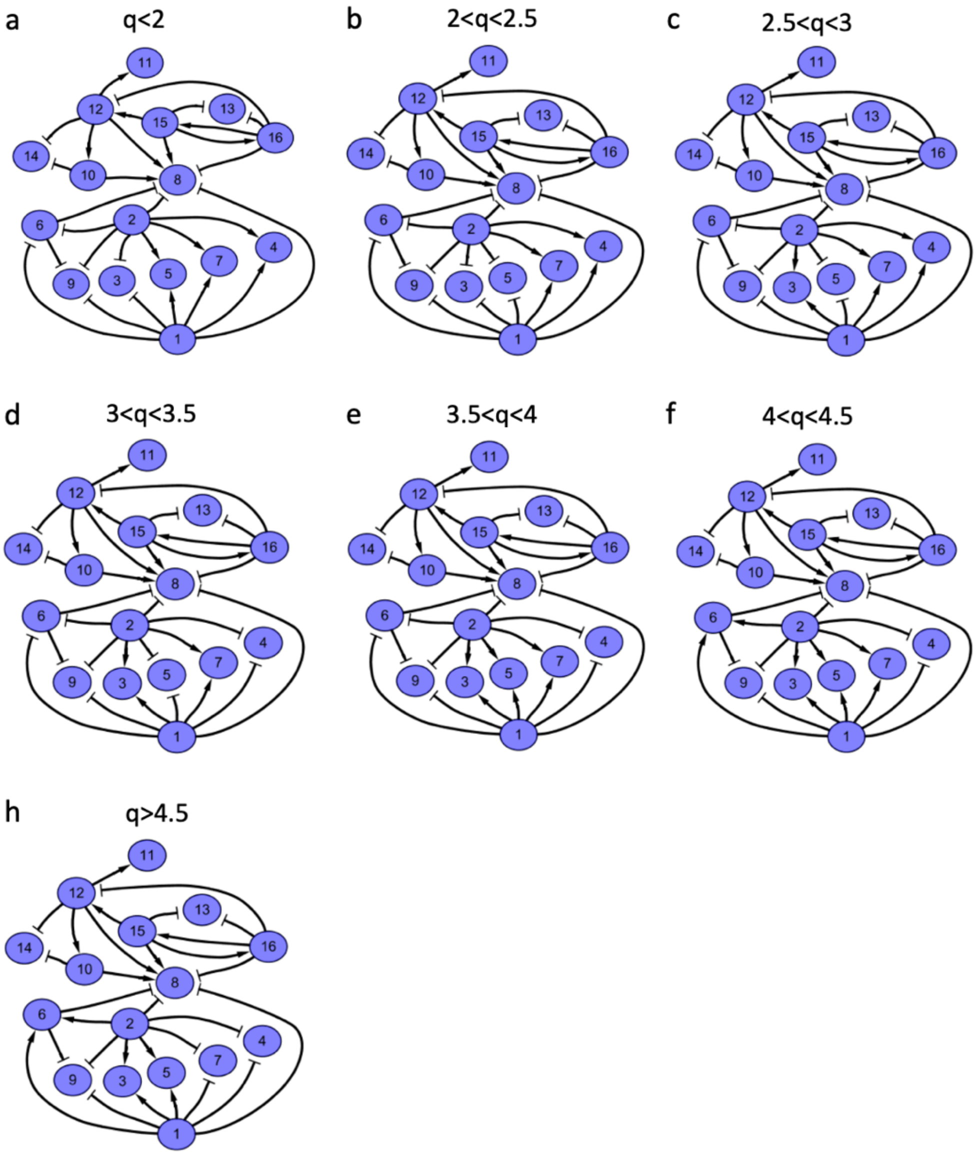

Figure S8. Networks with 16 nodes under different parameter q values. The arrow denotes activation and the blunted line indicates repression.

Figure S9. Networks with 18 nodes under different parameter $\boldsymbol{p}$ values

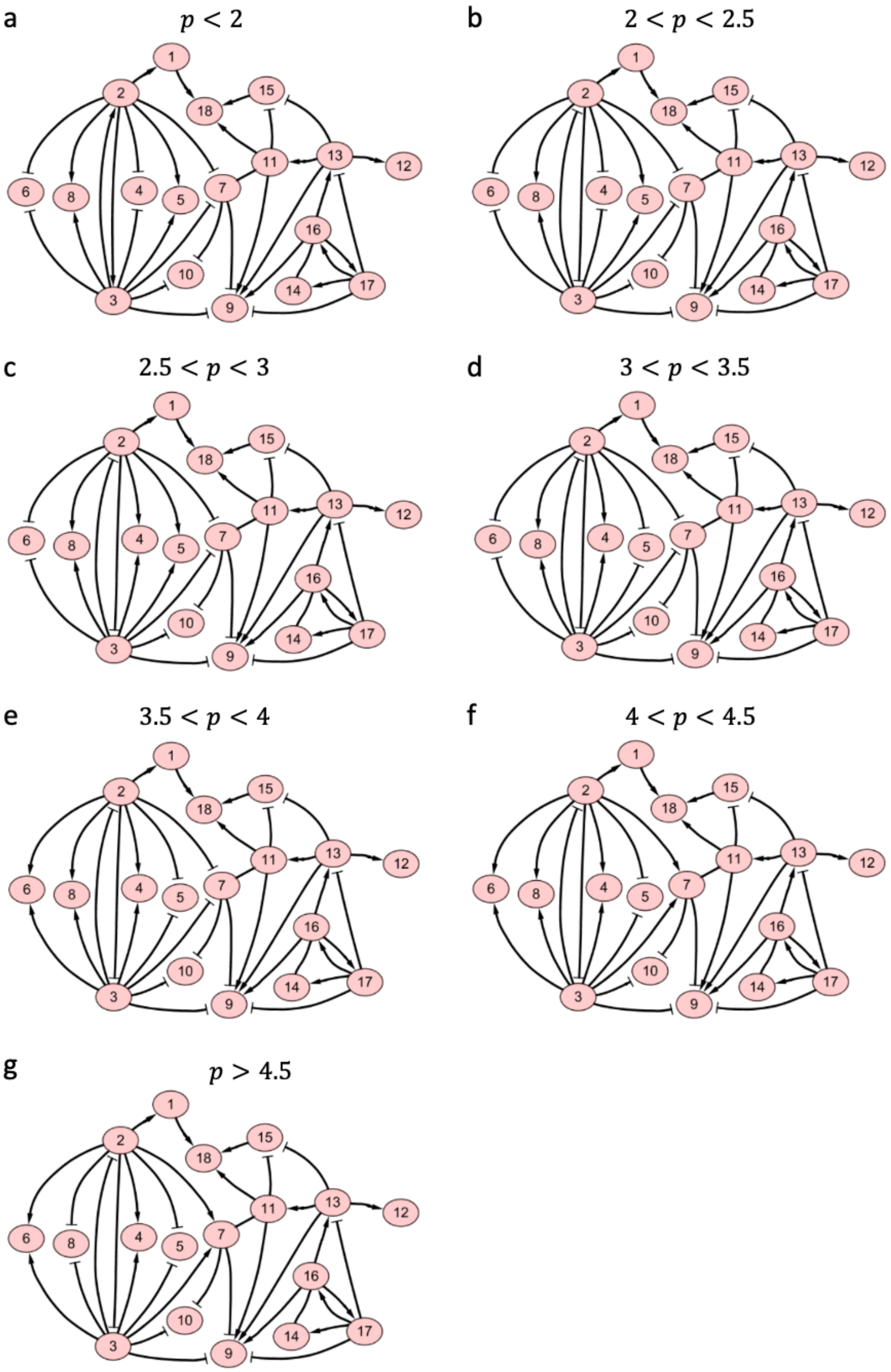

Figure S9. Networks with 18 nodes under different parameter p values. The arrow denotes activation and the blunted line indicates repression.

Figure S10. Performances of stPCA and a numerical solution SQP for multi-node networks

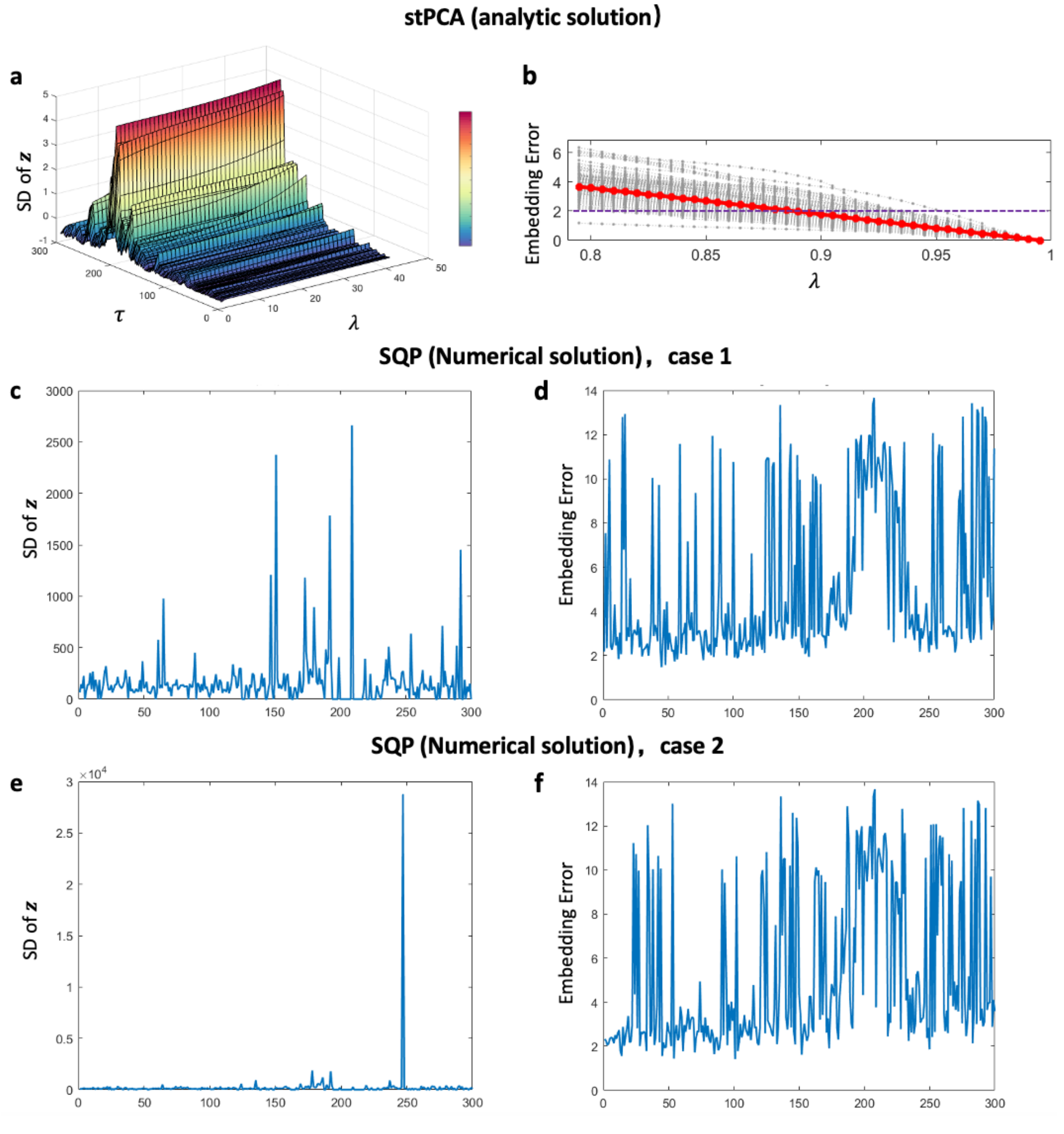

Figure S10. Performances of stPCA and a numerical solution SQP for multi-node networks. (a) The standard deviation (SD) of the variable z by stPCA with varying parameter τ and regularization parameter λ for the 16-node artificial system; (b) The average embedding error by stPCA as λ varies; (c) (e) The SD of the variable z by SQP with varying parameter τ for the 16-node artificial system for Case 1 and Case 2, respectively; (d) (f) The average embedding error by SQP with varying parameter τ for Case 1 and Case 2, respectively.

Figure S11. Preprocessing of the MIMIC datasets

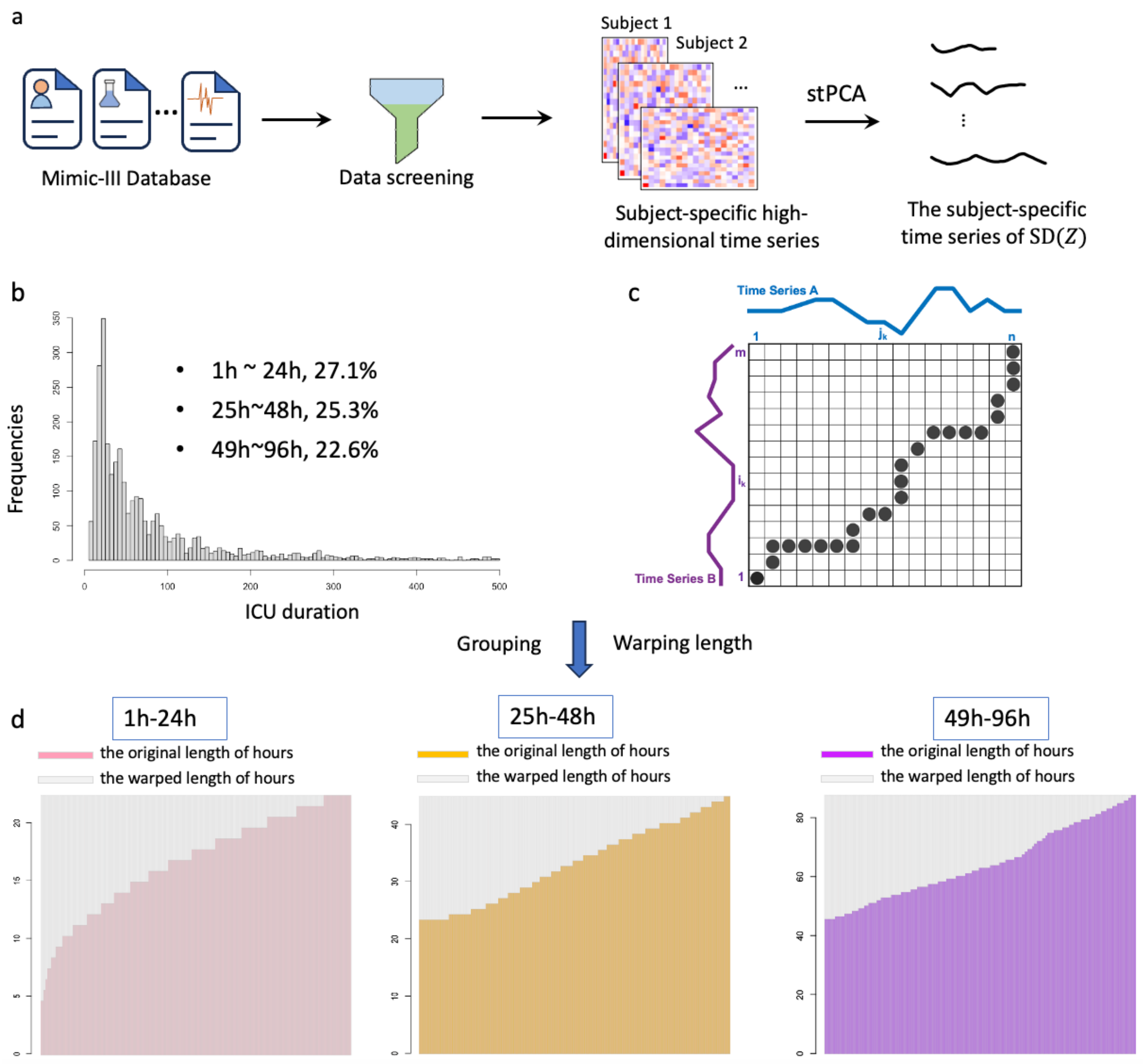

Figure S11. Preprocessing of the MIMIC-III dataset. (a) Data screening and dimensionality reduction by stPCA for each subject; (b) The ICU duration distribution of all filtered samples; (c) Schematic diagram of the dynamic time warping (DTW) algorithm; (d) The original lengths and warped lengths by the DTW algorithm for each group.

Figure S12. The sliding window scheme

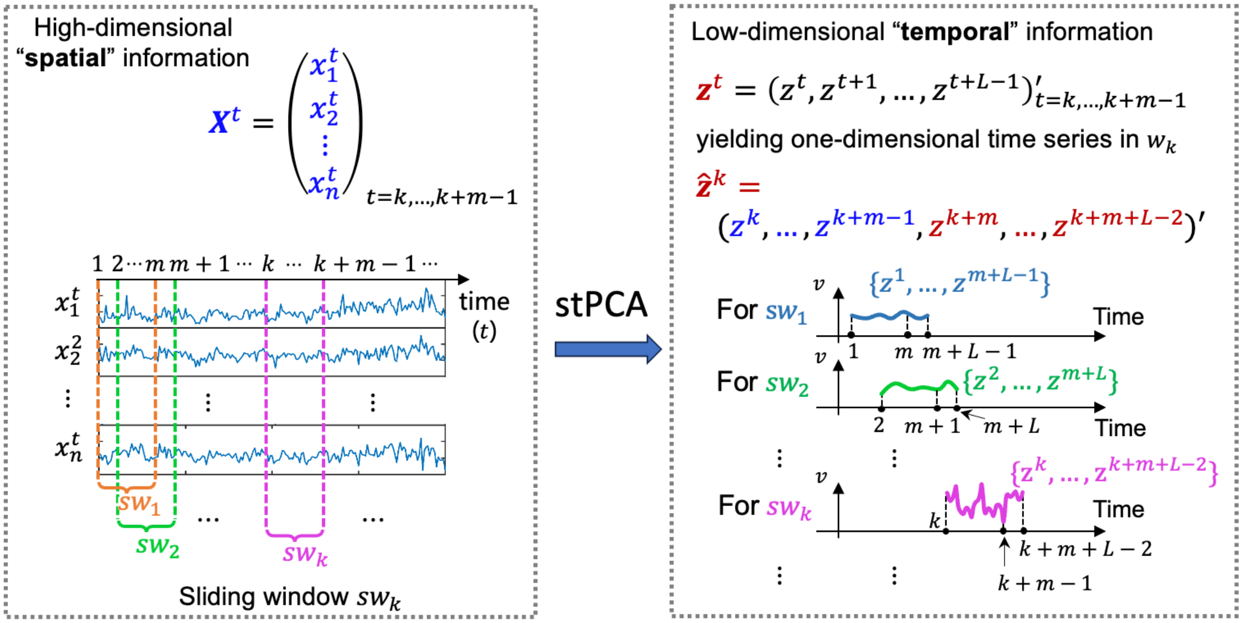

Figure S12. The sliding window scheme. The given high-dimensional "spatial" information $\boldsymbol{X}^t$ is transformed into one-dimensional "temporal" information $\boldsymbol{z}^t$ by stPCA.

Figure S13. The ICU discharge scheme based on stPCA

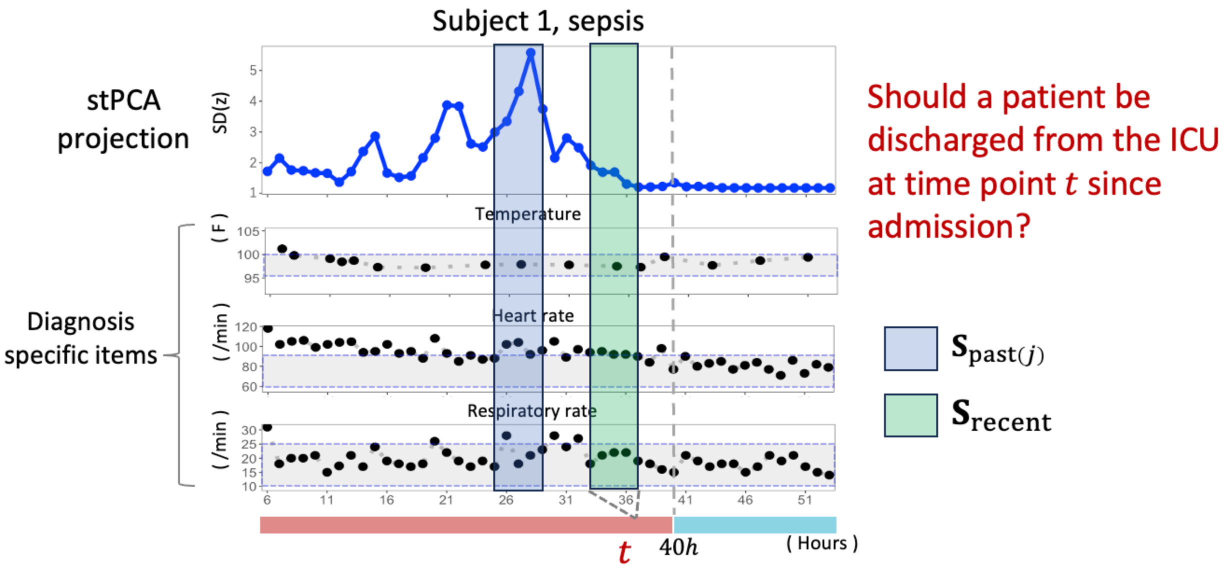

$$\mathrm{idx}_z(t) \;=\; \max\left(\mathrm{mean}\left(\mathbf{S}_{\mathrm{past}(j)}\right)\right) \,/\, \mathrm{mean}\left(\mathbf{S}_{\mathrm{recent}}\right)$$

$$itmFlg(t) = \sum_{i=1}^{k} Lb_i \leq x_i^t \leq Ub_i\,,$$

$$\mathrm{Decision}(t) = \begin{cases} 1, & \mathrm{idx}_z(t) \geq FC \text{ and } \mathrm{itmFlg}(t) = K, \quad \text{Stay} \\ 0, & \text{otherwise,} \quad \text{Discharge} \end{cases}$$

where FC is a fold change, K is the number of diagnosis specific items.

Figure S14. The preprocessing of the original records into patient-specific high-dimensional time series for the MIMIC datasets

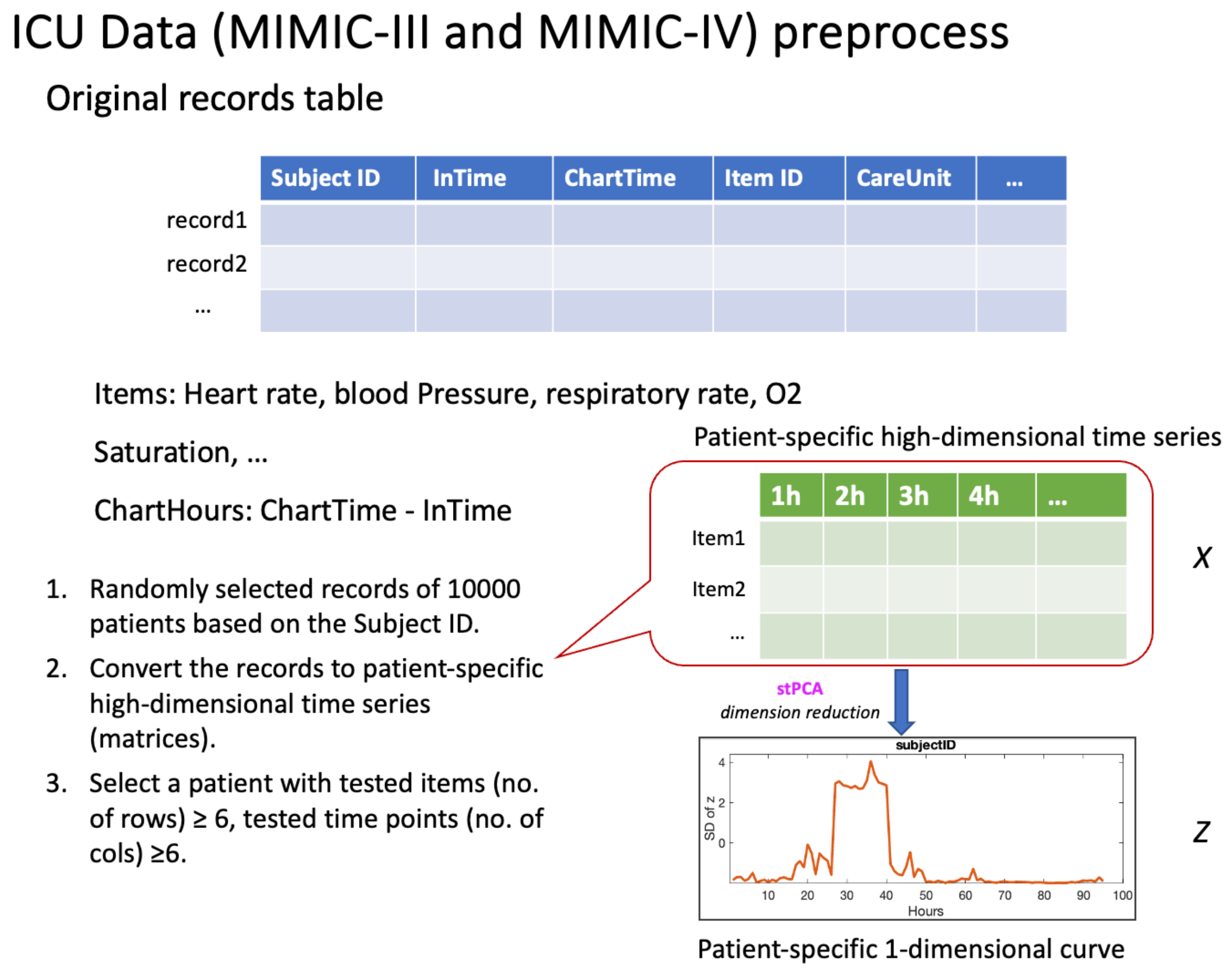

Section S2. Supplementary tables

Table S1. PCFDs for the top three projections of a coupled Lorenz system with different values of $\boldsymbol{L}$

L	zPC1 distance	zPC2 distance	zPC3 distance	L	zPC1 distance	zPC2 distance	zPC3 distance
4	0.1430	0.3180	1.5443	27	0.1204	0.3999	0.4729
5	0.1504	0.3029	1.4293	28	0.1537	0.4038	0.5174
6	0.1536	0.2912	1.2737	29	0.2499	0.4379	0.5783
7	0.1545	0.2819	1.0900	30	0.5959	0.5652	0.7258
8	0.1541	0.2748	0.9225	31	0.5699	0.5692	1.7344
9	0.1531	0.2694	0.7656	32	0.4814	0.5152	2.6426
10	0.1515	0.2656	0.6071	33	0.4459	0.5305	2.6919
11	0.1495	0.2630	0.4579	34	0.4198	0.5374	1.2708
12	0.1469	0.2617	0.3432	35	0.3966	0.5419	0.6529
13	0.1436	0.2615	0.2672	36	0.3746	0.5458	0.5560
14	0.1398	0.2625	0.2130	37	0.3532	0.5499	0.5642
15	0.1353	0.2646	0.2169	38	0.3320	0.5547	0.5993
16	0.1303	0.2682	0.2189	39	0.3110	0.5604	0.6436
17	0.1249	0.2732	0.2260	40	0.2901	0.5672	0.6924
18	0.1194	0.2799	0.2455	41	0.2694	0.5750	0.7443
19	0.1138	0.2883	0.2642	42	0.2489	0.5838	0.7988
20	0.1085	0.2986	0.2831	43	0.2288	0.5935	0.8556
21	0.1037	0.3105	0.3030	44	0.2090	0.6039	0.9145
22	0.0999	0.3241	0.3245	45	0.1896	0.6147	0.9754
23	0.0974	0.3391	0.3481	46	0.1708	0.6255	1.0379
24	0.0968	0.3551	0.3741	47	0.1526	0.6362	1.1020
25	0.0991	0.3715	0.4030	48	0.1351	0.6461	1.1675
26	0.1057	0.3872	0.4356	49	0.1183	0.6550	1.2344

Table S2. The relationship between parameters $\boldsymbol{q}$ and $\boldsymbol{\tau}$ for the 16-node network model

τ	q	τ	q	τ	q	τ	q
1	0.0500	79	0.0344	157	0.0188	235	0.0032
3	0.0496	81	0.0340	159	0.0184	237	0.0028
5	0.0492	83	0.0336	161	0.0180	239	0.0024
7	0.0488	85	0.0332	163	0.0176	241	0.0020
9	0.0484	87	0.0328	165	0.0172	243	0.0016
11	0.0480	89	0.0324	167	0.0168	245	0.0012
13	0.0476	91	0.0320	169	0.0164	247	0.0008
15	0.0472	93	0.0316	171	0.0160	249	0.0004
17	0.0468	95	0.0312	173	0.0156	251	0.0000
19	0.0464	97	0.0308	175	0.0152	253	0.0004
21	0.0460	99	0.0304	177	0.0148	255	0.0008
23	0.0456	101	0.0300	179	0.0144	257	0.0012
25	0.0452	103	0.0296	181	0.0140	259	0.0016
27	0.0448	105	0.0292	183	0.0136	261	0.0020
29	0.0444	107	0.0288	185	0.0132	263	0.0024
31	0.0440	109	0.0284	187	0.0128	265	0.0028

33	0.0436	111	0.0280	189	0.0124	267	0.0032
35	0.0432	113	0.0276	191	0.0120	269	0.0036
37	0.0428	115	0.0272	193	0.0116	271	0.0040
39	0.0424	117	0.0268	195	0.0112	273	0.0044
41	0.0420	119	0.0264	197	0.0108	275	0.0048
43	0.0416	121	0.0260	199	0.0104	277	0.0052
45	0.0412	123	0.0256	201	0.0100	279	0.0056
47	0.0408	125	0.0252	203	0.0096	281	0.0060
49	0.0404	127	0.0248	205	0.0092	283	0.0064
51	0.0400	129	0.0244	207	0.0088	285	0.0068
53	0.0396	131	0.0240	209	0.0084	287	0.0072
55	0.0392	133	0.0236	211	0.0080	289	0.0076
57	0.0388	135	0.0232	213	0.0076	291	0.0080
59	0.0384	137	0.0228	215	0.0072	293	0.0084
61	0.0380	139	0.0224	217	0.0068	295	0.0088
63	0.0376	141	0.0220	219	0.0064	297	0.0092
65	0.0372	143	0.0216	221	0.0060	299	0.0096
67	0.0368	145	0.0212	223	0.0056	301	0.0100
69	0.0364	147	0.0208	225	0.0052	303	0.0104
71	0.0360	149	0.0204	227	0.0048	305	0.0108
73	0.0356	151	0.0200	229	0.0044	307	0.0112
75	0.0352	153	0.0196	231	0.0040	309	0.0116
77	0.0348	155	0.0192	233	0.0036		

Table S3. The relationship between parameters $\boldsymbol{p}$ and $\boldsymbol{\tau}$ for the 18-node network model

τ	p	τ	p	τ	p	τ	p
1	-1.000	49	-0.760	97	-0.520	145	-0.280
3	-0.990	51	-0.750	99	-0.510	147	-0.270
5	-0.980	53	-0.740	101	-0.500	149	-0.260
7	-0.970	55	-0.730	103	-0.490	151	-0.250
9	-0.960	57	-0.720	105	-0.480	153	-0.240
11	-0.950	59	-0.710	107	-0.470	155	-0.230
13	-0.940	61	-0.700	109	-0.460	157	-0.220
15	-0.930	63	-0.690	111	-0.450	159	-0.210
17	-0.920	65	-0.680	113	-0.440	161	-0.200
19	-0.910	67	-0.670	115	-0.430	163	-0.190
21	-0.900	69	-0.660	117	-0.420	165	-0.180
23	-0.890	71	-0.650	119	-0.410	167	-0.170
25	-0.880	73	-0.640	121	-0.400	169	-0.160
27	-0.870	75	-0.630	123	-0.390	171	-0.150
29	-0.860	77	-0.620	125	-0.380	173	-0.140
31	-0.850	79	-0.610	127	-0.370	175	-0.130
33	-0.840	81	-0.600	129	-0.360	177	-0.120
35	-0.830	83	-0.590	131	-0.350	179	-0.110
37	-0.820	85	-0.580	133	-0.340	181	-0.100
39	-0.810	87	-0.570	135	-0.330	183	-0.090
41	-0.800	89	-0.560	137	-0.320	185	-0.080
43	-0.790	91	-0.550	139	-0.310	187	-0.070
45	-0.780	93	-0.540	141	-0.300	189	-0.060
47	-0.770	95	-0.530	143	-0.290		

Table S4. The 2-5 most relevant biometric items for the diagnoses/diseases

Diagnosis/Diseases	Item 1	Item 2	Item 3	Item 4	Item 5
Cardiac arrest	Heart Rate	O2 saturation pulseoxymetry	Level of Consciousness	Dorsal PedPulse	Cough/Deep Breathe
SEPSIS	Temperature	Heart Rate	Respiratory Rate		
intracranial hemorrhage	Heart Rate	Blood pressure systolic	Respiratory Rate		
Subarachnoid hemorrhage	Arterial BP [Systolic]	Arterial BP [Diastolic]			
Pneumonia	WBC	O2 saturation pulseoxymetry			
Congestive heart failure	Heart Rate	Arterial BP [Systolic]	Arterial BP [Diastolic]	Brain Natiuretic Peptide (BNP)	
ALTERED MENTAL STATUS	Respiratory Rate	O2 saturation pulseoxymetry	Heart Rhythm		
Acute respiratory failure	Arterial CO2 Pressure	TCO2 (calc) Arterial	Arterial O2 pressure	Arterial PaCO2	Arterial PaO2
Abdominal pain	Glucose finger stick	WBC	C Reactive Protein (CRP)		
STROKE;TELEMETRY;TRANSIENT ISCHEMIC ATTACK	Non-Invasive Blood Pressure Alarm - Low	Non-Invasive Blood Pressure Alarm - High	Blood Temperature CCO (C)		
Chest pain	Troponin	SpO2	Brain Natiuretic Peptide (BNP)	D-Dimer	
hypotension	NBP [Systolic]	Heart Rate	Heart Rhythm		
Acute posthemorrhagic anemia	Hemoglobin	Heart Rate	Platelet Count	WBC	
Pure hypercholesterolemia	Cholesterol	LDL	HDL	Triglyceride	
Acidosis	PH (Arterial)	PaCO2	HCO3Apache IIValue	Lactic Acid	
Thrombocytopenia	Platelet Count	Packed Red Blood Cells	Prothrombin time		
Cardiogenic shock	Arterial BP [Systolic]	O2 saturation pulseoxymetry	Lactic Acid	PCWP	CI (PiCCO)

Section S3. Supplementary Notes

Supplementary Note S1. Dynamical systems and delay embedding theorem

For a general discrete-time dissipative system, the dynamics can be defined as [2,3]

$$\mathbf{X}^{t+1} = \phi(\mathbf{X}^t), \tag{S1}$$

where $\phi: \mathbb{R}^n \to \mathbb{R}^n$ is a nonlinear map, and its variables are defined in the n-dimensional state space $\mathbf{X}^t = (x_1^t, x_2^t, \dots, x_n^t)'$ at a time point t, where " $'$ " denotes the transpose of a vector, and any time interval between two consecutive time points is equal. After a sufficiently long time, all states converge into a compact manifold $\mathcal{V}$. Denoting $\mathcal{A}$ as the attractor contained in manifold V with the box-counting dimension d, the delay embedding theorem indicates that using only observed long-term data of a single variable can topologically reconstruct the attractor $\mathcal{V}$ of the original high-dimensional system when certain conditions are satisfied. Takens' embedding theorem is stated as follows [2,3].

If $\mathcal{V}$ is an attractor with the box-counting dimension d, for a smooth diffeomorphism $\phi: \mathcal{V} \to \mathcal{V}$ and a smooth function $h: \mathcal{V} \to \mathbb{R}^1$, there is a generic property that the mapping $\Phi_{\phi,h}: \mathcal{V} \to \mathbb{R}^L$ is an embedding when $L > 2d$, that is,

$$\Phi_{\phi,h}(X) = (h(X), h \circ \phi(X), \dots, h \circ \phi^{L-1}(X))'. \tag{S2}$$

where symbol " $\circ$ " is the function composition operation. Generally, the dimension of the original system or the manifold $\mathcal{V}$ is much larger than that of attractor $\mathcal{A}$, i.e., $n \gg d$. In particular, letting $X = \mathbf{X}^t$ and $h(\mathbf{X}^t) = z^t$ where $z^t \in \mathbb{R}^1$, the mapping above has the following form with $\Phi_{\phi,h} = \Phi$ and

$$\Phi(\mathbf{X}^t) = (z^t, z^{t+1}, \dots, z^{t+L-1})' = \mathbf{Z}^t \tag{S3}$$

which is used in the following STI equations (Supplementary Eq. (S3) or main text Eq. (12)). Moreover, since the embedding is a one-to-one mapping, we can derive its conjugate form $\Psi: \mathbb{R}^L \to \mathbb{R}^n$ as $\mathbf{X}^t = \Phi^{-1}(\mathbf{Z}^t) = \Psi(\mathbf{Z}^t)$. Note that $\mathbf{X}^t$ contains n-dimensional variables here.

Supplementary Note S2. Spatiotemporal information (STI) transformation equations

The steady-state or attractor is constrained in a low-dimensional space for a high-dimensional system, which also holds true for most real-world systems. By exploring this low-dimensional feature, spatiotemporal information (STI) transformation[4–6] has been theoretically derived from delay embedding theory [3], which can transform the spatial information of high-dimensional data to the temporal information of any target variable.

Assuming $L > 2d > 0$, where d is the box-counting dimension of attractor $\mathcal{A}$ or manifold $\mathcal{V}$ and L is the number of embeddings, the STI equations can be defined as Supplementary Eq. (S3) or as follows, where $t = 1, 2, \ldots, m$, and m is the length of X.

$$\Phi(\mathbf{X}^t) = \mathbf{Z}^t$$

where $\Phi: \mathbb{R}^n \to \mathbb{R}^L$ is a differentiable function and X is n dimensions. Clearly, the left-hand side of this STI equation is the spatial information of n variables, while the right-hand side is the temporal information of the target variable.

Based on STI transformation, a randomly distributed embedding (RDE) framework has been developed for one-step-ahead prediction from short-term high-dimensional time series [5] by separately constructing many partial STI transformations. Furthermore, the multistep-ahead prediction is performed by using a multilayer neural network and an autoreservoir to represent only the primary STI equation [6].

Supplementary Note S3. Details of the stPCA algorithm

For each observed high-dimensional time series $\mathbf{X}^t = (x_1^t, x_2^t, \ldots, x_n^t)'$ with n variables and $t = 1,2, \ldots, m$, we construct a corresponding delayed vector $\mathbf{Z}^t = (z^t, z^{t+1}, \ldots, z^{t+L-1})'$ by a delay embedding strategy with $L > 1$ as the embedding dimension ($L > 2d > 0$, where d is the box-counting dimension of the attractor of the original high-dimensional system), and the "′" denotes the transpose of a vector. We establish the following equation as a linearized form of transformation $Z = WX$, where X, W and Z are defined as

$$X = \begin{pmatrix} x_1^1 & x_1^2 & \cdots & x_1^m \\ x_2^1 & x_2^2 & \cdots & x_2^m \\ \vdots & \vdots & \ddots & \vdots \\ x_n^1 & x_n^2 & \cdots & x_n^m \end{pmatrix}_{n\times m}, W = \begin{pmatrix} w_{11} & w_{12} & \cdots & w_{1n} \\ w_{21} & w_{22} & & w_{2n} \\ \vdots & \vdots & \ddots & \vdots \\ w_{L1} & w_{L2} & \cdots & w_{Ln} \end{pmatrix}_{L\times n}, Z = \begin{pmatrix} z^1 & z^2 & \cdots & z^m \\ z^2 & z^3 & \cdots & z^{m+1} \\ \vdots & \vdots & \ddots & \vdots \\ z^L & z^{L+1} & \cdots & z^{m+L-1} \end{pmatrix}_{L\times m}.$$

Based on the delay embedding requirement for Z, the last $m-1$ elements in the i-th row are identical to the first $m-1$ elements in the $(i+1)$-th row of the matrix Z. In addition, we have

$$\boldsymbol{W}_i P = (z^{i+1}, z^{i+2}, \cdots, z^{i+m-1}), \quad \text{(S4)}$$

$$\boldsymbol{W}_{i+1} Q = (z^{i+1}, z^{i+2}, \cdots, z^{i+m-1}), \quad \text{(S5)}$$

where P and Q are two submatrices of X, i.e., $P = [\boldsymbol{x}^2\ \boldsymbol{x}^3\ \cdots \boldsymbol{x}^m]$, $Q = [\boldsymbol{x}^1\ \boldsymbol{x}^2\ \cdots \boldsymbol{x}^{m-1}]$, and $\boldsymbol{W}_i = (w_{i1}, w_{i2}, \cdots, w_{in})$, $\boldsymbol{x}^t = (x_1^t, x_2^t, \cdots, x_n^t)'$, and $i = 1, 2, \cdots, L-1$.

From Eq. (S4) and Eq. (S5), the following equation can be obtained:

$$\boldsymbol{W}_i P = \boldsymbol{W}_{i+1} Q, \quad \text{(S6)}$$

---

1. We have a linear transformation $Z = WX$, e.g., $Z_i = W_i X$, based on which the loss function is defined as

$$\underset{W}{\text{minimize}} \quad -(1-\lambda)\sum_{i=1}^{L} \| \boldsymbol{W}_i X \|_2^2 + \lambda \sum_{i=1}^{L-1} \| \boldsymbol{W}_i P - \boldsymbol{W}_{i+1} Q \|_2^2, \quad s.t.\ \sum_{i=1}^{L} \boldsymbol{W}_i \boldsymbol{W}_i^T = 1, \qquad \text{(S7)}$$

where λ is a predetermined regularization parameter.

2. On the basis of the loss function, the Lagrange multiplier is defined as follows:

$$L(W,\Lambda) = -(1-\lambda)\sum_{i=1}^{L} \| \boldsymbol{W}_i X \|_2^2 + \lambda \sum_{i=1}^{L-1} \| \boldsymbol{W}_i P - \boldsymbol{W}_{i+1} Q \|_2^2 + \Lambda\left(\sum_{i=1}^{L} \boldsymbol{W}_i \boldsymbol{W}_i^T - 1\right)$$

$$= -(1-\lambda)\cdot \sum_{i=1}^{L} tr(\boldsymbol{W}_i X X^T \boldsymbol{W}_i^T) + \sum_{i=1}^{L-1} tr((W_i P - W_{i+1}Q)^T (W_i P - W_{i+1} Q)) + \Lambda\left(\sum_{i=1}^{L} \boldsymbol{W}_i \boldsymbol{W}_i^T - 1\right)$$

$$= -(1-\lambda)\cdot \sum_{i=1}^{L} tr(\boldsymbol{W}_i X X^T \boldsymbol{W}_i^T) + \lambda \sum_{i=1}^{L-1} tr(W_i P P^T W_i^T + W_{i+1} Q Q^T W_{i+1}^T - W_i P Q^T W_{i+1}^T - W_{i+1} Q P^T W_i^T) +$$
$$\Lambda_i (W_i W_i^T - 1),$$

where $\Lambda \in \mathbb{R}^{nL \times nL}$ is a Lagrange matrix.

3. Taking the partial derivative of $L(W,\Lambda)$ with respect to W_i and Λ yields

$$\frac{\partial L(W,\Lambda)}{\partial W_1} = -2(1-\lambda)\cdot XX^T W_1^T + \lambda(2PP^T W_1^T - PQ^T W_2^T - (W_2 Q P^T)^T) + 2\Lambda_1 W_1^T$$
$$= -2(1-\lambda)\cdot XX^T W_1^T + 2\lambda \cdot (PP^T W_1^T - PQ^T W_2^T) + 2\Lambda_1 W_1^T$$
$$= -2(((1-\lambda)\cdot XX^T - \lambda PP^T) W_1^T + \lambda PQ^T W_2^T) + 2\Lambda_1 W_1^T \qquad \text{(S8)}$$

$$\frac{\partial L(W,\Lambda)}{\partial W_i} = -2(1-\lambda)\cdot XX^T W_i^T + \lambda((2QQ^T W_i^T - (W_{i-1} P Q^T)^T - QP^T W_{i-1}^T) + (2PP^T W_i^T - PQ^T W_{i+1}^T - (W_{i+1} Q P^T)^T)) + 2\Lambda_i W_i^T$$
$$= -2(1-\lambda) XX^T W_i^T + 2\lambda(PP^T W_i^T + QQ^T W_i^T - QP^T W_{i-1}^T - PQ^T W_{i+1}^T) + 2\Lambda_i W_i^T$$
$$= -2\left(\lambda QP^T W_{k-1}^T + \left((1-\lambda)\cdot XX^T - \lambda PP^T - \lambda QQ^T\right) W_k^T + \lambda PQ^T W_{k+1}^T\right) + 2\Lambda_i W_i^T, \text{ where } 2 \le i \le L-1. \qquad \text{(S9)}$$

$$\frac{\partial L(W,\Lambda)}{\partial W_L} = -2(1-\lambda)\cdot XX^T W_L^T + \lambda(2QQ^T W_L^T - (W_{L-1} P Q^T)^T - QP^T W_{L-1}^T)) + 2\Lambda_L W_L^T \text{i}$$
$$= -2(1-\lambda)\cdot XX^T W_L^T + 2\lambda(QQ^T W - QP^T W_{L-1}^T) + 2\Lambda_L W_L^T$$
$$= -2\lambda\left(QP^T W_{L-1}^T + \left((1-\lambda)\cdot XX^T - \lambda QQ^T\right) W_L^T\right) + 2\Lambda_L W_L^T \qquad \text{(S10)}$$

4. Letting $\frac{\partial L(W,\Lambda)}{\partial W_i} = 0$, we obtain

the following characteristic equation from Eqs. (S8)-(S10):

$$\begin{bmatrix} (1-\lambda)XX^T - \lambda PP^T & \lambda PQ^T & 0 & \cdots & 0 & 0 & 0 \\ \lambda QP^T & (1-\lambda)XX^T - \lambda PP^T - \lambda QQ^T & \lambda PQ^T & \cdots & 0 & 0 & 0 \\ \cdots & \cdots & \cdots & \cdots & \cdots & \cdots & \cdots \\ 0 & 0 & 0 & \cdots & \lambda QP^T & (1-\lambda)XX^T - \lambda PP^T - \lambda QQ^T & \lambda PQ^T \\ 0 & 0 & 0 & \cdots & 0 & \lambda QP^T & (1-\lambda)XX^T - \lambda QQ^T \end{bmatrix}_{(nL)\times(nL)}$$

$$\cdot\begin{bmatrix} W_1^T \\ W_2^T \\ \vdots \\ W_{L-1}^T \\ W_L^T \end{bmatrix}_{(nL)\times 1} = \begin{bmatrix} \Lambda_1 & 0 & 0 & 0 \\ 0 & \Lambda_2 & 0 & 0 \\ \vdots & \vdots & \ddots & \vdots \\ 0 & 0 & \cdots & \Lambda_L \end{bmatrix}_{(nL)\times(nL)} \cdot \begin{bmatrix} W_1^T \\ W_2^T \\ \vdots \\ W_{L-1}^T \\ W_L^T \end{bmatrix}_{(nL)\times 1} \tag{S11}$$

$$H(X)\boldsymbol{V} = \alpha\boldsymbol{V},$$

where H is a function that converts X to a block-tridiagonal matrix, i.e.,

$$H(X) =$$
$$\begin{pmatrix} (1-\lambda)XX^T - \lambda PP^T & \lambda PQ^T & 0 & \cdots & 0 & 0 & 0 \\ \lambda QP^T & (1-\lambda)XX^T - \lambda PP^T - \lambda QQ^T & \lambda PQ^T & \cdots & 0 & 0 & 0 \\ \cdots & \cdots & \cdots & \cdots & \cdots & \cdots & \cdots \\ 0 & 0 & 0 & \cdots & \lambda QP^T & (1-\lambda)XX^T - \lambda PP^T - \lambda QQ^T & \lambda PQ^T \\ 0 & 0 & 0 & \cdots & 0 & \lambda QP^T & (1-\lambda)XX^T - \lambda QQ^T \end{pmatrix},$$

α and $\boldsymbol{V}$ denote a top eigenvalue and eigenvector of $H(X)$.

5. W is obtained by reshaping the vector $\boldsymbol{V}$ by $W = reshape(V) \cdot \alpha$.

6. We obtain

$$\hat{Z} = WX. \tag{S12}$$

7. From Eq. (S12), we let $z^t = \frac{\sum \hat{z}_{i,j}}{k}$, where $i + j = t + 1$, and $i = 1, \dots, L;\ j = 1, \dots, m;\ t = 1, \dots, m + L - 1$, and k is the number of $\hat{z}_{i,j}$. The one-dimensional representation $\boldsymbol{z} = (z^1, z^2, \cdots, z^m, z^{m+1}, \cdots, z^{m+L-1})$ and a Hankel matrix Z can then be easily obtained from $\boldsymbol{z}$.

Supplementary Note S4. Coupled Lorenz systems

To validate the effectiveness of stPCA in capturing the dynamics of a high-dimensional nonlinear system, we consider a series of coupled Lorentz systems [7]. The ith ($i = 1,2, \dots, N$) coupled subsystem is given by

$$\begin{cases} \dot{x}_i = \sigma(t)(y_i - x_i) + Cx_{i-1} \\ \dot{y}_i = \rho x_i - y_i - x_i z_i \\ \dot{z}_i = -\beta z_i + x_i y_i \end{cases}. \tag{S13}$$

The coupling term Cx_{i-1} represents that the ith subsystem is coupled with the $(i-1)$th subsystem via the x component. When $i = 1$, we set $i - 1$ as N so that the system can be closed. We set ρ, β and C to be typical values, i.e., $\rho = 28, \beta = 83, C = 0.1$.

Notably, Supplementary Eq. (S13) is an ordinary Lorenz System (time-invariant system) when $\sigma(t) \equiv 10$, which was used in Fig. 2 of the main text, Supplementary Figs. 1-6 and Supplementary Table 1. When generating the dataset, we set the initial values of $\{x_i(0), y_i(0), z_i(0)\}_{i=1,2,\ldots,N}$ as 0.1 and the time interval Δt as 0.02. We collect data after transient dynamics. In the application, we select different sets of known series, i.e., 10 and 50 points, as known data (Supplementary Figs. 1-6 and Supplementary Table 1). Notably, stPCA uses only the generated time-course datasets generated from Supplementary Eq. (S13) to perform the dimensionality reduction as in the illustrated examples, without using Supplementary Eq. (S13).

Supplementary Note S5. The details of the numerical simulation on multiple-node networks

In this study, we use a sixteen-gene network (see Fig. 3A in the main text) and an eighteen-gene network (see Fig. 3E in the main text) to conduct two numerical simulations and theoretically demonstrate the detection of early warning signals through the stPCA scheme.

Sixteen-node network:

these types of gene regulatory networks are often used to study transcription, translation, diffusion, and translocation processes that affect gene regulatory activities. The following sixteen differential equations represent the gene regulation of sixteen genes in a network where gene regulation is represented in a Michaelis–Menten form, excluding the degradation rates, which are linearly proportional to the concentrations of the corresponding genes.

$$
\begin{cases}
\frac{dz_1(t)}{dt} = \frac{(8-4q)z_2(t)}{15(1+z_2(t))} - 4\left(\frac{1+q}{15}\right)z_1(t) + \zeta_1(t) \\
\frac{dz_2(t)}{dt} = \frac{(4-2q)z_1(t)}{15(1+z_1(t))} - 2\left(\frac{4+q}{15}\right)z_2(t) + \zeta_2(t) \\
\frac{dz_3(t)}{dt} = \frac{4q-10}{15} + \frac{5-2q}{15(1+z_1(t))} + \frac{5-2q}{15(1+z_2(t))} - z_3(t) + \zeta_3(t) \\
\frac{dz_4(t)}{dt} = \frac{(6-2q)z_1(t)}{15(1+z_1(t))} + \frac{(6-2q)z_2(t)}{15(1+z_2(t))} - \frac{6}{5}z_4(t) + \zeta_4(t) \\
\frac{dz_5(t)}{dt} = \frac{4q-14}{15} + \frac{(7-2q)}{15(1+z_1(t))} + \frac{(7-2q)}{15(1+z_2(t))} - \frac{7}{5}z_5(t) + \zeta_5(t) \\
\frac{dz_6(t)}{dt} = \frac{4q-16}{15} + \frac{2(4-q)}{15(1+z_1(t))} + \frac{2(4-q)}{15(1+z_2(t))} - \frac{8}{5}z_6(t) + \zeta_6(t) \\
\frac{dz_7(t)}{dt} = \frac{(9-2q)z_1(t)}{15(1+z_1(t))} + \frac{(9-2q)z_2(t)}{15(1+z_2(t))} - \frac{9}{5}z_7(t) + \zeta_7(t) \\
\frac{dz_8(t)}{dt} = -\frac{13}{15} + \frac{2}{15(1+z_1(t))} + \frac{2}{15(1+z_2(t))} + \frac{2}{5(1+z_6(t))} + \frac{2z_{10}(t)}{5(1+z_{10}(t))} + \frac{3z_{12}(t)}{5(1+z_{12}(t))} \\
\qquad + \frac{z_{15}(t)}{5(1+z_{12}(t))} + \frac{1}{5(1+z_{16}(t))} - 2z_8(t) + \zeta_8(t) \\
\frac{dz_9(t)}{dt} = -1 + \frac{1}{5(1+z_1(t))} + \frac{1}{5(1+z_2(t))} + \frac{3}{5(1+z_6(t))} - \frac{11}{5}z_9(t) + \zeta_9(t) \\
\frac{dz_{10}(t)}{dt} = \frac{3z_{12}(t)}{5(1+z_{12}(t))} - \frac{12}{5}z_{10}(t) + \zeta_{10}(t) \\
\frac{dz_{11}(t)}{dt} = \frac{z_{12}(t)}{4(1+z_{12}(t))} - \frac{13}{5}z_{11}(t) + \zeta_{11}(t) \\
\frac{dz_{12}(t)}{dt} = -\frac{2}{5} + \frac{2z_{15}(t)}{5(1+z_{15}(t))} + \frac{2}{5(1+z_{16}(t))} - \frac{14}{5}z_{12}(t) + \zeta_{12}(t) \\
\frac{dz_{13}(t)}{dt} = -\frac{24}{5} + \frac{1}{1+z_{15}(t)} + \frac{19}{5(1+z_{16}(t))} - 5z_{13}(t) + \zeta_{13}(t) \\
\frac{dz_{14}(t)}{dt} = -\frac{8}{5} + \frac{4}{5(1+z_{10}(t))} + \frac{4}{5(1+z_{12}(t))} - \frac{16}{5}z_{14}(t) + \zeta_{14}(t) \\
\frac{dz_{15}(t)}{dt} = \frac{z_{16}(t)}{10(1+z_{16}(t))} - \frac{7}{2}z_{15}(t) + \zeta_{15}(t) \\
\frac{dz_{16}(t)}{dt} = \frac{z_{15}(t)}{10(1+z_{15}(t))} - \frac{7}{2}z_{16}(t) + \zeta_{16}(t)
\end{cases}
\tag{S14}
$$

where q is a scalar control parameter and $\zeta_i(t)$ $(i = 1, 2, \ldots, 16)$ are Gaussian noises with zero means. $z_i(t)$ $(i = 1, 2, \ldots, 16)$ represents the concentrations of mRNA-i. In Eq. (S9), the degradation rates of mRNAs are $(-4\frac{1+q}{15}, -2\frac{4+q}{15}, -1, -\frac{6}{5}, -\frac{7}{5}, -\frac{8}{5}, -\frac{9}{5}, -2, -\frac{11}{5}, -\frac{12}{5}, -\frac{13}{5}, -\frac{14}{5}, -5, -\frac{16}{5}, -\frac{7}{2}, -\frac{7}{2})$. The stable equilibrium point $\bar{Z} = (\bar{z}_1,\ \bar{z}_2, \ldots,\ \bar{z}_{16})$ =(0, 0, …, 0) exists. The differential equations Eq. (S14) can be transformed into the difference equations $Z(k+1) = f(Z(k), q)$ using the Euler scheme, i.e.,

$$
\begin{cases}
z_1(k+1) = z_1(k) + \left[\frac{(8-4q)z_2(k)}{15(1+z_2(k))} - 4\left(\frac{1+q}{15}\right) z_1(k) + \zeta_1(k)\right]\Delta t \\
z_2(k+1) = z_2(k) + \left[\frac{(4-2q)z_1(k)}{15(1+z_1(k))} - 2\left(\frac{4+q}{15}\right) z_2(k) + \zeta_2(k)\right]\Delta t \\
z_3(k+1) = z_3(k) + \left[\frac{4q-10}{15} + \frac{5-2q}{15(1+z_1(k))} + \frac{5-2q}{15(1+z_2(k))} - z_3(k) + \zeta_3(k)\right]\Delta t \\
z_4(k+1) = z_4(k) + \left[\frac{(6-2q)z_1(k)}{15(1+z_1(k))} + \frac{(6-2q)z_2(k)}{15(1+z_2(k))} - \frac{6}{5}z_4 + \zeta_4(k)\right]\Delta t \\
z_5(k+1) = z_5(k) + \left[\frac{4q-14}{15} + \frac{(7-2q)}{15(1+z_1(k))} + \frac{(7-2q)}{15(1+z_2(k))} - \frac{7}{5}z_5(k) + \zeta_5(k)\right]\Delta t \\
z_6(k+1) = z_6(k) + \left[\frac{4q-16}{15} + \frac{2(4-q)}{15(1+z_1(k))} + \frac{2(4-q)}{15(1+z_2(k))} - \frac{8}{5}z_6(k) + \zeta_6(k)\right]\Delta t \\
z_7(k+1) = z_7(k) + \left[\frac{(9-2q)z_1(k)}{15(1+z_1(k))} + \frac{(9-2q)z_2(k)}{15(1+z_2(k))} - \frac{9}{5}z_7 + \zeta_7(k)\right]\Delta t \\
z_8(k+1) = z_8(k) + [-\frac{13}{15} + \frac{2}{15(1+z_1(k))} + \frac{2}{15(1+z_2(k))} + \frac{2}{5(1+z_6(k))} + \frac{2z_{10}(k)}{5(1+z_{10}(k))} \\
\qquad + \frac{3z_{12}(k)}{5(1+z_{12}(k))} + \frac{z_{15}(k)}{5(1+z_{15}(k))} + \frac{1}{5(1+z_{16}(k))} - 2z_8(k) + \zeta_8(k)]\Delta t \\
z_9(k+1) = z_9(k) + \left[-1 + \frac{1}{5(1+z_1(k))} + \frac{1}{5(1+z_2(k))} + \frac{3}{5(1+z_6(k))} - \frac{11}{5}z_9(k) + \zeta_9(k)\right]\Delta t \\
z_{10}(k+1) = z_{10}(k) + \left[\frac{3z_{12}(k)}{5(1+z_{12}(k))} - \frac{12}{5}z_{10}(k) + \zeta_{10}(k)\right]\Delta t \\
z_{11}(k+1) = z_{11}(k) + \left[\frac{z_{12}(k)}{4(1+z_{12}(k))} - \frac{13}{5}z_{11}(k) + \zeta_{11}(k)\right]\Delta t \\
z_{12}(k+1) = z_{12}(k) + \left[-\frac{2}{5} + \frac{2z_{15}(k)}{5(1+z_{15}(k))} + \frac{2}{5(1+z_{16}(k))} - \frac{14}{5}z_{12}(k) + \zeta_{12}(k)\right]\Delta t \\
z_{13}(k+1) = z_{13}(k) + \left[-\frac{24}{5} + \frac{1}{1+z_{15}(k)} + \frac{19}{5(1+z_{16}(k))} - 5z_{13}(k) + \zeta_{13}(k)\right]\Delta t \\
z_{14}(k+1) = z_{14}(k) + \left[-\frac{8}{5} + \frac{4}{5(1+z_{10}(k))} + \frac{4}{5(1+z_{12}(k))} - \frac{16}{5}z_{14}(k) + \zeta_{14}(k)\right]\Delta t \\
z_{15}(k+1) = z_{15}(k) + \left[\frac{z_{16}(k)}{10(1+z_{16}(k))} - \frac{7}{2}z_{15}(k) + \zeta_{15}(k)\right]\Delta t \\
z_{16}(k+1) = z_{16}(k) + \left[\frac{z_{15}(k)}{10(1+z_{15}(k))} - \frac{7}{2}z_{16}(k) + \zeta_{16}(k)\right]\Delta t
\end{cases}
\tag{S15}
$$

with a small time interval Δt. Note that Z(k) is the vector of Z(t) at time instant $k\Delta t$. We denote the Jacobian matrix of Eq. (S15) as $J = \frac{\partial f(Z;q)}{\partial Z} \mid_{Z=\bar{Z}}$, where

$$J = \mathrm{e}^{\Delta t \cdot A} \tag{S16}$$

with

$$
A=\begin{bmatrix}
\frac{-4-4q}{15} & \frac{8-4q}{15} & 0 & 0 & 0 & 0 & 0 & 0 & 0 & 0 & 0 & 0 & 0 & 0 & 0 & 0 \\
\frac{4-2q}{15} & \frac{-8-2q}{15} & 0 & 0 & 0 & 0 & 0 & 0 & 0 & 0 & 0 & 0 & 0 & 0 & 0 & 0 \\
\frac{-5+2q}{15} & \frac{-5+2q}{15} & -1 & 0 & 0 & 0 & 0 & 0 & 0 & 0 & 0 & 0 & 0 & 0 & 0 & 0 \\
\frac{6-2q}{15} & \frac{6-2q}{15} & 0 & -\frac{6}{5} & 0 & 0 & 0 & 0 & 0 & 0 & 0 & 0 & 0 & 0 & 0 & 0 \\
\frac{-7+2q}{15} & \frac{-7+2q}{15} & 0 & 0 & -\frac{7}{5} & 0 & 0 & 0 & 0 & 0 & 0 & 0 & 0 & 0 & 0 & 0 \\
\frac{-8+2q}{15} & \frac{-8+2q}{15} & 0 & 0 & 0 & -\frac{8}{5} & 0 & 0 & 0 & 0 & 0 & 0 & 0 & 0 & 0 & 0 \\
\frac{9-2q}{15} & \frac{9-2q}{15} & 0 & 0 & 0 & 0 & -\frac{9}{5} & 0 & 0 & 0 & 0 & 0 & 0 & 0 & 0 & 0 \\
-\frac{2}{15} & -\frac{2}{15} & 0 & 0 & 0 & -\frac{2}{5} & 0 & -2 & 0 & \frac{2}{5} & 0 & \frac{3}{5} & 0 & \frac{1}{5} & 0 & -\frac{1}{5} \\
-\frac{1}{5} & -\frac{1}{5} & 0 & 0 & 0 & -\frac{3}{5} & 0 & 0 & -\frac{11}{5} & 0 & 0 & 0 & 0 & 0 & 0 & 0 \\
0 & 0 & 0 & 0 & 0 & 0 & 0 & 0 & 0 & -\frac{12}{5} & 0 & \frac{3}{5} & 0 & 0 & 0 & 0 \\
0 & 0 & 0 & 0 & 0 & 0 & 0 & 0 & 0 & 0 & -\frac{13}{5} & \frac{1}{4} & 0 & 0 & 0 & 0 \\
0 & 0 & 0 & 0 & 0 & 0 & 0 & 0 & 0 & 0 & 0 & -\frac{14}{5} & 0 & 0 & \frac{2}{5} & -\frac{2}{5} \\
0 & 0 & 0 & 0 & 0 & 0 & 0 & 0 & 0 & 0 & 0 & 0 & -5 & 0 & -1 & \frac{19}{5} \\
0 & 0 & 0 & 0 & 0 & 0 & 0 & 0 & 0 & \frac{4}{5} & 0 & -\frac{4}{5} & 0 & -\frac{16}{5} & 0 & 0 \\
0 & 0 & 0 & 0 & 0 & 0 & 0 & 0 & 0 & 0 & 0 & 0 & 0 & 0 & -\frac{7}{2} & \frac{1}{10} \\
0 & 0 & 0 & 0 & 0 & 0 & 0 & 0 & 0 & 0 & 0 & 0 & 0 & 0 & \frac{1}{10} & -\frac{7}{2}
\end{bmatrix}
$$

We obtain sixteen distinct eigenvalues$(0.67^q, 0.45, 0.37, 0.30, 0.25, 0.20, 0.17, 0.14, 0.11, 0.09, 0.07, 0.06, 0.05, 0.04, 0.033, 0.027)$ from Eq. (S16), by taking $\Delta t = 1$. Obviously, there is a critical value $q_c = 0$, where the system loses stability and undergoes a critical transition. We aim to detect early warning signals that reveal the critical transition as the control parameter q approaches a critical value of 0.

Following the stPCA method, for each simulation trial, we use the 10 samples generated when the control parameter q is far from the critical value $q_c = 0$ (e.g., $q \in \{-0.6, -0.58, -0.56, -0.54, -0.52, -0.5, -0.48, -0.46, -0.44, -0.42\}$) as the reference samples. Then, each simulation trial is based on the single sample $\{z_1, z_2, \dots, z_{16}\}_q$ derived for each parameter value $q \in [-0.4, 0.2]$. In the main text, the parameter τ simply corresponds to the index of q (Table S2), and facilitates the mapping with time points in the time series data.

Eighteen-node network:

The following 18 differential equations represent the interactions of 18 variables in a network where the interactions, excluding the degradation rates, are represented in a Michaelis–Menten form.

$$
\begin{cases}
\frac{dx_1(t)}{dt} = \frac{-5p-1}{5} + \frac{3x_1(t)}{1+x_1(t)} + \frac{x_2(t)}{1+x_2(t)} - 100\left(\frac{x_1(t)}{1+x_1(t)}\right)^3 + \eta_1(t) \\
\frac{dx_2(t)}{dt} = \frac{(8-4p)x_3(t)}{15(1+x_3(t))} - 4\left(\frac{1+p}{15}\right)x_2(t) + \eta_2(t) \\
\frac{dx_3(t)}{dt} = \frac{(4-2p)x_2(t)}{15(1+x_1(t))} - 2\left(\frac{4+p}{15}\right)x_3(t) + \eta_3(t) \\
\frac{dx_4(t)}{dt} = \frac{4p-10}{15} + \frac{5-2p}{15(1+x_2(t))} + \frac{5-2p}{15(1+x_3(t))} - x_4(t) + \eta_4(t) \\
\frac{dx_5(t)}{dt} = \frac{(6-2p)x_2(t)}{15(1+x_1(t))} + \frac{(6-2p)x_3(t)}{15(1+x_3(t))} - \frac{6}{5}x_5(t) + \eta_5(t) \\
\frac{dx_6(t)}{dt} = \frac{4p-14}{15} + \frac{(7-2p)}{15(1+x_2(t))} + \frac{(7-2\text{[illegible]})}{15(1+x_3(t))} - \frac{7}{5}x_6(t) + \eta_6(t) \\
\frac{dx_7(t)}{dt} = \frac{4p-16}{15} + \frac{2(4-p)}{15(1+x_2(t))} + \frac{2(4-p)}{15(1+x_3(t))} - \frac{8}{5}x_7(t) + \eta_7(t) \\
\frac{dx_8(t)}{dt} = \frac{(9-2p)x_2(t)}{15(1+x_2(t))} + \frac{(9-2p)x_3(t)}{15(1+x_3(t))} - \frac{9}{5}x_8(t) + \eta_8(t) \\
\frac{dx_9(t)}{dt} = -\frac{13}{15} + \frac{2}{15(1+x_2(t))} + \frac{2}{15(1+x_3(t))} + \frac{2}{5(1+x_7(t))} + \frac{2x_{11}(t)}{5(1+x_{11}(t))} + \frac{3x_{13}(t)}{5(1+x_{13}(t))} \\
\qquad + \frac{x_{16}(t)}{5(1+x_{16}(t))} + \frac{1}{5(1+x_{17}(t))} - 2x_9(t) + \eta_9(t) \\
\frac{dx_{10}(t)}{dt} = -1 + \frac{1}{5(1+x_2(t))} + \frac{1}{5(1+x_3(t))} + \frac{3}{5(1+x_7(t))} - \frac{11}{5}x_{10}(t) + \eta_{10}(t) \\
\frac{dx_{11}(t)}{dt} = \frac{3x_{13}(t)}{5(1+x_{13}(t))} - \frac{12}{5}x_{11}(t) + \eta_{11}(t) \\
\frac{dx_{12}(t)}{dt} = \frac{x_{13}(t)}{4(1+x_{13}(t))} - \frac{13}{5}x_{12}(t) + \eta_{12}(t) \\
\frac{dx_{13}(t)}{dt} = -\frac{2}{5} + \frac{2x_{16}(t)}{5(1+x_{16}(t))} + \frac{2}{5(1+x_{17}(t))} - \frac{14}{5}x_{13}(t) + \eta_{13}(t) \\
\frac{dx_{14}(t)}{dt} = \frac{6x_{17}(t)}{5(1+x_{17}(t))} - 3x_{14}(t) + \eta_{14}(t) \\
\frac{dx_{15}(t)}{dt} = -\frac{8}{5} + \frac{4}{5(1+x_{11}(t))} + \frac{4}{5(1+x_{13}(t))} - \frac{16}{5}x_{15}(t) + \eta_{15}(t) \\
\frac{dx_{16}(t)}{dt} = \frac{x_{17}(t)}{10(1+x_{17}(t))} - \frac{7}{2}x_{16}(t) + \eta_{16}(t) \\
\frac{dx_{17}(t)}{dt} = \frac{x_{16}(t)}{10(1+x_{16}(t))} - \frac{7}{2}x_{17}(t) + \eta_{17}(t) \\
\frac{dx_{18}(t)}{dt} = \frac{x_1(t)}{1+x_1(t)} + \frac{x_{11}(t)}{15(1+x_{11}(t))} + \frac{x_{15}(t)}{12(1+x_{15}(t))} - x_{18}(t) + \eta_{18}(t)
\end{cases}
\tag{S17}
$$

where p is a scalar control parameter ranging from -1 to 1 and $\eta_i(t)$ (i = 1; 2, …, 18) are Gaussian noises with zero means and covariances κ_{ij}= Cov(η_i; η_j). Here, we set the amplitude κ_{ii} of η_i as 1. x_i (i = 1, …, 18) represents the value of variable i. There is a stable equilibrium when the parameter p is smaller than the critical value P_c= 0. When p exceeds P_c = 0, there is a state transition of the system (see Fig. 3 in the main text). The differential equations Eq. (S16) can be transformed into the difference equations X(k +1) = f(X(k); P) using the Euler scheme [8], with a small time interval Δt = 0.01 when $p \in$ [-1; 1]. Note that X(k) is the vector of X(t) at time instant k Δt. By using this system, we simulate the data and apply the stPCA scheme to identify the pretransition state, as shown in Fig. 3 in the main text.

Notably, the kinetic model has nothing to do with the application of the scheme on real-world data. Model Eq. (S17) relates only to a numerical simulation that is totally irrelevant to real-data applications. In the main

text, the parameter τ simply corresponds to the index of p (Table S3), and facilitates the mapping with time points in the time series data.

Supplementary Note S6. Evaluation Metrics

Besides, to evaluate the ICU discharge performance for a specific method, the following evaluation metrics were employed [9].

1. True Positives (TP)/Recall: The number of cases in which a method yielded an out-of-ICU decision and the patient was discharged within 5 hours or all the most relevant indicators thereafter were within the normal range.
2. True Negative (TN): The number of cases in which a method did not yield an out-of-ICU decision and no patients were discharged from the ICU during the postintervention period.
3. False Positive (FP): The number of cases in which a method yielded an out-of-ICU decision, but no patients were discharged from the ICU during the postintervention period.
4. False Negative (FN): The number of cases in which a method did not yield an out-of-ICU decision, but a patient was discharged from the ICU during the postintervention period.

Based on the indicators above, some other assessment metrics are derived. The TPR (true positive rate), i.e., sensitivity or recall, refers to the ratio of true positive cases to all positive cases, while the TNR (true negative rate), i.e., specificity, refers to the ratio between the true negative cases and all negative cases. In addition, precision is the ratio between the true positive cases and all positive cases, accuracy represents the ratio between the true positive and negative cases and all positive and negative cases, and the FPR (false positive rate) is the ratio between the number of negative events incorrectly categorized as positive (false positives) and the total number of actual negative events. In addition, the F1-score is the average of TPR, precision and accuracy, while the Error is the ratio between false positive and negative cases to all positive and negative cases, in contrast to Accuracy.

These metrics can be mathematically represented as:

$$\mathrm{TPR/Recall} = \frac{\mathrm{TP}}{\mathrm{TP+FN}}, \tag{S18}$$

$$\mathrm{TNR} = \frac{\mathrm{TN}}{\mathrm{FP+TN}}, \tag{S19}$$

$$\mathrm{Precision} = \frac{\mathrm{TP}}{\mathrm{TP+FP}}, \tag{S20}$$

$$\mathrm{Accuracy} = \frac{\mathrm{TP+TN}}{\mathrm{P+N}}, \tag{S21}$$

$$\mathrm{Error} = \frac{\mathrm{FP+FN}}{\mathrm{P+N}}, \tag{S22}$$

$$\text{FPR} = \frac{\text{FP}}{\text{FP+TN}}, \tag{S23}$$

$$\text{F1\_score} = \frac{2*\text{Recall}*\text{Precision}}{\text{Recall+Precision}}. \tag{S24}$$

These evaluation metrics are derived from four components, namely, TP, FP, TN and FN. As each indicator emphasizes a specific aspect of the algorithm's performance, we attempted to illustrate every aspect of the performance of the algorithm by utilizing all these measures.

Based on these metrics, we illustrated the decision-making process of stPCA (and other comparison methods) for a possible discharge and determined the TP, TN, FP, and FN events.

When $Decision(t) = 1$, if the patient is discharged within 5 hours or all the most relevant indicators thereafter are within the normal range, then this decision is considered a true positive (TP); otherwise, it is a false-positive (FP). When $Decision(t) = 0$, if the patient is currently receiving treatment in the ICU, then this decision is considered a true negative (TN); otherwise, it is considered a false negative (FN).

Supplementary Note S7. Discrete Fréchet distance

A $curve$ is defined as a continuous mapping $f : [a, b] \to V$, where $a, b \in \Re$ and $a \leq b$ and (V, d) is a metric space.

Given two curves $f : [a, b] \to V$ and $g : [a', b'] \to V$,, their $Fr\acute{e}chet\ distance$ is defined as

$$\delta_F(f, g) = \inf_{\alpha,\beta} \max_{t\in[0,1]} d(f(\alpha(t)), g(\beta(t))), \tag{S25}$$

where α (resp.β) is an arbitrary continuous nondecreasing function from [0,1]onto $[a, b]$ (resp. $[a', b']$)[10].

In calculating the Fréchet distance between arbitrary curves, one typically approximates the curves by polygonal curves. A $polygonal\ curve$ is a curve $P : [0, n] \to V$, where n is a positive integer, such that for each $i \in \{0,1, \dots, n-1\}$, the restriction of P to the interval $[i, i+1]$ is affine, that is, $P(i+\lambda) = (1-\lambda)P(i) + \lambda P(i+1)$.

Let $P : [0, n] \to V$ be a polygonal curve. We denote the sequence $(P(0), P(1), \dots, P(n))$ of the endpoints of the line segments of P by $\sigma(P)$. Let P and Q be polygonal curves and $\sigma(P) = (u_1, \dots, u_p)$ and $\sigma(Q) = (v_1, \dots, v_q)$ be the corresponding sequences. A $coupling$ L between P and Q is a sequence

$$(u_{a_1}, v_{b_1}), (u_{a_2}, v_{b_2}), \dots, (u_{a_m}, v_{b_m})$$

of distinct pairs from $\sigma(P) \times \sigma(Q)$ such that $a_1 = 1, b_1 = 1, a_m = p$, $b_m = q$, and for all $i = 1, \dots, q$ we have $a_{i+1} = a_i$ or $a_{i+1} = a_i + 1$, and $b_{i+1} = b_i$ or $b_{i+1} = b_i + 1$. Thus, a coupling must respect the order of the points in P and Q. The $length$ $||L||$ of the coupling L is the length of the longest link in L, that is,

$$||L|| = \max_{i=1,\dots,m} d(u_{a_i}, v_{b_i}). \tag{S26}$$

Given polygonal curves P and Q, their $discrete\ Fr\acute{e}chet\ distance$ is defined as

$$\delta_{dF}(P, Q) = \min\{||L|| \mid L \text{ is a coupling between } P \text{ and } Q\}. \tag{S27}$$

Supplementary Note S8. Dynamic time warping

In time series analysis, dynamic time warping [11] (DTW) is an algorithm for measuring similarity between two temporal sequences, which may vary in speed. For instance, similarities in walking could be detected using DTW, even if one person is walking faster than the other or if there are accelerations and decelerations during an observation. DTW has been applied to temporal sequences of video, audio, and graphics data — indeed, any data that can be transformed into a one-dimensional sequence can be analyzed with DTW. A well-known application has been using DTW with automatic speech recognition to handle different speaking speeds. Other applications include the use of DTW in speaker recognition and online signature recognition, as well as in partial shape matching.

In general, DTW calculates an optimal match between two given sequences (e.g., time series) with certain restrictions and rules:

Every index from the first sequence must be matched with one or more indices from the other sequence, and vice versa.

- The first index from the first sequence must be matched with the first index from the other sequence (but it does not have to be its only match)
- The last index from the first sequence must be matched with the last index from the other sequence (but it does not have to be its only match)
- The mapping of the indices from the first sequence to indices from the other sequence must be monotonically increasing, and vice versa, i.e., in this formulation, the number of possible matches is the Delannoy number.

The optimal match is the match that satisfies all the restrictions and rules and that has the minimal cost, where the cost is computed as the sum of absolute differences, for each matched pair of indices, between their values.

The sequences are "warped" nonlinearly in the time dimension to determine a measure of their similarity independent of certain nonlinear variations in the time dimension. This sequence alignment method is often used in time series classification. Although DTW measures a distance-like quantity between two given sequences, it does not guarantee that the triangle inequality holds.

In addition to a similarity measure between the two sequences, a "warping path" is produced. By warping according to this path, the two signals may be temporally aligned time. In this signal, an original set of points X (original) and Y (original) is transformed to X (warped) and Y (warped). This enables applications in genetic sequence and audio synchronization. In a related technique, sequences of varying speeds can be averaged using this technique; see the average sequence section for details.

Supplementary Note S9. Methods for comparison

In this study, we compared the performance of stPCA with that of the following methods.

- Principal Component Analysis (PCA) [12]. PCA is a widely used linear dimensionality reduction method that employs orthogonal transformations to project high-dimensional data into a lower-dimensional space. Its objective is to find a set of new features known as principal components, arranged in descending order of variance, to retain as much information as possible while reducing dimensions. PCA is particularly effective when datasets contain redundant information or when noise reduction is desired.
- Locally Linear Embedding (LLE) [13]. LLE is a nonlinear dimensionality reduction method that preserves the local geometry of data by reconstructing each data point as a weighted sum of its neighbors. It excels in capturing nonlinear features of data, especially for manifold data. LLE's key idea is that each data point can be linearly represented by its nearest neighbors, preserving local relationships.
- t-Distributed stochastic neighbor embedding (t-SNE) [14]. t-SNE is a nonlinear dimensionality reduction technique that maps high-dimensional data into a lower-dimensional space while emphasizing the preservation of local structures and clustering patterns. It is widely used for visualization and cluster analysis, especially in uncovering hidden structures within high-dimensional data. t-SNE uses probability distributions to capture the similarity between data points, mapping similar points to nearby positions in the lower-dimensional space.
- Multidimensional Scaling (MDS) [15]. MDS is a distance-based dimensionality reduction method aimed at preserving pairwise distances between data points. It maps high-dimensional data to a lower-dimensional space in such a way that the distances between points in the lower-dimensional space closely reflect the distance relationships in the original data. MDS is suitable for maintaining relative distances between data points, such as in visualization.
- Isometric Feature Mapping (ISOMAP) [16]. ISOMAP is a nonlinear dimensionality reduction method that approximates geodesic distances between data points on a manifold. It is capable of handling nonlinear manifold data, such as surfaces and curved structures. ISOMAP's key concept involves connecting data points using the shortest path distance to capture manifold structures.
- Autoencoder (AE) [17]. AE is a neural network-based dimensionality reduction method that learns a compressed representation of data by encoding and decoding it through a bottleneck layer. It consists of an encoder and a decoder, where the encoder maps high-dimensional data to a lower-dimensional space, and the decoder reconstructs it to the original dimension. AE is useful for unsupervised learning and feature learning, as well as uncovering latent structures in data.
- Variational Autoencoder (VAE) [18]. VAE is an extension of AE that introduces probabilistic distributions to model the latent space. It allows for sampling in the latent space and generates new data points through the decoder, making it suitable for generative tasks and flexible data representations.

- MAnifold Representation Basis LEarning (MARBLE) [19]. It is a fully unsupervised geometric deep learning framework for the data-driven representation of non-linear dynamics, and aims to find a similarity-preserving map from the local flow fields (LFFs) to a shared latent space.
- Charts and Atlases for Nonlinear Data-Driven Dynamics on Manifolds（CANDyMan) [20]. It employs k-means clustering in state space to partition the data into patches and then expands each cluster along the graph using KNN, resulting in overlapping coordinate domains. Next, it learns the coordinate maps and their inverses of an autoencoder. It estimates the dimension of the underlying manifold by monitoring the reconstruction error of the autoencoder as a function of the latent dimension.
- Hankel alternative view of Koopman (HAVOK) [21]. It combines delay embedding and Koopman theory to decompose chaotic dynamics into a linear model in the leading delay coordinates with forcing by low-energy delay coordinates. Dynamic mode decomposition (DMD) is adopted to approximate the Koopman operator with a best-fit linear model advancing spatial measurements from one time to the next.

Supplementary Note S10. The comparisons of time complexity between DNN methods and stPCA

stPCA: the main time cost comes from solving the dominant eigenvalue and a corresponding eigenvector from a block-tridiagonal matrix $H(X)$. Utilizing the tridiagonal matrix algorithm [22], the time complexity is $O(nL)$.

MARBLE: if implemented parallelly, the diffusion layer has a complexity of $O(1)$, the computation of filters and concatenation of derivatives is $O(p)$, and training the deep neural network is $O(N_{it}Nnl)$, where p is the derivative order. Thus, the overall time complexity is $O(1) + O(p) + O(N_{it}Nnl) \approx O(N_{it}Nnl)$.

CANDyMan: time complexity for training an atlas of charts and corresponding dynamics: create K-nearest neighbours graph: $O(nN)$; k-mean cluster: $O(N_{it}Nkn)$; training the autoencoder: $O(N_{it}kN_snl)$; and the overall time complexity for training is $O(nN) + O(N_{it}Nkn) + O(N_{it}kN_snl) \approx O(N_{it}kN_snl)$;
Time complexity for evolving an initial condition forward: the time complexity is $O(nN) + O(T) \approx O(nN)$ where T is the number of steps.

HAVOK: the main time cost comes from DMD, so the overall time complexity is $O(min\{mn\text{^}2, m\text{^}2n\} + r)$, where r is the rank of input time series.

It is clear that $O(nL) < O(N_{it}kN_snl)$, $O(nL) < O(N_{it}Nnl)$, and $O(nL) < O(min\{mn^2, m^2n\} + r)$, since delay embedding dimension $L < n$, $L < N_{it}$, $L < m$. Additionally, training a deep neural network

typically requires a large sample size N. Therefore, the time complexity O(stPCA) < O(CANDyMan), O(stPCA) < O(MARBLE), and O(stPCA) < O(HAVOK)

Note: n is the dimensional number of original data, m is the total time points of input data, L is the number of delay embeddings, N_{it} is the iteration number in the training process of a neural network, k is the patch number of CANDyMan, N is the total sample number of original data, N_s is the average number of nodes/samples in each patch, l is the number of the neural network layers.

References

1. Fletcher, R. *Practical Methods of Optimization*. (John Wiley & Sons, 2000).
2. Takens, F. Detecting strange attractors in turbulence. in *Dynamical systems and turbulence, Warwick 1980* 366–381 (Springer, 1981).
3. Sauer, T., Yorke, J. A. & Casdagli, M. “Embedology,” Journal of Statistical Physics. (1991).
4. Ma, H., Zhou, T., Aihara, K. & Chen, L. Predicting time series from short-term high-dimensional data. *International Journal of Bifurcation and Chaos* **24**, 1430033 (2014).
5. Ma, H., Leng, S., Aihara, K., Lin, W. & Chen, L. Randomly distributed embedding making short-term high-dimensional data predictable. *Proceedings of the National Academy of Sciences* **115**, E9994–E10002 (2018).
6. Chen, C. *et al.* Predicting future dynamics from short-term time series by anticipated learning machine. *National Science Review* (2020).
7. Curry, J. H. A generalized Lorenz system. *Communications in Mathematical Physics* **60**, 193–204 (1978).
8. Protter, P. & Talay, D. The Euler scheme for Lévy driven stochastic differential equations. *The Annals of Probability* 393–423 (1997).
9. Hossin, M. & Sulaiman, M. N. A review on evaluation metrics for data classification evaluations. *International journal of data mining & knowledge management process* **5**, 1 (2015).
10. Agarwal, P. K., Avraham, R. B., Kaplan, H. & Sharir, M. Computing the discrete Fréchet distance in subquadratic time. *SIAM Journal on Computing* **43**, 429–449 (2014).

11. Müller, M. Dynamic time warping. *Information retrieval for music and motion* 69–84 (2007).
12. Bro, R. & Smilde, A. K. Principal component analysis. *Analytical methods* **6**, 2812–2831 (2014).
13. Roweis, S. T. & Saul, L. K. Nonlinear dimensionality reduction by locally linear embedding. *science* **290**, 2323–2326 (2000).
14. Van der Maaten, L. & Hinton, G. Visualizing data using t-SNE. *Journal of machine learning research* **9**, (2008).
15. Buja, A. *et al.* Data visualization with multidimensional scaling. *Journal of computational and graphical statistics* **17**, 444–472 (2008).
16. Tenenbaum, J. B., Silva, V. de & Langford, J. C. A global geometric framework for nonlinear dimensionality reduction. *science* **290**, 2319–2323 (2000).
17. Kramer, M. A. Nonlinear principal component analysis using autoassociative neural networks. *AIChE journal* **37**, 233–243 (1991).
18. Kingma, D. P. & Welling, M. An introduction to variational autoencoders. *Foundations and Trends® in Machine Learning* **12**, 307–392 (2019).
19. Gosztolai, A., Peach, R. L., Arnaudon, A., Barahona, M. & Vandergheynst, P. Interpretable statistical representations of neural population dynamics and geometry. Preprint at http://arxiv.org/abs/2304.03376 (2024).
20. Floryan, D. & Graham, M. D. Data-driven discovery of intrinsic dynamics. *Nature Machine Intelligence* **4**, 1113–1120 (2022).
21. Brunton, S. L., Brunton, B. W., Proctor, J. L., Kaiser, E. & Kutz, J. N. Chaos as an intermittently forced linear system. *Nature communications* **8**, 19 (2017).
22. Lee, W. T. Tridiagonal matrices: Thomas algorithm. *MS6021, Scientific Computation, University of Limerick* (2011).
